# Heterocyclic Electrochemistry: Renewable Electricity in the Construction of Heterocycles

**DOI:** 10.1021/acsomega.2c07378

**Published:** 2023-02-01

**Authors:** Samina Aslam, Najoua Sbei, Sadia Rani, Manal Saad, Aroog Fatima, Nisar Ahmed

**Affiliations:** † Department of Chemistry, The Women University Multan, Multan60000, Pakistan; # The Department of Chemistry, University of Oxford, 12 Mansfield Road, Oxford, OX1 3TA, U.K.; ‡ School of Chemistry, Cardiff University, Main Building Park Place, Cardiff, CF10 3AT, United Kingdom; § Institute of Nanotechnology, Karlsruhe Institute of Technology, EggensteinLeopoldshafen, 76344KarlsruheGermany

## Abstract

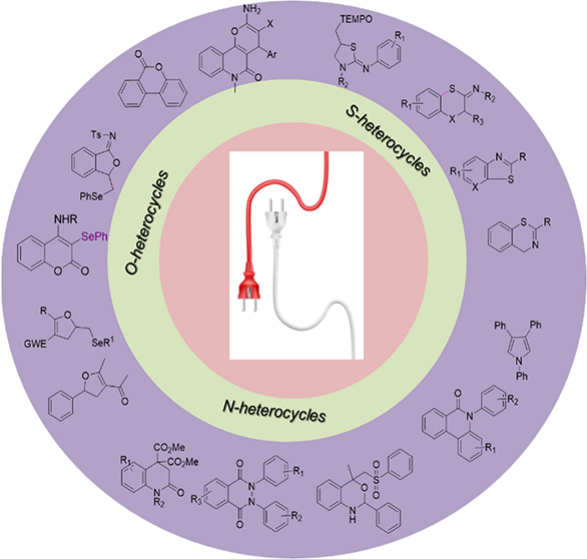

Numerous applications in the realm of biological exploration and drug synthesis can be found in heterocyclic chemistry, which is a vast subject. Many efforts have been developed to further improve the reaction conditions to access this interesting family to prevent employing hazardous ingredients. In this instance, it has been stated that green and environmentally friendly manufacturing methodologies have been introduced to create N-, S-, and O-heterocycles. It appears to be one of the most promising methods to access these types of compounds avoiding use of stoichiometric amounts of oxidizing/reducing species or precious metal catalysts, in which only catalytic amounts are sufficient, and it represent an ideal way of contributing toward the resource economy. Thus, renewable electricity provides clean electrons (oxidant/reductant) that initiate a reaction cascade via producing reactive intermediates that facilitate in building new bonds for valuable chemical transformations. Moreover, electrochemical activation using metals as catalytic mediators has been identified as a more efficient strategy toward selective functionalization. Thus, indirect electrolysis makes the potential range more practical, and less side reactions can occur. The latest developments in using an electrolytic strategy to create N-, S-, and O-heterocycles are the main topic of this mini review, which was documented over the last five years.

## 1 Introduction

The organic chemistry field has always been fascinated by the synthesis and modification of heterocyclic structures. In contrast, formulations are frequently conducted in environments that are contradictory to the existence of specific functional groups in the substrates, such as those that call for the employment of poisonous substances like bases or acids or that occasionally demand extreme temperatures in order to produce heterocyclic systems.

**Figure 1:**
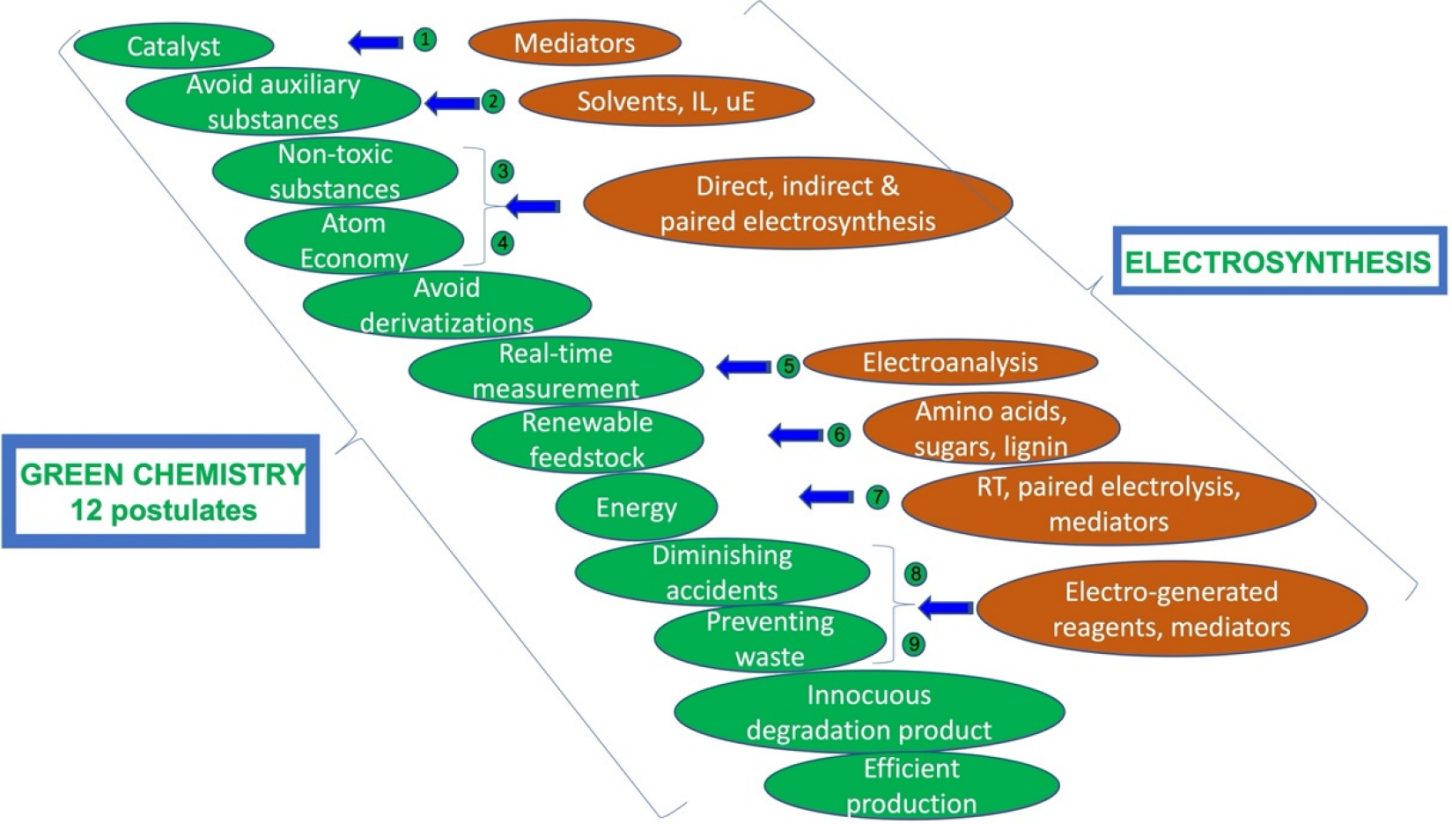
Correlation of electrosynthesis with green chemistry postulates.^9^ Adapted with permission from ref (9). Copyright 2010, The Royal Society of Chemistry.

The major objective of scientists has been concentrated on the creation of environmentally friendly and long-term technologies without introducing noxious materials in regard to growth with economic and environmental restrictions.^1^ Therefore, in recent years, organic electrochemical synthesis appears to be a flexible and powerful method for assembling heterocyclic structures^2−5^ because it is more efficient and selective than the conventional synthetic methods, and more importantly the electrosynthesis correlates with nine of the 12 postulates of green chemistry (Figure 1). Agrochemicals, organic substances, medicines, and physiologically active natural items contain various heterocyclic structures,^6^ and crucially, at least one heterocyclic ring is present in greater than 70% of all pharmaceuticals and agricultural chemicals.^7^ Electricity-initiated organic transformations are inherently sustainable and environmentally favorable, requiring just mild working conditions.^8,9^

However, some concerns are obvious when using an electrochemical process in chemical transformations such as that comprehensive electrochemical equipment is required, which is typically costly to buy and operate. Additionally, a supporting electrolyte is frequently used to assist electron transport in solution, and because tetrahydrofuran, toluene, and other poorly conductive solvents are used, the choice of solvent can be challenging in electrosynthesis. The use of metal catalysts in electrochemical reactions under readily accessible undivided cells is relatively limited because the majority of metallic ions are readily reduced at the cathode to zerovalent metals, and when electrical and chemical reactions are carried out in split cells, costly ion exchange membranes are required for separating positive and negative electrodes.^10^ Several publications highlighting the electrochemical synthesis of heterocycles have recently been reported. Numerous reviews on electrochemical generation for heterocyclic systems may be found in the published studies. In 2019, Sbei and co-workers^11^ reviewed the synthesis of N-heterocycles, and the catalytic electrosynthesis of N, O-heterocycles has been reviewed in 2020 by the same group.^12^ In 2020, Ye and colleagues presented the electrochemical synthesis and functionalization of indole derivatives.^13^ Besides this, in 2021, we reviewed organic synthesis via Kolbe and related non-Kolbe electrolysis: An enabling electrostrategy.^14^ The paired electrolysis has been reviewed in 2021 by Ahmed and co-workers.^15^

**Figure 2:**
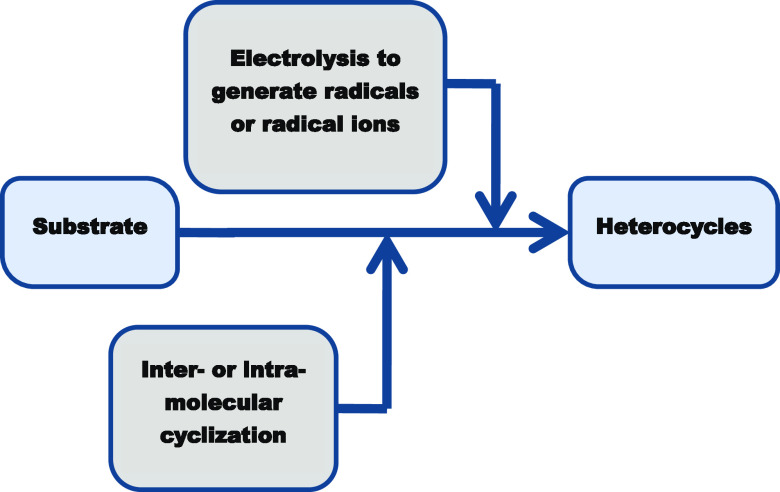
Synthesis of heterocycles aided by electrochemistry.^7^ Adapted with permission from ref (7). Copyright 2018, American Chemical Society.

Herein, in this study, we review the progress made in the electrochemical synthesis of N-, S-, O-heterocyclic compounds by intramolecular and intermolecular cyclization reactions over the last five years.

## 2 Electrosynthesis of S-, N-, O-Heterocycles

### 2.1 S-Heterocycles

The S-heterocycle family of organic compounds such as thiazole-2-imine **2** derivatives has led to a remarkable increase in their biological applications due to their intriguing therapeutic potential such as analgesic, kinase inhibition activities, anti-inflammatory, antibacterial,^16^ antifungal,^17^ and melanin reducing activity.^18^

In 2018, the electrochemical synthesis of thiazolidin-2-imines **2** from thiourea-tethered terminal alkenes **1** was reported by our group.^19^ The electrolysis was carried out in a flow-reactor using TEMPO as a redox catalyst. The 2,2,6,6-tetramethylpiperidine-*N*-oxyl radical (TEMPO) was used as mediator (catalyst) and react as nucleophilic reagents as shown in the proposal mechanism (Scheme 2).

**Scheme 1:**
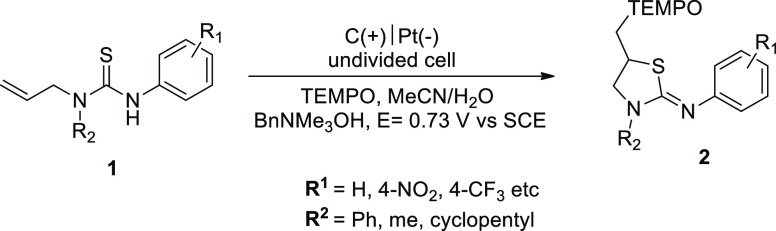
Electrochemical Synthesis of Thiazolidin-2-imines **2**

**Scheme 2:**
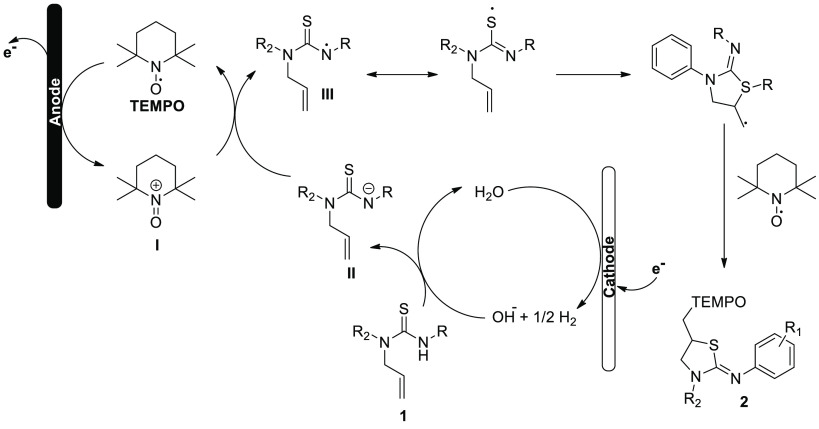
Proposed Mechanism for the Electrochemical Synthesis of Thiazolidin-2-imines **2**

According to the authors, the following mechanism is presented (Scheme 2). Cathodic reduction of water to OH^–^ and H_2_ is followed by anodic oxidation of TEMPO to produce oxo-ammonium ion **I**. Phenylurea **1** is deprotonated by the generated hydroxide ion, resulting in a nitrogen-centered anion **II**. An electron-deficient nitrogen-centered radical **III** is created by a succeeding SET between anion **II** and intermediate **I**, which replenishes the TEMPO radical molecule. The thiazolidin-2-imine **2** undergoes additional nitrogen radical tautomerization with the thiocarbonyl moiety to produce a sulfur radical. This radical develops cyclization to produce another radical at the terminal carbon, which interacts with the TEMPO radical molecule to produce the difunctionalized oxysulfurization product **2**.

**Scheme 3:**
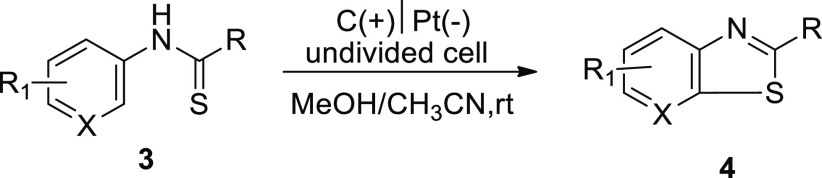
Electrochemical Synthesis of Benzothiazoles **4**

In 2018, another technique has been created for electrochemical dehydrogenative C–S bond synthesis in continuous flow without the use of a catalyst or a supporting electrolyte. In good to exceptional yields and with high current efficiency, a wide range of *N*-arylthioamides **3** have been converted to the corresponding benzothiazoles **4** (Scheme 3). Using carbon as the anode and a platinum plate as the cathode, the reaction was carried out in an undivided cell at a constant current. The authors demonstrated that this transition is accomplished solely by the use of a laboratory-grade solvent, and electricity without the use of degassing or a flux environment. This manuscript focuses on three benefits of electrochemistry in flow: (i) easy scaling up of the reaction without the use of a larger reactor; (ii) a supporting electrolyte-free reaction; and (iii) the critical and significant impact of having a good reaction solution which can be accomplished with the use of flow systems.^20^

**Scheme 4:**
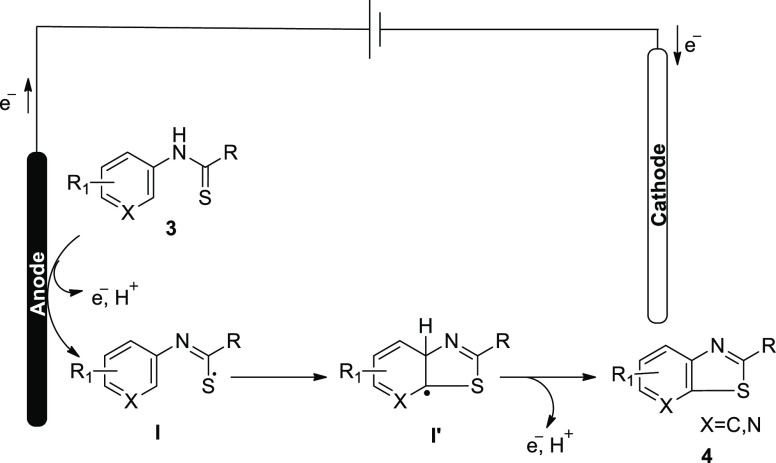
Proposed Mechanism for the Electrochemical Synthesis of Benzothiazoles **4**

The researchers speculated that the anodic oxidation was the first reaction in the chain of events of thioamides derivatives **3** to form the radical intermediates **I** and **I’**. This later after cyclization and deprotonation gives the final compound **4** (Scheme 4).

In 2019, intramolecular dehydrogenative C–S coupling was used to create the six-membered heterocyclic ring in 1,4-benzoxathiins and 1,4-benzothiazines.^21^ By executing the reactions in an acidic flow cell, oxidative desulfurization (a frequent side reaction for thioamides **5**) is avoided. The model substrate thioamide **5** was chosen to find the best reaction conditions. A flow electrolytic cell with a carbon filled polyvinylidene fluoride (C/PVDF)-anode and a Pt-cathode was used to conduct the electrolysis. Extensive testing demonstrated that when a continuous-flow electrolytic process was performed in a combination of solvents such as MeCN and TFA(9/1) in the availability of Sc(OTf)_3_, the required 1,4- benzoxathiin **6** could be extracted in 73% yield. The reaction used 2.4 F mol^–1^ (charge), which was significantly more than the theoretical 2F mol^–1^ used. Under the optimum circumstances, no desulfurized amide **7** was detected.

**Scheme 5:**
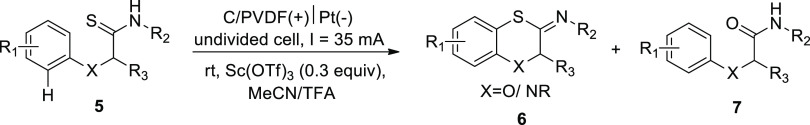
Electrosynthesis of 1,4-Benzoxathiin **6**

**Scheme 6:**
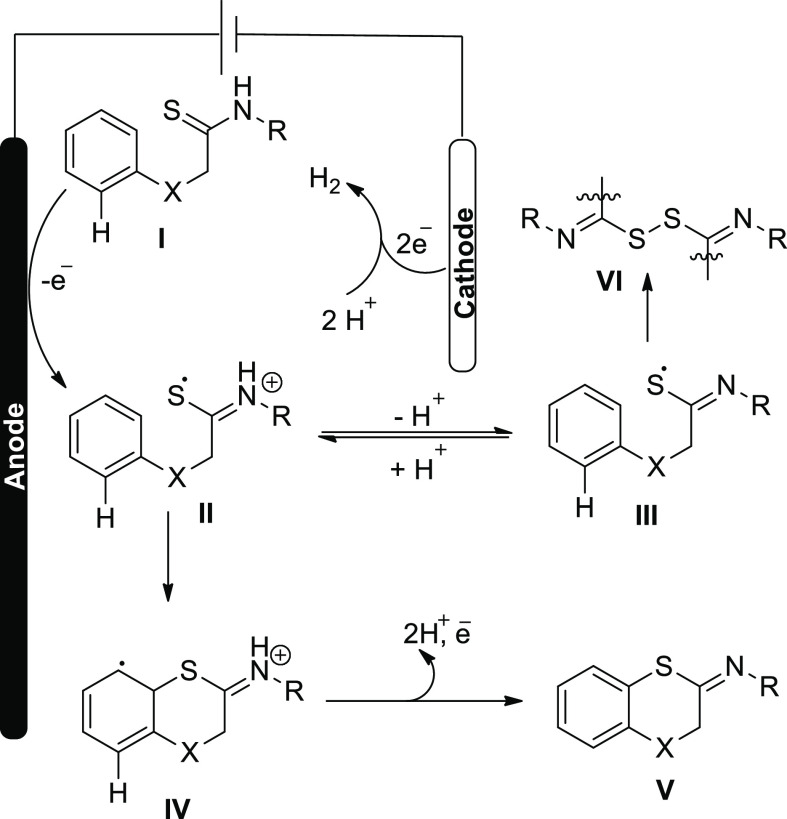
Proposed Mechanism for the Electrosynthesis of 1,4-Benzoxathiin **6**

The electrosynthesis mechanism was postulated (Scheme 6). The production of thioamidyl radical cation **II**, showing concordance with the neutral thioamidyl radical **III**, results from one-electron anodic oxidation of thioamide substrate **I**. The final product **V** is obtained by radical cyclization followed by oxidative rearomatization. It is anticipated that cyclization through the protonated radical **II** will be more successful than cyclization through neutral species **III** since the S-radical in intermediates **II** is more electrophilic than that in intermediates **III** and consequently more reactive toward the phenyl ring. TFA likely conducts ligand exchange with Sc(OTf)_3_ in the reaction mixture to produce the stronger acid (TfOH). This boosts the protonated form **II**’s level. The radical **III** can be dimerized and hydrolyzed to produce desulfurization product **6** with a gradual cyclization.

Similarly, in 2019, various functionalized 1,3-benzothiazines **8** were prepared by conducting the reactions in an acidic flow cell, and oxidative desulfurization, a frequent side reaction for thioamides, is avoided. The crucial intermediate is thought to be the thioamidyl radical cation. The electrolytic phenomenon was performed in a flow cell with Pt-cathode and carbon-anode loaded with polyvinylidene fluoride. A screening of the reaction parameters, including current (42 mA), flow rate (0.3 mL min^–1^), additives. and solvent (trifluoromethanesulfonic acid (TfOH, 0.06 M) in MeCN) revealed that is the best output. Addition of TfOH enhanced the conductivity of the reaction solution, so no supporting salt was required. The reaction of **5** produced the desired product **8** in good yield under these conditions.^22^

**Scheme 7:**
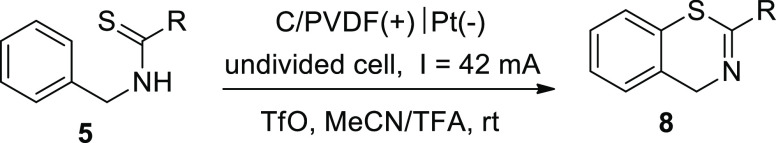
Electrosynthesis of 1,3-Benzothiazines **8**

**Scheme 8:**
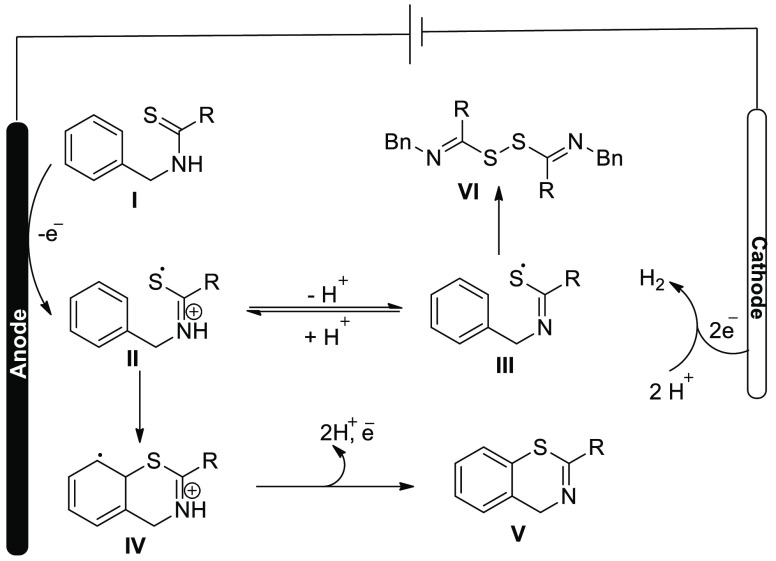
Proposed Mechanism for the Electrosynthesis of 1,3-Benzothiazines **8**

Based on the findings of this study, Scheme 8 offers an explanation that makes sense. At the anode, the thioamide **I** is oxidized by SET to produce radical cation **II**, which is then cyclized and oxidatively aromatized to produce the final heterocycle **V**. A proton can be deleted from the intermediate **II** to generate **III**, which is less susceptible to cyclization than **II**. Dimerization of the radical **III** can result in the radical **VI** which can then be hydrolyzed to produce desulfurized material. The addition of TfOH creates a more favorable balance on **II**, reducing desulfurization.

In 2019, it is stated that adding element sulfur **10** to N-tosyl hydrazones **9** results in the production of 1,2,3-thiadiazoles **11** using a metal- and oxidant-free electrochemical process. Tosylhydrazone **9**, which was produced from acetophenone, and sulfur **10** were coupled. Using TBAI (tetrabutylammonium iodide) (20 mol percent) as a catalyst and LiClO_4_ as an electrolyte in DMAc (dimethylacetamide) solvent at 120 °C for 6 h, a combination of **9** and **10** produced annulation product **11** in an 80% yield.^23^

**Scheme 9:**
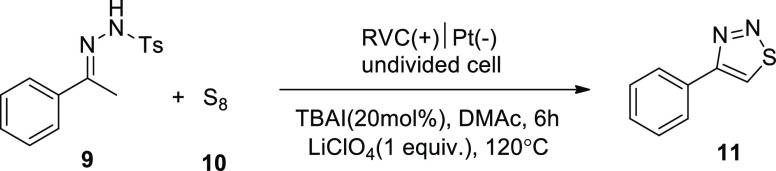
Electrosynthesis of 1,2,3-Thiadiazoles **11**

**Scheme 10:**
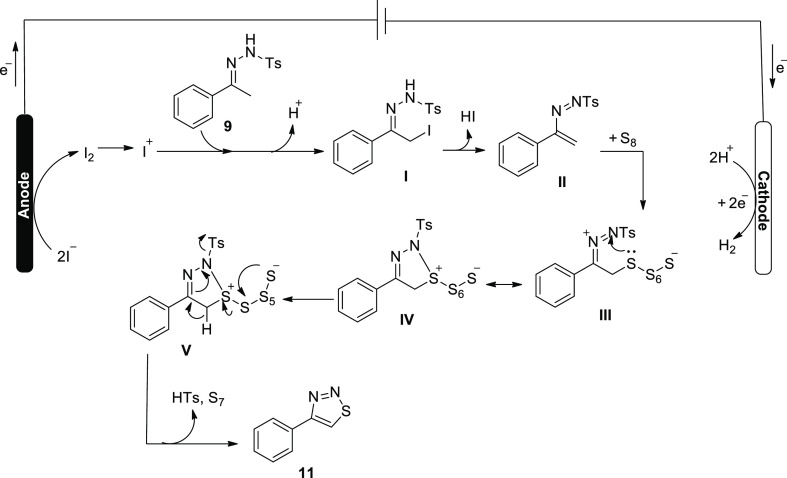
Proposed Mechanism for the Electrosynthesis of 1,2,3-Thiadiazoles **11**

A likely mechanism was put forth (Scheme 10). The electrochemical oxidation of 2I^–^ at the anode produced I_2_, which was efficiently converted into I^–^ and I^+^. Acetophenone tosylhydrazone **9** was converted into intermediate **I** by α-iodation, while azoalkene **II** was produced by HI elimination. The cycle of the reaction could be finished in these methods by further oxidizing the produced iodine anions. Zwitterion **III** was created by adding S_8_ to azoalkene **II**. This compound undergoes cyclization to yield intermediate **V**. Finally, the synthesis of the desired product **11** resulted from the removal of S_7_ and TsH. Reduction took place at the cathode to produce hydrogen, concluding the electrochemical cycle.

In 2021, under oxidant- and catalyst-free circumstances, a feasible and environmentally friendly electrochemical technique for the synthesis of C-3-sulfonated benzothiophenes **14** from 2-alkynylthio-anisoles **12** (5 mmol, 1.12 g) and sodium sulfinates **13** (2 equiv) was created. At a steady current, moderate to good yields of sulfonated benzothiophenes with significant and practical functional groups have been produced. An undivided cell with a graphite (C) rod serving as the anode and platinum (Pt) serving as the cathode was used to conduct the reaction. The electrolyte utilized was *n*Bu_4_BF_4_ (2.5 mmol), while the solvent used was CH_3_CN/H_2_O (2/1). In order to obtain product **14** in a 68% yield, the mixture was agitated at 30 °C and electrolyzed for 15 h at 8 mA (current) in an oil bath.^24^

**Scheme 11:**
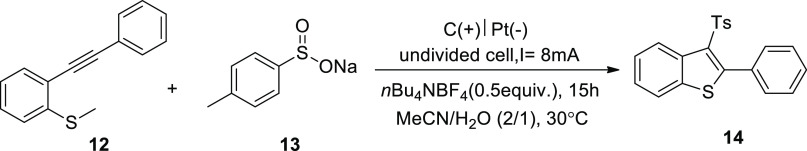
Electrosynthesis of C-3-Sulfonated Benzothiophenes **14**

**Scheme 12:**
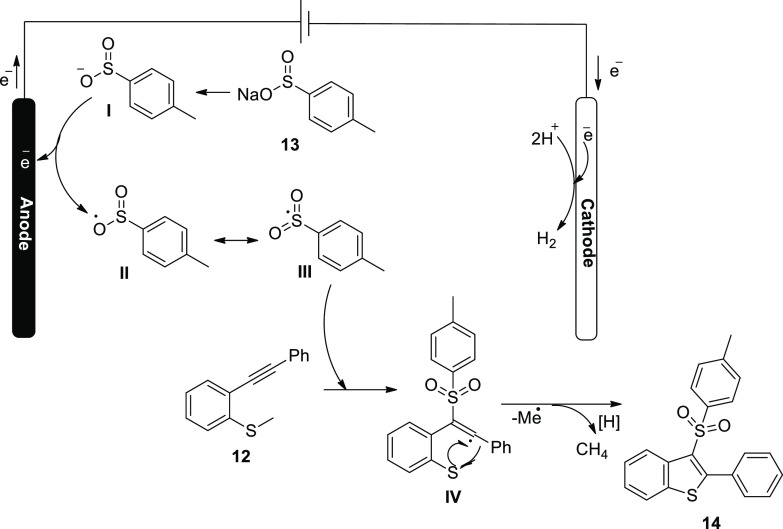
Proposed Mechanism for the Electrosynthesis of C-3-Sulfonated Benzothiophenes **14**

Scheme 12 shows a potential mechanistic pathway for the electrocatalytic sulfonylation process. Commencing with sodium *p*-tolylsulfinate **13**, which loses an electron at the positive electrode giving radical intermediate **II** or **III**, the reaction phase started. Resonance structures exist between the arylsulfonyl radicals **III** and **II**. The intermediate vinyl radical **IV** is created as a result of the intermolecular radical addition of sulfonyl radical **III** to the alkynyl molecule of **12**. The methylthio moiety then attacks the intermediate **IV** to release target product **14** and liberate methyl radical. Hydrogen from the reaction mixture then reacts with the methyl radical to produce methane.

### 2.2 N-Heterocycles

**Scheme 13:**
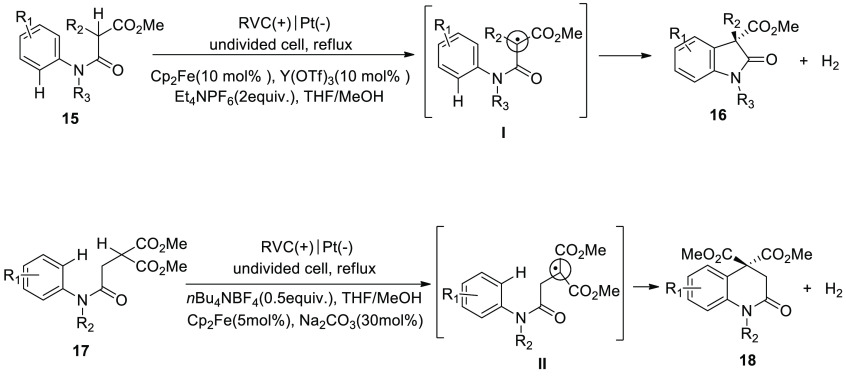
Electrosynthesis of Oxindole Cycle of 1,3-Dicarbonyl Compounds **16** and **18**

The importance of nitrogen-containing heterocyclic compounds for biomedicine^16^ has expanded synthetic methods available for their preparation. In 2018, Wu et al. reported the electrochemical reaction to achieve intramolecular C(sp^3^)–H/C(sp^2^)–H cross-coupling using Cp_2_Fe as a catalyst (Scheme 13). A family of oxindole cycle of 1,3-dicarbonyl compounds **16** and **18** was formed in good yield.^25^

**Scheme 14:**
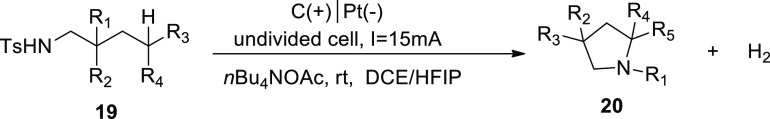
Electrosynthesis of Substituted Pyrrolidine **20**

Another method for the electrochemical formation of N-heterocycle was developed by Hu and co-workers in 2018 (Scheme 14). One of the methods for constructing saturated nitrogen-containing compounds is to cross-couple C(sp^3^)–H and N–H. In such operations, additional oxidizing agents or halogenated substances are typically needed. The authors reported an excellent work concerning the electrochemical synthesis of a five membered ring without the use of extra oxidants or toxic reagents. Starting from sulfonamide **19**, a large family of pyrrolidine **20** was obtained in fairly good yield. The electrolysis action was accomplished in an undivided cell with a platinum plate cathode and a carbon rod anode. Tetrabutylammonium acetate can generate an intermolecular hydrogen bond with amide and facilitate the cleavage of the N–H bond, in addition to being used as an electrolyte. In this method, the extra oxidants and N-halogenation step can be skipped. With good yields, benzylic and nonactivated primary, secondary, and tertiary C(sp^3^)–H amination can be produced.^26^

**Scheme 15:**
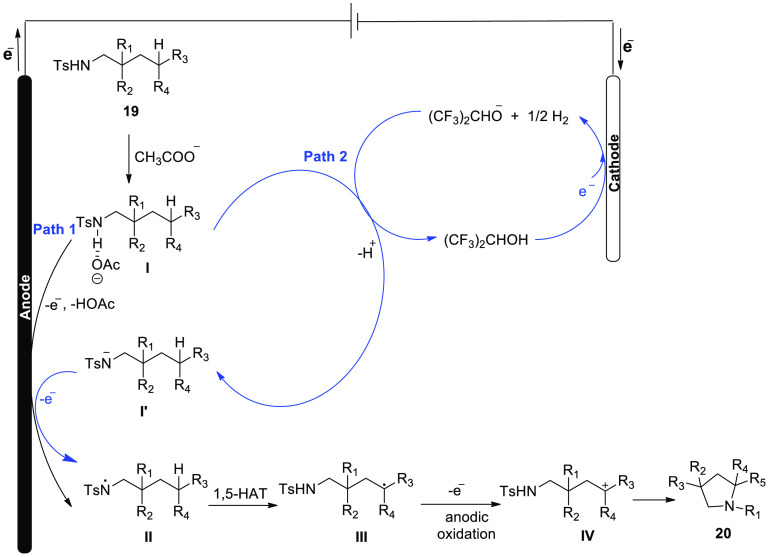
Proposed Mechanism for the Electrosynthesis of Substituted Pyrrolidine **20**

The reaction’s feasible mechanism is shown in Scheme 15. Bonding complexes **I** formed between sulfonamide **19** and acetate started the process. The production of the N-centered radical intermediate **II** was caused by a single electron oxidation on the anode (Path a). A C-centered radical **III** resulted from the 1,5-HAT (1,5-hydrogen Atom Transfer) of C–H bond by an aminyl radical. After that, the radical species was then oxidized to generate the carbon cation intermediate **IV**. Cyclization product **20** would be generated after the sulfonamide’s nucleophilic assault and proton eliminations. During the reaction phase, simultaneous cathodic reduction of produced protons would generate molecular hydrogen, avoiding the necessity of a stoichiometric exogenous oxidant. The produced alkoxide may deprotonate the substrate, resulting in the formation of the N-anion **I’**, which can then be quickly oxidized on the anode to produce the N-centered radical intermediate **II** (Path b).

**Scheme 16:**
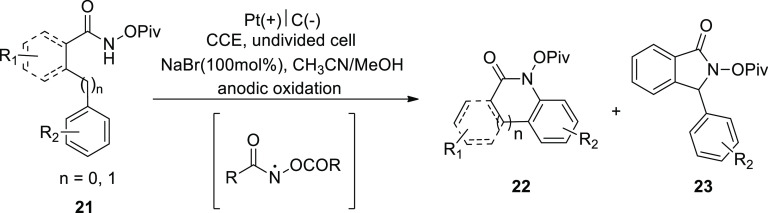
Electrosynthesis of Quinolinone **22** and Indolinone **23**

In 2018, for the first time, an inner-sphere electron-transfer process is used to generate N-acyloxy amidyl radicals electrochemically (Scheme 16). A single cell with a graphite (C) cathode and a platinum (Pt) anode was employed. The in situ produced amidyl radicals undergo intramolecular C(sp^2^/sp^3^)–H aminations using sodium bromide (catalyst and electrolyte), resulting in quinolinone **22** and indolinone **23** products with exceptional regio- and chemoselectivities.^27^

**Scheme 17:**
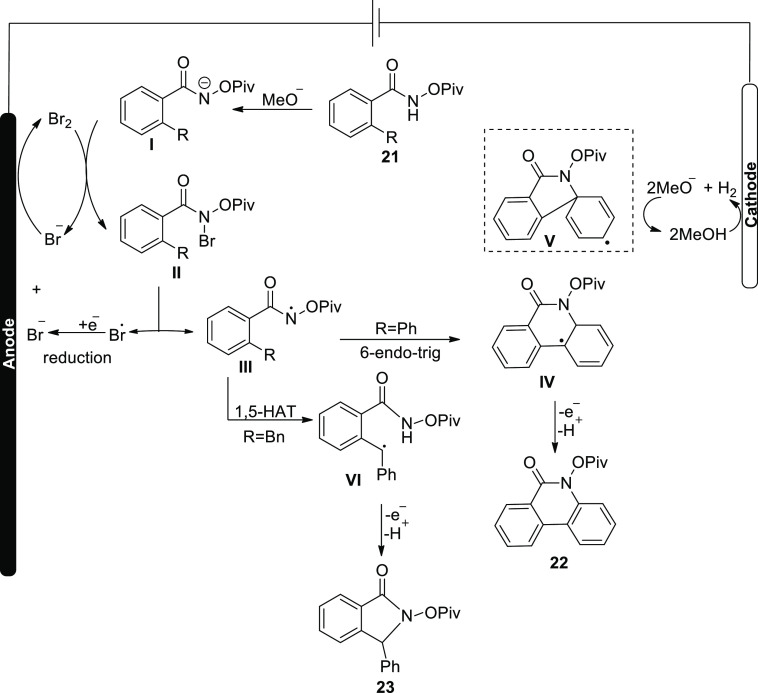
Proposed Mechanism for the Electrosynthesis of Quinolinone **22** and Indolinone **23**

An approach was hypothesized based on these results and related research (Scheme 17). Anodically produced Br_2_ is collected by the substrate **21** when a methoxide ion (electrogenerated base) (MeO−) is available, yielding intermediate **II**. Following nitrogen and bromine link breakage, the N-acyloxy amidyl **III** is produced, which follows cyclization (6-endo-trig) to yield intermediate **IV**. Bromine radical production was seen in the CV tests, supporting N-Br link cleavage. Steric hindrance of ^–^OPiv suppresses the potential undesirable intermediate. Finally, an aromatization changes intermediate **IV** into the sp^2^ C–H amination product **22**. In the production of benzylic radical **VI** in the sp^3^ C–H amination process, however, 1,5-hydrogen atom transfer has been hypothesized. The benzylic radical **VI** is further oxidized and then is intramolecularly cyclized to produce the product **23**.

**Scheme 18:**
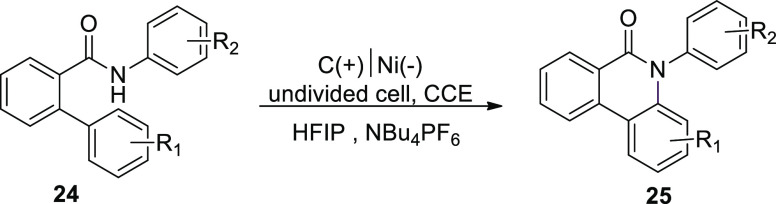
Electrosynthesis of *N*-Arylphenanthredin-6-one **25**

In 2018, another method for the electro-anodic oxidation was used to provide a new and long-lasting source of *N*-arylphenanthredin-6-one derivatives. *N*-(Phenyl) biphenyl-2-carboxamide**24** is converted into N,C coupled product **25** (Scheme 18) in an undivided Teflon cell having graphite (+) and nickel (−). 1,1,1,3,3,3-Hexafluoroisopropanol (HFIP) is taken as solvent. Using a moderator is not mandatory with this system due to its great electrical effectiveness. A straightforward and durable pathway to this class of compounds is provided by readily available and affordable starting ingredients. It is feasible to make a wide range of derivatives, and valuable functionalities that permit further reactions are recognized. This system can easily be scaled up or down.^28^

**Scheme 19:**
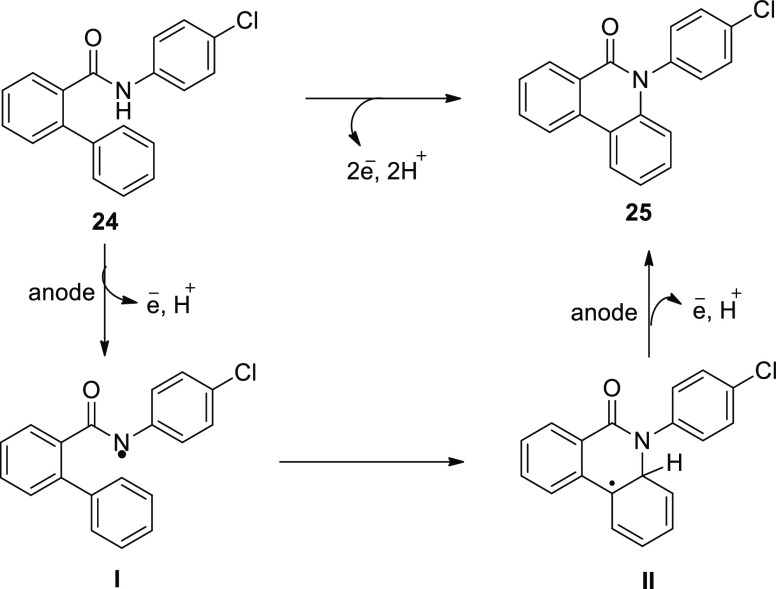
Proposed Mechanism for the Electrosynthesis of *N*-Arylphenanthredin-6-one **25**

Scheme 19 demonstrates the hypothesized method for the production of compound **25**. The cyclization is started by oxidation of the substrate at positive electrode (anode), which produces an amidyl radical **I**. An in situ produced HFIP anion could perform the deprotonation of the anilide. The N-aryl system can stabilize the amidyl radical. The amidyl radical forms an N,C connection with the second, unsubstituted phenyl moiety of the biphenyl scaffold in this case, resulting in a radical inside the lactam system. The product **25** is completed after a second oxidation phase, which is followed by a proton extrusion.

**Scheme 20:**
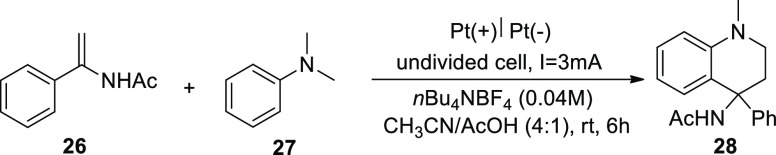
Electrosynthesis of *N*-(1-Methyl-4-phenyl-1,2,3,4-tetrahydro-quinolin-4-yl)acetamide **28**

In 2018, it was reported that a potent strategy (Scheme 20) for the quick assembly of six-membered heterocycles is the oxidative [4 + 2] annulation process. In this technique, tertiary anilines and alkenes undergo electrochemical oxidative [4 + 2] annulation to produce tetrahydroquinolines uniformly without the use of metals or external oxidants. To evaluate the reaction conditions, model substrates *N*,*N*-dimethylaniline **27** and *N*-(1-phenylvinyl)acetamide **26** were used. *N*-(1-Methyl-4-phenyl-1,2,3,4-tetrahydro-quinolin-4-yl)acetamide **28** may be produced in an undivided cell with a 72% yield using *n*Bu_4_NBF_4_ as the electrolyte and CH_3_CN/AcOH as cosolvents for 6 h.^29^

**Scheme 21:**
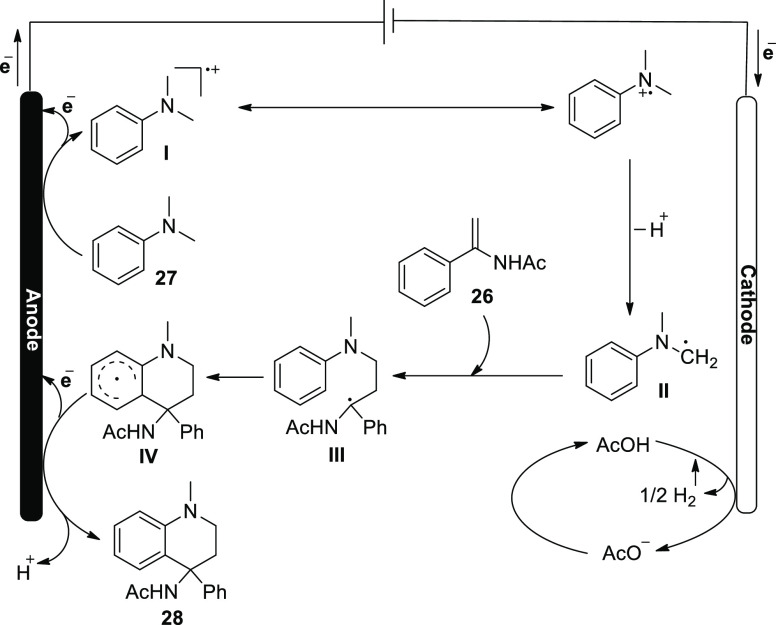
Proposed Mechanism for the Electrosynthesis of *N*-(1-Methyl-4-phenyl-1,2,3,4-tetrahydro-quinolin-4-yl)acetamide **28**

In Scheme 21, a viable mechanism for the reaction between **26** and **27** is presented. First, **27** is oxidized to produce a radical cationic that acetic acid can stabilize (anodic reaction). The tertiary-amino carbon radical is obtained after the radical cation resonates and then deprotonates. The radical **II** may then undergo a radical addition reaction with **26**. The subsequent anodic oxidation will produce the desired product **28** after the resulting radical species **III** participate in an intramolecular cyclization event. Hydrogen gas is created as a result of the simultaneous cathodic reduction of acetic acid.

In 2018, two related cobalt-catalyzed electrochemical methods have been used to produce substituted oxindoles via radical routes. It was shown that the electrochemical cobalt-catalyzed system was effective and environmentally benign because it did not require the use of stoichiometric oxidants to get good yields of the arylation **32** or alkylation **33** products at room temperature. A number of substituted oxindoles were easily made by electrochemically reacting *N*-arylacrylamides **29** with either potassium alkyltrifluoroborates **31** or arylhydrazines **30** under benign circumstances. RVC is used as the anode (+) and platinum is used as the cathode (−) to carry out the reaction. Without the use of oxidants, these two transformations have offered a revolutionary method for creating new radical oxidative couplings. In addition, a potential cocatalyzed radical reaction for the synthesis of compounds with all carbonic chiral core will occur when *N*-arylacrylamides and potassium alkyltrifluoroborates react. It is believed that these two novel techniques for obtaining substituted oxindoles will be beneficial for organic synthesis.^30^

**Scheme 22:**
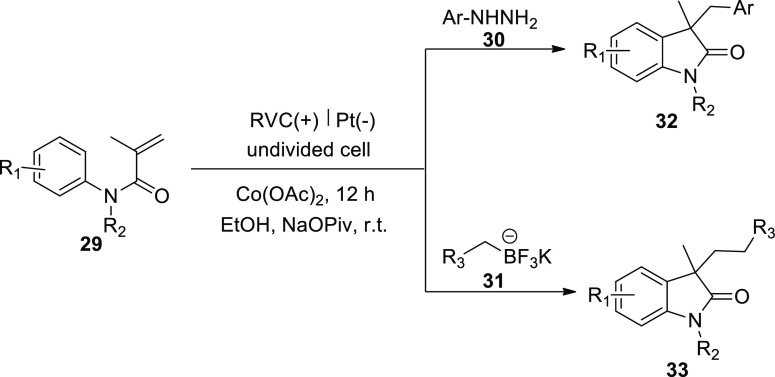
Electrosynthesis of the Arylation **32** and Alkylation **33** Products

**Scheme 23:**
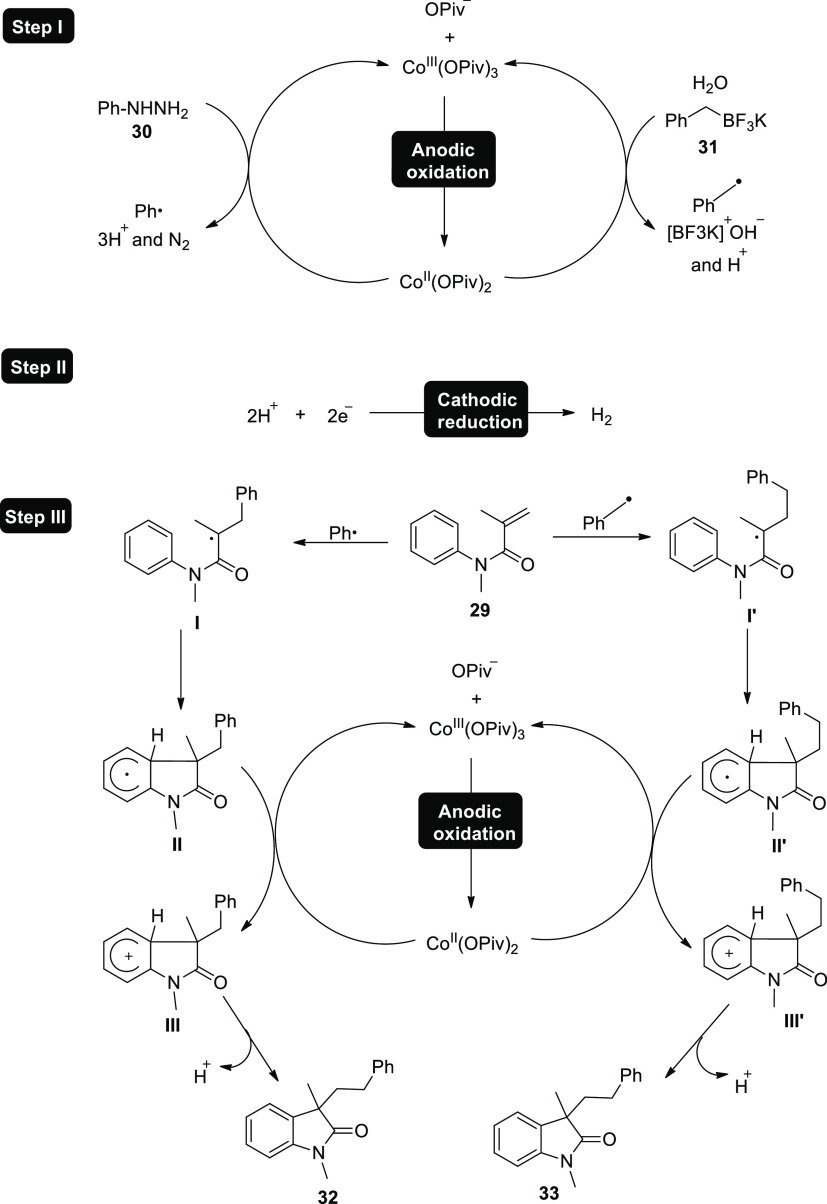
Proposed Mechanism for the Electrosynthesis of the Arylation **32** and Alkylation **33** Products

With the aid of Co salts, anodic oxidations of phenylhydrazine **30** or potassium benzyltrifluoroborate **31** produced phenyl or benzyl free radicals, which then served as the catalyst for the reaction (Scheme 23-Step I). Cathodic reduction occurs to give molecular hydrogen (H_2_) (Scheme 23-Step II). Ph· or Ph–CH_2_· directly assaulted **29** during the reaction to produce radical intermediate **I** or **I’**, and then intramolecular cyclization was used to produce radical **II** or **II’**. Following a single electron transfer from Co^III^ to Co^II^, the anode oxidized radical **II** or **II’** to produce the cationic intermediate **III** or **III’**. Finally, deprotonation moved intermediate **III** or **III’** to the intended product **32** or **33** respectively (Scheme 23-Step III).

**Scheme 24:**
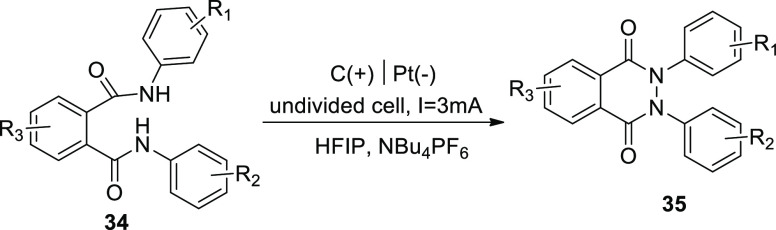
Electrosynthesis of Phthalazin-1,4-diones **35**

In 2018, electrochemical synthesis was used to create a new and sustainable access to phthalazin-1,4-diones **35** while avoiding highly poisonous and carcinogenic hydrazine chemicals. This technique was performed in an undivided cell possessing graphite-anode and platinum-cathode, while 1,1,1,3,3,3-hexafluoroisopropanol (HFIP) and NBu_4_PF_6_ were taken as solvents and phthaldianilide **34** as starting material (Scheme 24). This approach is a useful substitution for the conventional synthetic pathway since it uses readily available and low-cost starting components. A simple setup, the absence of metal catalysts and organic oxidizers, as well as scalable and long-lasting electrode materials, provide for easy and long-term access to this class of substrates. An anodic N–N bond is generated. This approach allows for a wide range of derivatives, valuable functionalities that enable future reactions are tolerated, and nonsymmetrical products are accessible.^31^

**Scheme 25:**
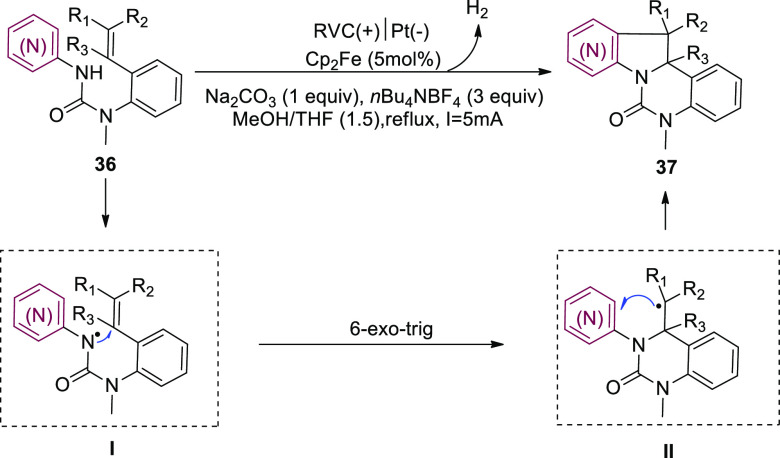
Electrosynthesis of Indolines and Azaindolines **37**

In 2018, the systematic and stereoselective formulation of indolines and azaindolines **37** using intramolecular dehydrogenative (3 + 2) annulation of heteroarylamines **36** with coupled substituted alkenes has been described with metal catalysts or oxidizing reagents (Scheme 25). The process uses a cascade radical cyclization to generate a carbon–carbon and carbon–nitrogen linkages in that order. Production of a 6-membered ring during the first step of the bicyclization ensures the efficient formation of the succeeding C–C bond, which is critical to the annulation’s success. From commercially accessible ingredients, the electrosynthetic method allows for the entire synthesis of (±)-hinckdentine A in 12 steps (LLS).^32^

**Scheme 26:**
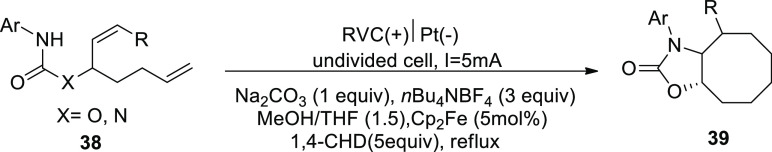
Electrochemical Synthesis of Functionalized 7-Membered Carbocycles **39**

In 2018, a 5-exo-trig or 7-endo-trig radical cyclization cascade for the electrochemical synthesis of seven-membered carbocycles **39** has been employed (Scheme 26). The cascade process’s first cyclization phase produces a 5-membered ring with radical center transposition and the leftover alkene. This trans-configuration causes region-selective 7-endo cyclization of the 6-heptenyl radical. This process was conducted in an undivided cell using mixture of methanol and tetrahydrofuran as solvent and *n*Bu_4_NBF_4_ as an electrolyte.^33^

To make axially chiral imidazopyridine-containing biaryls, the reactions go via a radical cyclization cascade.^34^ An innovative radical carbon–nitrogen linked cyclization (regiospecific [3 + 2] annulations) was employed to synthesize imidazo-fused N-heteroaromatics electrochemically.^35^ In 2018, the invention of a tetra-arylhydrazine as a catalyst for generating amidyl radicals and the discovery of unique reactivities of nitrogen- and carbon-centered radicals for carbon–nitrogen link construction have permitted this electrosynthesis. To this objective, an easily available carbamate **40** was used as a model substrate and an undivided cell with a reticulated vitreous carbon-anode and a platinum-cathode to test a number of different electrolysis conditions. When **40** was electrolyzed at a constant current in a mixed solvent of methyl cyanide and water under reflux in the vicinity of N_2_Ar_4_-catalyst, the best results were achieved. Despite their widespread availability, tetraarylhydrazines have never been used as a redox catalyst.^36,37^ The required imidazopyridine **41** was extracted in 89% yield under these circumstances.^38^

**Scheme 27:**
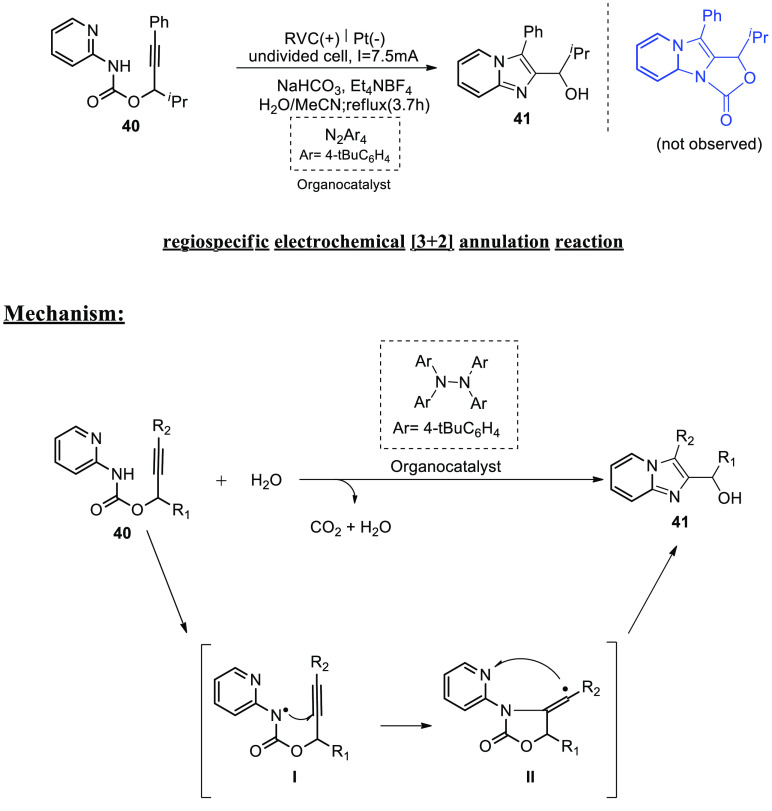
Proposed Mechanism for the Electrosynthesis of Fused Imidazo-pyridine **41**

**Scheme 28:**
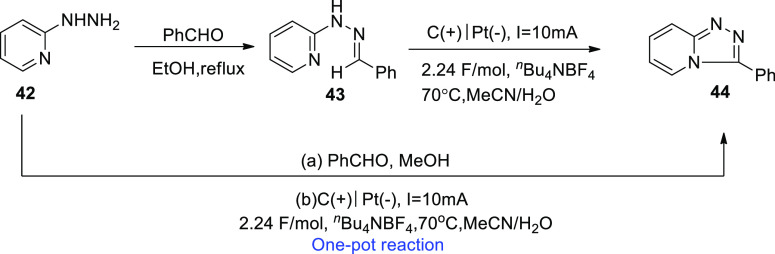
Electrosynthesis of 1,2,4-Trazolo[4,3-*a*]pyridines **44**

In 2018, an intramolecular dehydrogenative C–N cross-coupling method without reagents has been developed for moderate electrolysis (Scheme 28). Valuable 1,2,4-triazolo[4,3-*a*]pyridines **44** and its derivatives might be easily synthesized from easily accessible aldehydes and 2- hydrazinopyridines **42** in an atom- and step-economic one-pot procedure. This method, which is simple to use on a gram scale in the absence of oxidants or metals is suitable for a variety of functional groups. This innovative approach was used to synthesize one of the most popular medications, Xanax, as well as late-stage functionalization to produce chemical heterogeneity in biologically important lead compounds.^39^

**Scheme 29:**
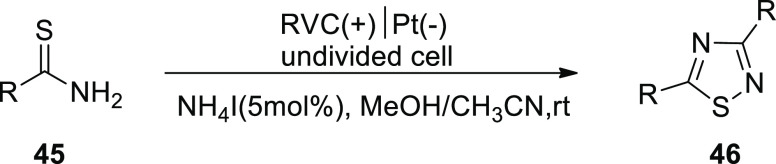
Electrosynthesis of 3,5-Disubstituted-1,2,4-thiadiazoles **46**

In 2018, the fabrication of 3,5-disubstituted-1,2,4-thiadiazoles **46** via an electrochemical approach using NH_4_I-mediated dimerization of thioamides **45** was described (Scheme 29). This electrosynthesis method uses ammoniumiodide as a catalyst and electrolyte, requiring no oxidizing chemicals and allowing the creation of a wide range of 1,2,4-thiadiazoles compounds. The process is an illustration of electrochemical S–N bond production.^36^

**Scheme 30:**
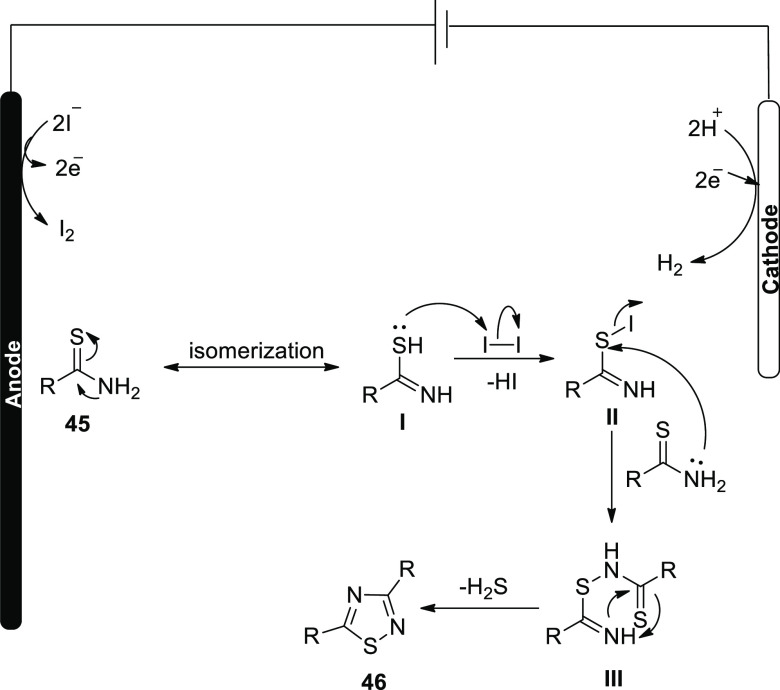
Proposed Mechanism for the Electrosynthesis of 3,5-Disubstituted-1,2,4-thiadiazole **46**

Scheme 30 serves as a demonstration of the suggested method for Scheme 29.

**Scheme 31:**
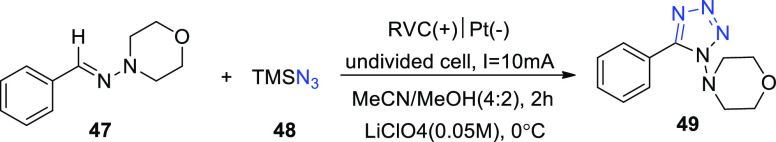
Electrosynthesis of 1,5-Disubstituted Tetrazole **49**

In 2018, the development of an ecologically acceptable electrochemical reaction for the production of useful functionalized tetrazoles has been made. No oxidants or metal catalysts were necessary for this simple reaction, and a variety of compounds were acceptable under the reasonable conditions. Importantly, this reaction is easily performed in a single pot or on a gram level. More uses of this technology are being developed.^37^ The electrochemical reaction was executed in a simple undivided cell under constant current circumstances. The anode is made of reticulated vitreous carbon, while the cathode is made of platinum plate. Tetrazole **49** was synthesized in 90% yield by electrolyzing hydrazone **47** and TMSN_3_**48** at 0 °C in a solvent mixture of methyl cyanide and methyl alcohol. The electrolyte used here was LiClO_4_ (Scheme 31).

**Scheme 32:**
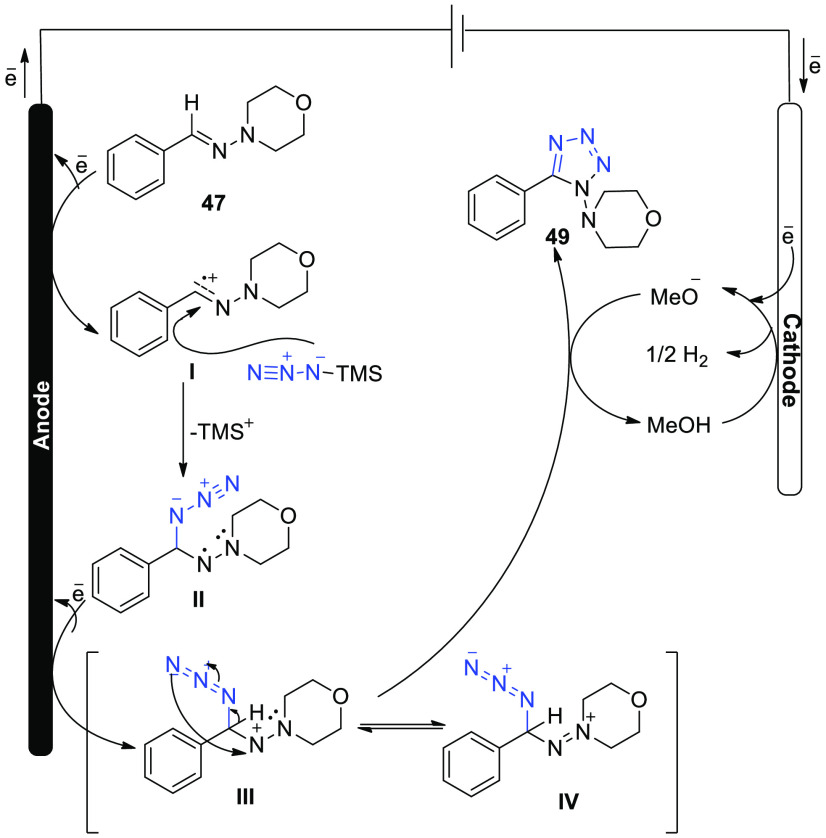
Proposed Mechanism for the Electrosynthesis of 1,5-Disubstituted Tetrazole **49**

The author proposes a feasible mechanism (Scheme 32). Carbocation intermediate **I** may be formed from **47** by anodic oxidation, which then combines with TMSN_3_ to formulate a C–N bond, yielding intermediate **II**. The anode may then oxidize the N-centered radical to produce intermediate **III**, which shows resonance with **IV**. Ultimately, following intramolecular cyclization and deprotonation (using methoxide ion formed by cathodic reduction of methanol) **III**/**IV** is converted into tetrazole product **49**.

**Scheme 33:**
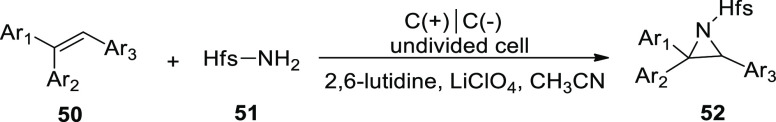
Electrosynthesis of Aziridine **52**

In 2018, an electrochemical technique was used to obtain the first immediate aziridination of alkenes, which might be extended to multisubstituted styrenes. As a nucleophilic nitrogen source, hexafluoroisopropanol sulfamate **51** was utilized. Mechanistic tests imply that this electrochemical mechanism occurs by forming two C–N bonds one at a time through interactions of sulfamate and cationic carbon species. To test the viability of such a technique, **50** underwent electrochemical methodology at a voltage of 5 V with LiClO_4_ as the electrolyte using graphite felt electrodes in the presence of sulfamate **51** in acetonitrile. With 2,6-lutidine as a base, the required aziridine **52** was achieved in 87% yield (Scheme 33).^40^

**Scheme 34:**
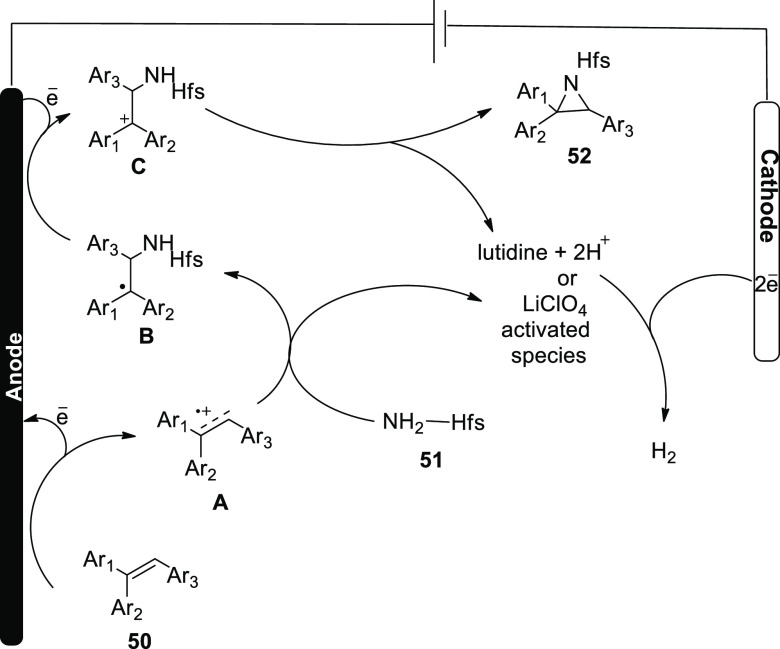
Proposed Mechanism for the Electrosynthesis of Aziridine **52**

It was suggested that a reaction pathway be developed (Scheme 34). The alkene is first anodized, resulting in the formation of carbocation radical **I**. The sulfamate nucleophile is added, and lutidine is used to deprotonate it, resulting in neutral radical species **II**, which is oxidized to carbocation C on the anode. Finally, the aziridine product **52** is obtained through ring closure. At the cathode, lutidine is regenerated by releasing hydrogen. In addition, LiClO_4_, activated 2,6-lutidine, or CH_3_CN can play a role in the cathode discharge process.

**Scheme 35:**
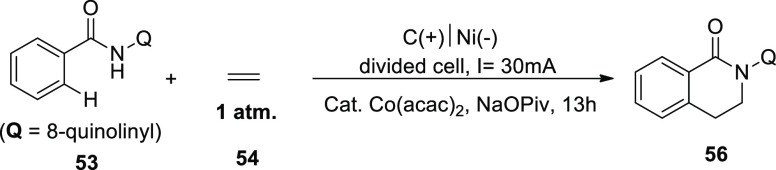
Electrosynthesis of 2-(Quinolin-2-yl)-3,4-dihydroisoquinolin-1(2*H*)-one **56**

In 2018, an electrochemical reaction approach was used to demonstrate the dehydrogenative C–H/N–H[4 + 2] annulation of amides **53** using ethylene **54** or ethyne **55**. However, there are just a few techniques that can be used to add ethylene or ethyne to fine compounds. Co(acac)_2_ was successfully used as a catalyst with ethylene **54** in the presence of sodium pivalate and undivided electrolytic conditions. At 4.0 mA constant current electrolysis, 89% of the isolated yield of the cyclization product **56** could be achieved after 4 h. This electrochemical process required the aminoquinoline directing group because no desirable product could be seen when the directing group was pyridine or pyridine-N-oxide. For a high reaction yield, the choice of a cobalt catalyst precursor was crucial. A 5 mmol scale reaction with a 30 mA constant current in a larger divided cell was attempted (Scheme 35). Both the anode and the cathode electrodes were made of carbon fabric. Fortunately, 0.90 g (66%) of **56** could be extracted after 13 h of electrolysis.^41^

**Scheme 36:**
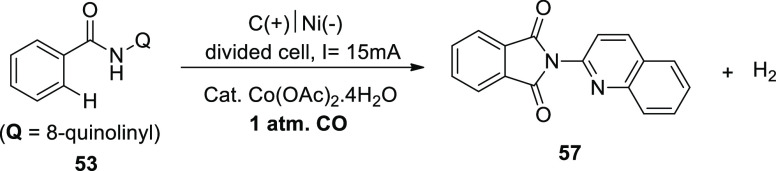
Electrosynthesis of 2-(Quinolin-2-yl)isoindoline-1,3-dione **57**

One of the easiest ways to make carbonyl compounds is oxidative carbonylation with carbon monoxide (Scheme 36), which is catalyzed by transition metals. In 2018, it was possible to carry out the intramolecular C–H/N–H carbonyltion via anodic oxidation by using 4-methyl-*N*-(quinoline-8-yl)benzamide **53** as the sample material. At 15 mA constant current electrolysis, a **57** isolated yield of 85% could be produced. Co(OAc)_2_·4H_2_O catalyst revealed the highest performance.^42^

**Scheme 37:**
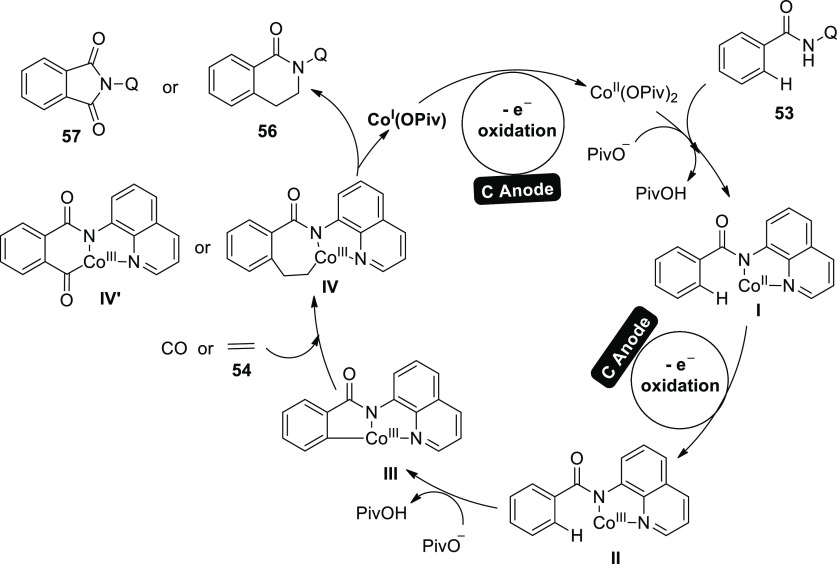
Proposed Mechanism for the Electrosynthesis of 2-(Quinolin-2-yl)-3,4-dihydroisoquinolin-1(2*H*)-one **56** and 2-(Quinolin-2-yl)isoindoline-1,3-dione **57**

A potential mechanism (Scheme 37) for Scheme 35 and 36 is put forth in light of the results reported above. With the aid of NaO^–^ Piv·H_2_O, Co(II) complex **I** can be produced as a bidentate nitrogen coordinated Co(II) complex, which initially coordinates with **53**. Next, the anode directly oxidizes complex **I** to produce Co(III) complex **II**. Complex **II** undergoes intramolecular C–H activation to produce cyclic Co(III) complex **III** with the aid of NaOPiv·H_2_O. The end products **56** and **57** are created by ethylene **54** or CO insertion and reductive removal of the Co(III) species, respectively. In order to replenish the Co(II) catalyst, the Co(I) species produced following reductive exclusion are oxidized by the C-anode. Since a significant amount of hydrogen gas can be found in the reaction system by GC after the reaction is stopped, proton reduction is most likely the accompanying cathodic reaction.

**Scheme 38:**
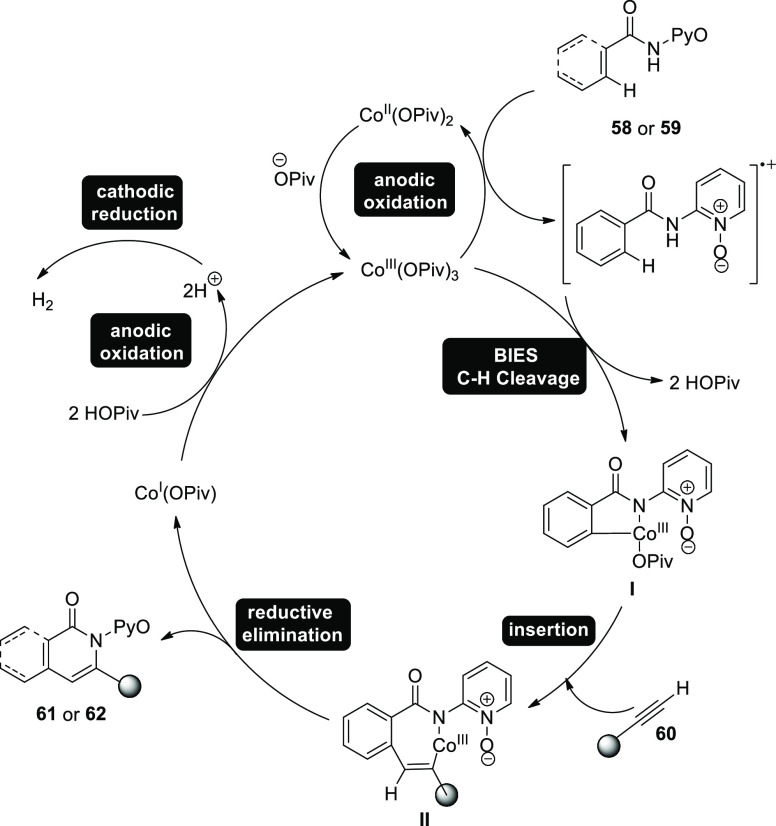
Proposed Mechanism for the Electrosynthesis of Isoquinolones **61** and Pyridones **62**

In 2018, competition experiments, kinetic-isotope-effect (KIE) measurements, and CV investigations were done to shed light on the process of C–H/N–H annulation. These mechanistic discoveries led to the development of an illustration (Scheme 38), which included the production of the intermediate **I** and the addition of the alkyne **60** to create the essential intermediate **II**. By using reductive elimination, the desired product **61** or **62** is ultimately fabricated.^43^

**Scheme 39:**
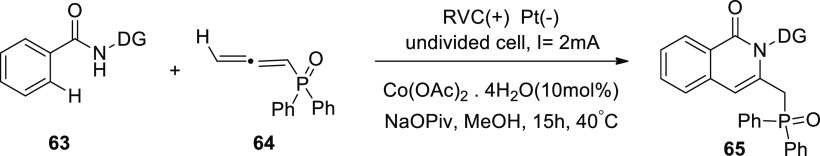
Electrosynthesis of C–H Annulation Product **65**

In 2018, allenes were used to activate C–H electrochemically coupled to versatile cobalt catalysis. Thus, under exceptionally mild conditions, allene **64** annulations were accomplished with respect to C–H/N–H functionalizations with good degrees of chemo-, site-, and regioselectivity. Substrate **63** is converted into product **65**. While it was discovered that an RVC anode was advantageous, several cobalt-salts might be used as the precatalyst. The electrochemical C–H activation was effective with a range of solvents, including polar protic alcohols, THF and CH_2_Cl_2_. Therefore, MeOH-solvent and NaOPiv-additive provided the best reaction conditions (Scheme 39).^44^

**Scheme 40:**
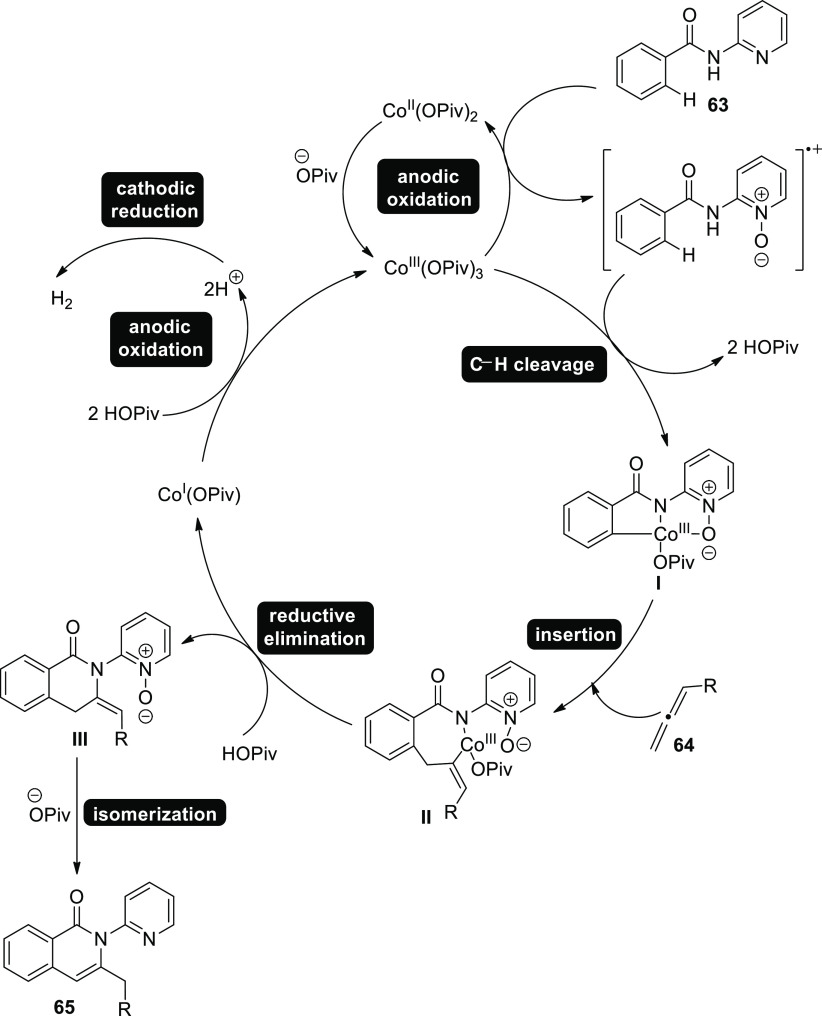
Proposed Mechanism for the Electrosynthesis of C–H Annulation Product **65**

Based on our experimental and theoretical mechanistic analyses, the suggested mechanism for Scheme 39 is mentioned in Scheme 40. It prepares the way for a successful BIES–C–H scission with carboxylate (COO^–^) support. The exomethylene isoquinolone **III** is then produced by the addition of allene **64** and reductive elimination, which isomerized to yield product **65**. After that, the crucial anodic oxidation regenerates the active cobalt catalyst, with the only waste being H_2_.

**Scheme 41:**
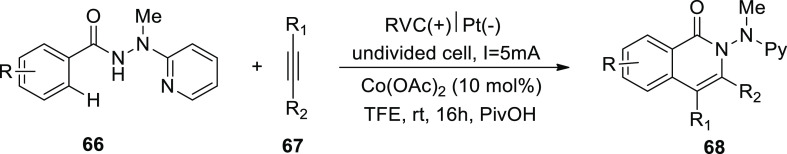
Electrosynthesis of Substituted Isoquinolone **68**

In 2018, flexible cobalt(Co)-catalyzed annulation and activation of internal alkynes **67** and **66** to achieve substituted isoquinolone product **68** were displayed (Scheme 41). Earth-abundant cobalt catalysts were used in an undivided cell setup with extremely modest reaction parameters at room temperature to demonstrate the viability of the electro-oxidative C–H activation array. By preventing the usage of metallic oxidizing agents, electrochemical cobalt catalysis produces only H_2_ as a byproduct.^45^

**Scheme 42:**
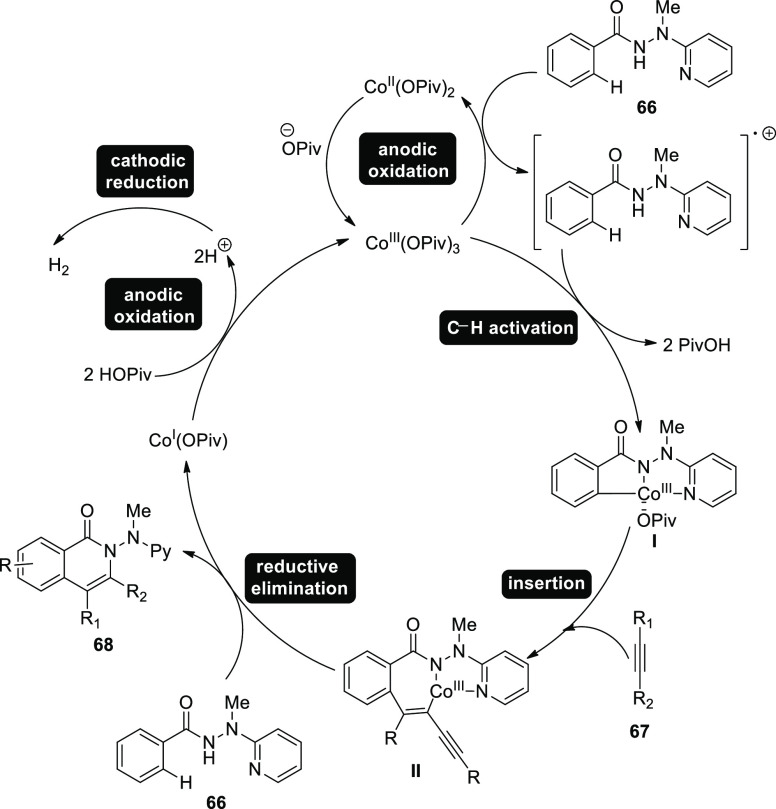
Proposed Mechanism for the Electrosynthesis of Isoquinolone **68**

A reasonable mechanism for Scheme 41 has been shown in Scheme 42 based on the fundamental investigations. First, anodic oxidation is used to create the catalytically capable cobalt(III) salt. Next, simple C–H cobaltation with carboxylate assistance produces cobalt(III) species **I**. Following migratory insertion, cobalt(III) complex **II** is produced. The simultaneous release of isoquinolone **68** and cobalt(I) intermediate followed by reductive elimination. Anodic oxidation was used to regenerate cobalt(III) carboxylate, which is capable of catalysis. Overall, the cobaltaelectrocatalysis avoids using stoichiometric amounts of expensive and harmful oxidizing substances, generating hydrogen.

In 2018, it has been found that readily available ethyl naphthalen-1-ylcarbamate **69** electrooxidatively annulates alkyne **67**. Rhodium(III) catalysts that are frequently employed failed to produce the product **70.** DMF and t-AmOH were among a group of typical solvents that produced promising results, but addition of potassium acetate in a solvent mixture (t-AmOH–H_2_O) turned out to be the most effective.^46^

**Scheme 43:**
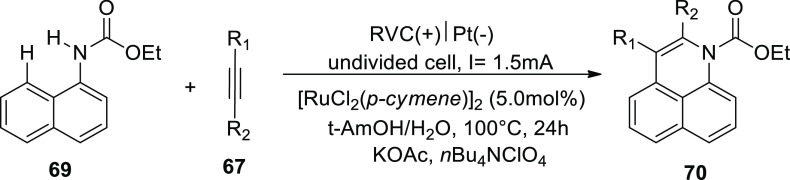
Electrosynthesis of Rhodium(III) Catalyzed Annulated Product **70**

**Scheme 44:**
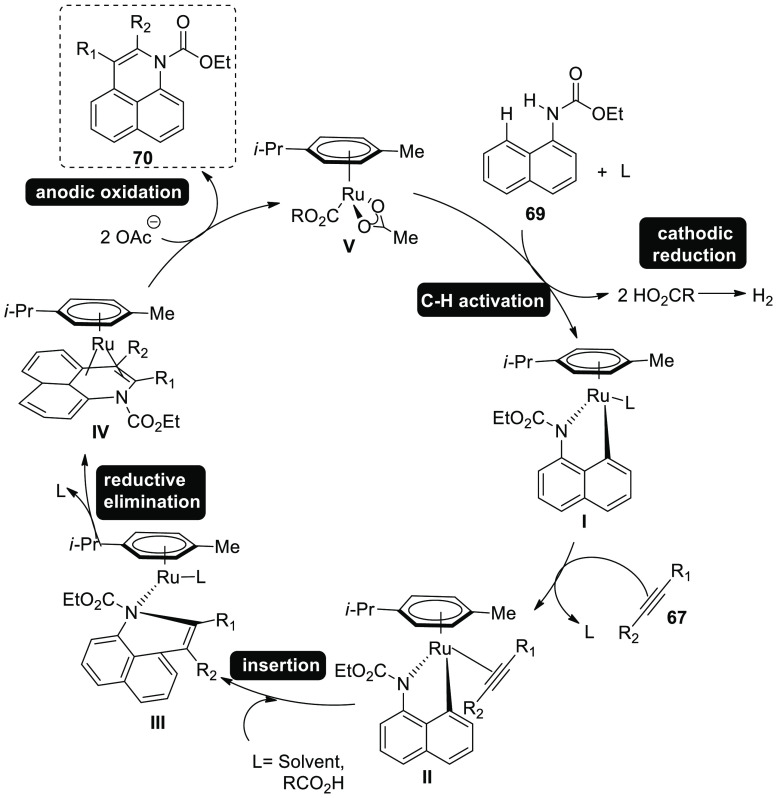
Proposed Mechanism for the Electrosynthesis of Rhodium(III) Catalyzed Annulated Product **70**

Mechanistic research suggests that an easy organometallic C–H activation will start a feasible catalytic cycle (Scheme 44). Thus, ruthena(II)cycle **I** and two equivalents of carboxylic acid are produced. The seven-membered ruthena(II) cycle **III** is then provided by alkyne inclusion, and is quickly transformed into ruthenium(0) sandwich complex **IV** on reductive-elimination. Anodic-oxidation is ultimately responsible for the crucial reoxidation of the resulting ruthenium(0) complex **IV**, whereas cathodic reduction produces just molecular hydrogen as a stoichiometric by product.

**Scheme 45:**
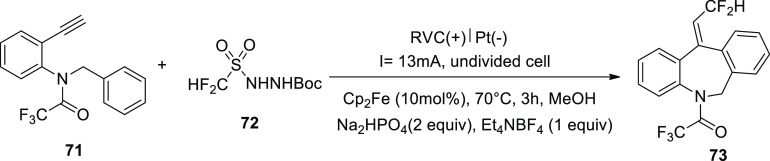
Electrosynthesis of Fluorinated Dibenzazepine **73**

In 2018, by finding a new difluoromethylation reagent, CF_2_HSO_2_NHNHBoc **72**, an entirely unknown scheme has been created to obtain fluorinated dibenzazepines **73** (Scheme 45). To produce **73**, the CF_2_H radical produced in the presence of ferrocene takes part in a novel alkyne **71** position and a difficult homolytic aromatic substitution step that forms a 7-membered ring. Thus, the stereoselective synthesis of fluorinated dibenzazepine **73** (yield = 70%) was achieved by electrolyzing amide **71** carrying a terminal alkynyl group as the radical acceptor. This formulation occurs in the presence of methanol at 70 °C utilizing ferrocene (Cp^2^Fe) as the mediator.^47^

**Scheme 46:**
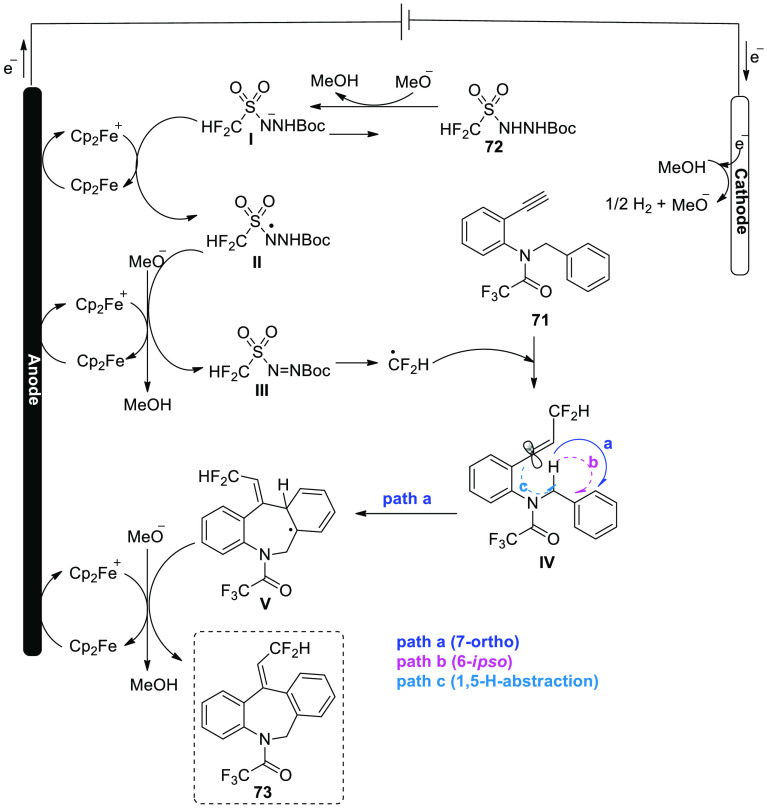
Proposed Mechanism for the Electrosynthesis of Fluorinated Dibenzazepine **73**

A workable explanation for Scheme 45 is offered (Scheme 46). Oxidation of Cp_2_Fe to Cp_2_Fe^+^ is the first step in the electrolytic reaction. Cathodic reduction of methanol (CH_3_OH) in the interim produces H_2_ and MeO^–^. As a result, diazene **III** is produced by Cp_2_Fe^+^ oxidizing **I**, the conjugate base of **72**, most likely with the help of N-radical **II**. Converted current efficiency may result from Cp_2_Fe^+^ being reduced to Cp_2_Fe at the cathode in an undivided cell. After **III** is broken down, the CF_2_H radical is produced, and it interacts with **71** to produce vinyl radical **IV**. According to computational simulations (Scheme 46), the 7-ortho cyclization (route-a) was kinetically preferred for the current process over the 6-ipso cyclization (route-b) or 1,5-H abstraction (route-c). In order to create the radical intermediate **V**, the C-radical **IV** undergoes a regio- and stereoselective path-a. Finally, the dibenzazepine product **73** results from rearomatization of **V** by electron (e^–^) and proton (H^+^) removal.

**Scheme 47:**
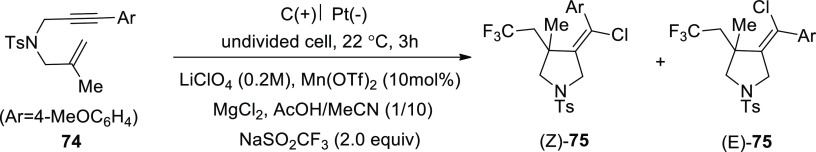
Electrosynthesis of Chlorotrifluoromethylated Pyrrolidines **75**

In 2018 and 2020, it has been reported on an electrocatalytic method for making chlorotrifluoromethylated pyrrolidine derivatives. Anodically linked electrolysis, in which a pair of reactive radical species are simultaneously produced anodically and then undergo a convergent and beneficial reaction, allows for this process. A redox-active Mn catalyst regulates the addition of these intermediates to the alkene. The eneyne cyclization products can be produced with great stereoselectivity with respect to the alkene geometry by using 2,2′- bipyridine as the ligand. Interestingly, under barely altered reaction conditions, difunctionalization of 1,6-enyne substrates **74** produced chlorotrifluoromethylated pyrrolidines **75**. In this procedure, LiClO_4_ was utilized as an electrolyte and mixture of acetic acid and methyl cyanide as solvent in an undivided cell. The reaction is performed at 22 °C for 3 h. In this case, the 2,2′-bipyridine (bpy) bidentate ligand considerably improved the stereochemistry of products **75** (Scheme 47).^48,49^

**Scheme 48:**
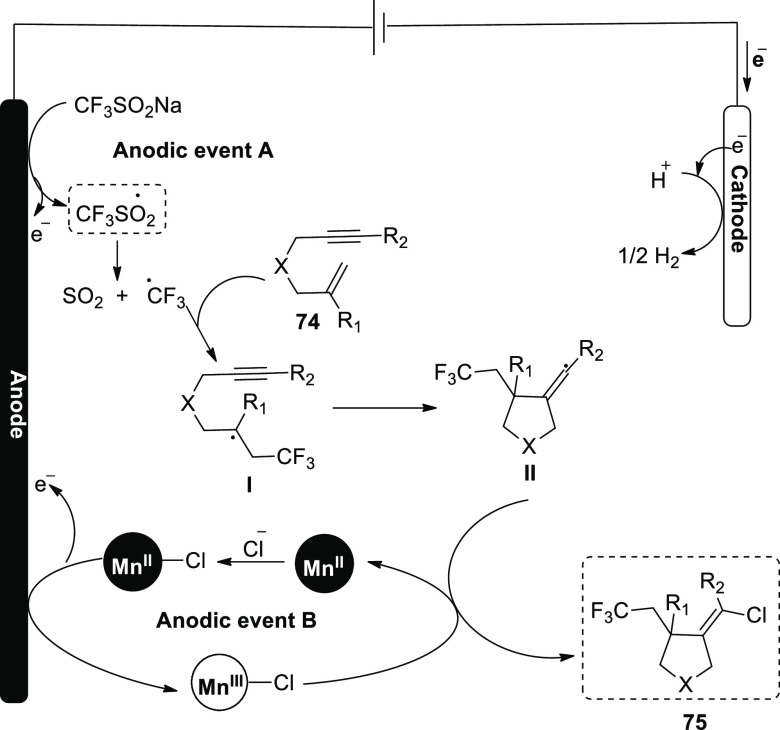
Proposed Mechanism for the Electrosynthesis of Chlorotrifluoromethylated Pyrrolidines **75**

In order to catalyze the electrochemical ene-yne cyclization, a cycle was devised (Scheme 48). Anodically linked electrolysis allows the catalyst and functional group donors to permit the anodic event **A** and **B** occurrence. A sp^3^ carbon-centered radical **I** is created when the transient and highly reactive CF_3_ radical is added to the trisubstituted alkene **74**. This intermediate is subsequently intramolecularly added to the alkyne to create an intermediate with an alkenyl radical **II**. This very reactive carbon-centered radical is used in the vicinity of an open-shell metal complex ([Mn^III^]-Cl), and is transformed into an alkenylchloride **75** (radical atom transfer). In this procedure, catalyst undergoes single-electron oxidation on the electrode and transitions back to the Mn^II^ oxidation state.

**Scheme 49:**
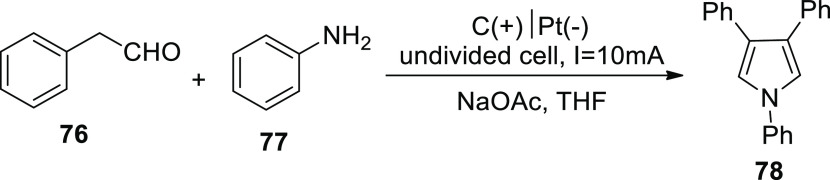
Electrosynthesis of 1,3,4-triphenyl Pyrrole **78**

Gao and associates published a fantastic technique for producing pyrrole derivatives in 2019. Pyrroles **78** was formed in an undivided cell using carbon plate-anode and platinum-cathode. Electrolyzing simple and readily available arylacetaldehydes **76** and primary amines **77** at constant current of 10 mA provided a comprehensive series of pyrrole derivatives in excellent ratios (Scheme 49). Furthermore, in modest environments, this reaction might operate with strong functional group resistance and reproducibility.^50^

**Scheme 50:**
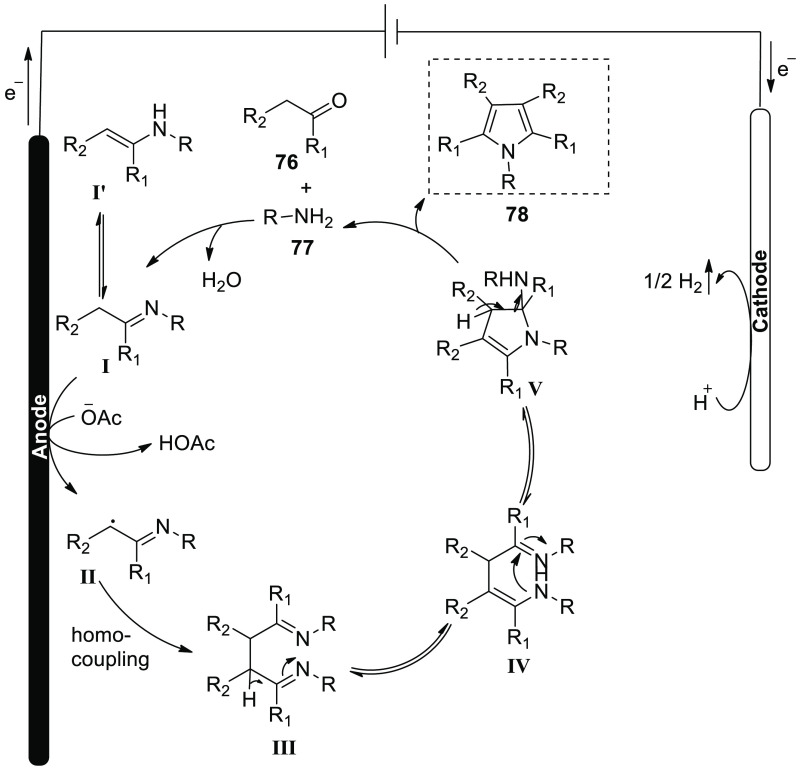
Proposed Mechanism for the Electrosynthesis of 1,3,4-Triphenyl Pyrrole **78**

Scheme 50 shows the detailed mechanism of Scheme 49. To begin, imine **I** is made up of arylacetaldehyde **76** and primary amine **77**, which can isomerize to enamine. At the positive electrode (anode), SET-oxidation of imine resulted in the formation of benzyl radical **II**. Finally, benzyl radicals self-coupled to create complex **III**, which, before intramolecular nucleophilic attack and cyclization to create the requisite chemical **78**, would isomerize to enamine.

**Scheme 51:**
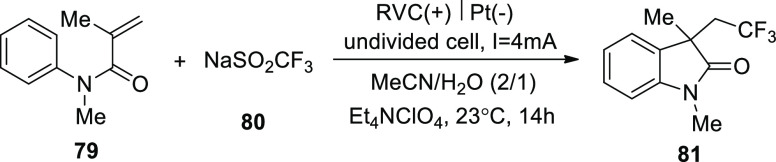
Electrosynthesis of 1,3-Dimethyl-3-(2,2,2-trifluoroethyl)indolin-2-one **81**

In 2019, under external oxidant-free circumstances, an electrolytic procedure was accomplished without the use of a catalyst. The method (Scheme 51) uses a moderate, direct electrolysis of sodium sulfinates **45** in an undivided cell with the use of methylcyanide and water (solvent mixture) and Et_4_NClO_4_ salt as an electrolyte. Under constant current conditions, *N*-arylacrylamide**79** was electrochemically trifluoromethylated and cycled with CF_3_SO_2_Na **80**. This concept can be applied to a wide range of functional groups. In a yield of 74%, the intended product 1,3-dimethyl-3-(2,2,2-trifluoroethyl)indolin-2-one **81** was attained.^51^

**Scheme 52:**
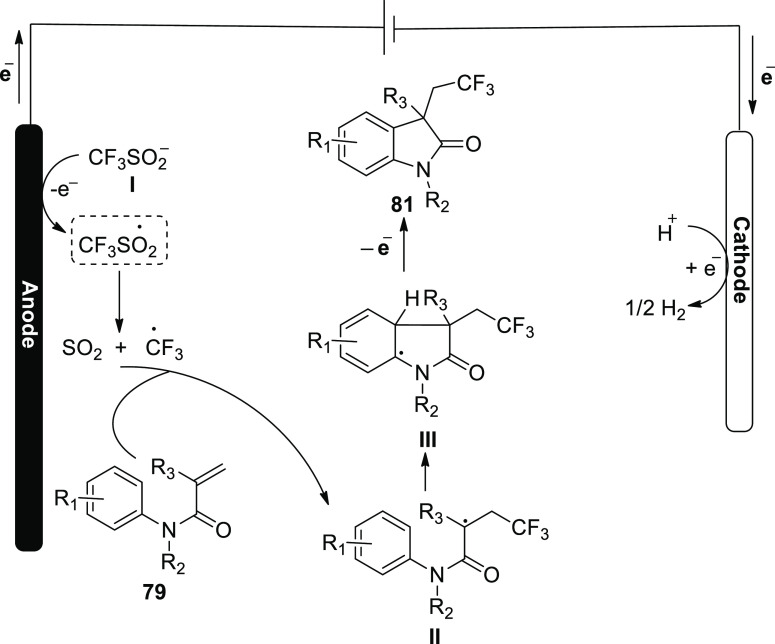
Proposed Mechanism for the Electrosynthesis of 1,3-Dimethyl-3-(2,2,2-trifluoroethyl)indolin-2-one **81**

A tenable mechanistic hypothesis was put forth for Scheme 51 in Scheme 52. Corresponding radical is created, which converts sulfinate anion **I** to CF_3_ radical in a desulfurative reaction. In the region of the anode, the CF_3_ radical attacks the alkene **79** to produce intermediate **II**, and this is transformed into intermediate **III**. Under anodic oxidation circumstances, more aromatization results in the equivalent product **81**. On the cathode, hydrogen cations are simultaneously reduced, yielding molecular hydrogen.

**Scheme 53:**
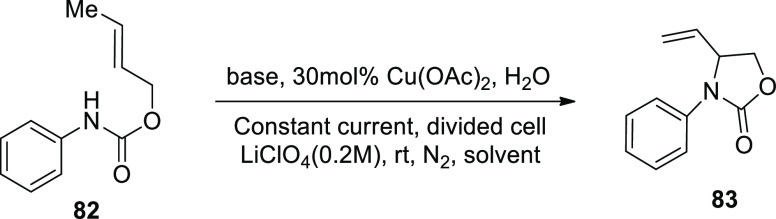
Electrosynthesis of Alkene Substituted Oxazolone **83**

In 2019, Hu and companions published a new technique for making N–O heterocycles. An aza-Wacker cyclization process was devised by copper-catalyzed electrosynthesis. This tandem approach allows for substrate transformations that result in R·-intermediates, considerably expanding the action range. Under mild circumstances, alkene substituted oxazolone **83** was fabricated. They began with electrochemical oxidative amination of crotyl *N*-phenylcarbamat **82** in the presence of a copper catalyst. They discovered that the desired product **83** could be obtained (Scheme 53) at room temperature in a divided cell with methanol (solvent), carbon fiber (electrode), LiClO_4_ (electrolyte), Cu(OAc)_2_ (catalyst), NaOAc (base), and 3 mA (constant current).^52^

**Scheme 54:**
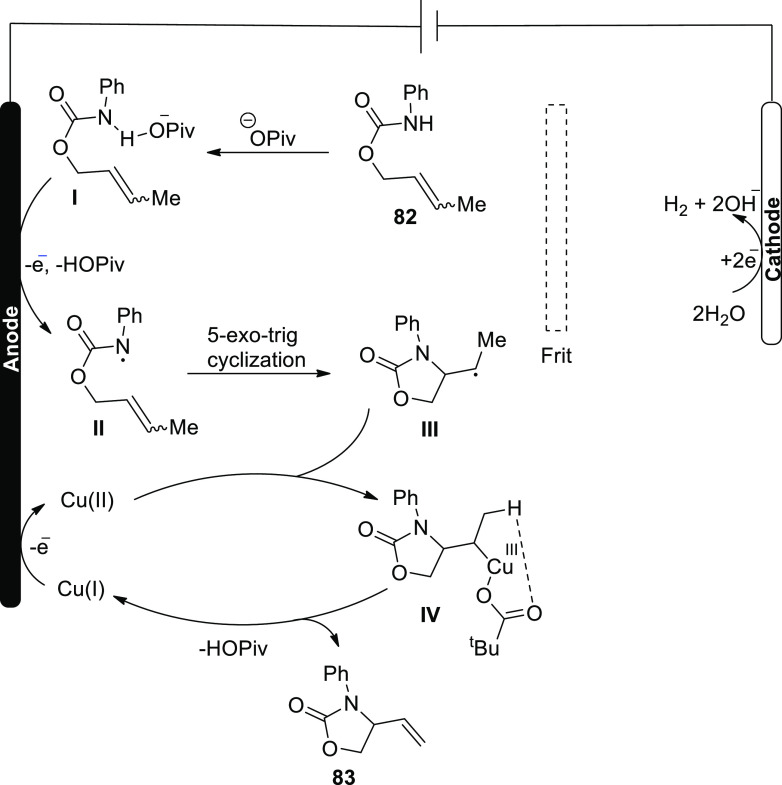
Proposed Mechanism for the Electrosynthesis of Alkene Substituted Oxazolone **83**

Based on the aforesaid findings, the electrochemical formal aza-Wacker cyclization mechanism was developed (Scheme 54). The substrate **82** first forms an adduct with the base, **I**, which is then oxidized at anode to form an amidyl radical **II**. The radical is cyclized to produce radical **III**, which is trapped by Cu(II) to produce Cu(III) alkyl intermediate **IV**. Following ba ase-catalyzed elimination reaction, product **83** is produced, yielding a Cu(I) species. To re-enter the catalytic cycle, the latter is oxidized to Cu(II) at the electrode.

**Scheme 55:**
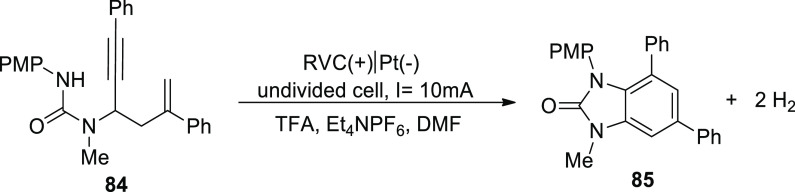
Electrosynthesis of Substituted Benzimidazolone Derivative **85**

In 2019, it has been suggested that by generating homo- and heterocyclic ringed structures a dehydrogenative cyclization cascade can be employed to easily synthesize highly substituted benzimidazolone and benzoxazolone derivatives. The benzimidazolone **85** and benzoxazolone **87** are vital frameworks in a range of pharmacological compounds. A single step of biscyclization/dehydrogenation transforms arylamine-tethered 1,5-enynes into functionalized benzanellated heterocycles with perfect regioselectivity control. H_2_ evolution powers these electricity-powered oxidative processes, eliminating the requirement for oxidants and metal catalysts entirely. Electrochemical dehydrogenative interconversion of readily obtainable urea substrate **84** was examined (Scheme 55). Due to the vulnerability of **86** to base-promoted ionic hydroamidation, base additions were explicitly avoided.

**Scheme 56:**
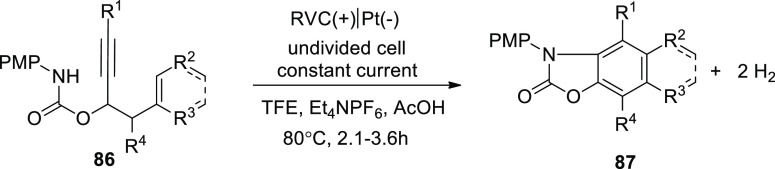
Electrochemical Synthesis of Substituted Benzoxazolones **87**

The substrate, however, maintains its stability in an acidic environment. After conducting an electrolytic procedure at 100 °C researchers were able to produce benzimidazo-lone product **69** in 83% yield. During this process, a constant current of 10 mA, trifluoroacetic acid-additive, dimethylformamide-solvent and RVC-anode and Pt-cathode were employed. To make functionalized benzoxazolones **87** from propargylic carbamates**86**, an electrochemical cyclization cascade could be used (Scheme 56). The yield was significantly improved by performing the electrochemical phenomenon in TFE (solvent) and acetic acid (additive) at 80 °C.^53^

**Scheme 57:**
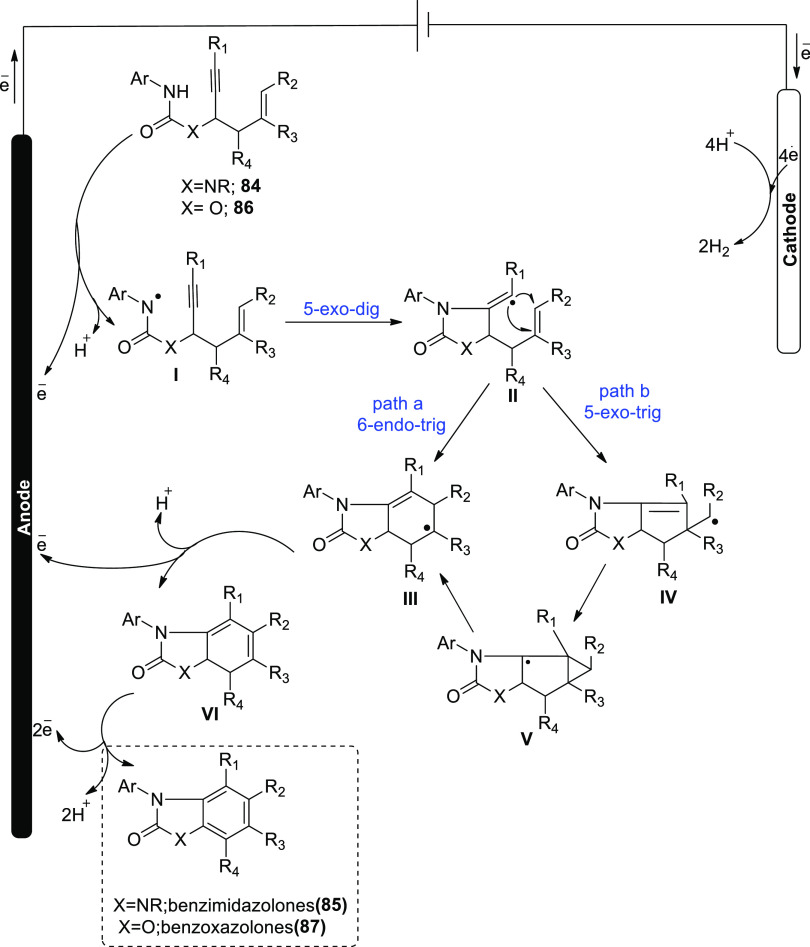
Proposed Mechanism for Electrochemical Synthesis of Substituted Benzoxazolones **85** and **87**

The proposed mechanism for Schemes 55 and 56 has been demonstrated in Scheme 57. In order to create an amidyl radical **I**, the initial material’s arylamine moiety **84** is first anodically oxidized, deprotonated, and then 5-exo-dig cyclized to create a vinyl radical **II**. As a result of **II**’s intramolecular 6-endo-trig cyclization, **III** with an acyclic carbon structure is created. As a substitute, **II** can go through 5-exo-trig cyclization to create **IV**, which can then be changed into **III** through a intermediary atricyclic radical **V**. The typical outcome of the vinyl radical cyclization is a combination of the 5-exo and 6-endo products **85** and **87**.

**Scheme 58:**
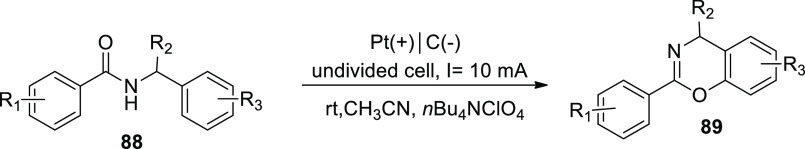
Electrosynthesis of 4*H*-1,3-Benzoxazines **89**

In 2019, electrochemical dehydrogenative cyclization of *N*-benzylamides **88** was examined. A platinum plate anode (+) and a graphite rod cathode (−) and constant current of 10 mA for 2 h was applied in an undivided cell at room temperature. The oxidative degradation of the products was adequately inhibited, and 4*H*-1,3-benzoxazines **89** were produced (Scheme 58) regardless of the benzylic position substituents. This approach could potentially be used to make 4*H*-1,3-benzothiazines **89**.^54^

**Scheme 59:**
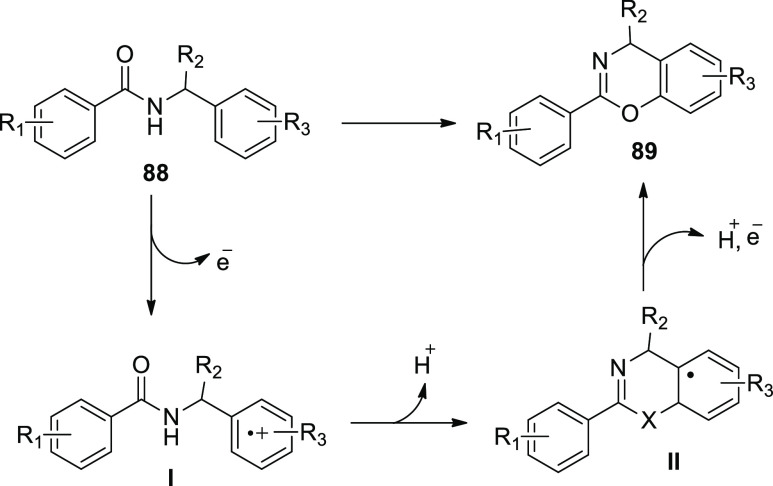
Proposed Mechanism for the Electrosynthesis of 4*H*-1,3-Benzoxazines **89**

The proposed mechanism is illustrated by Scheme 59. The benzylic moeity of the substrate **88** was oxidized under electrolysis conditions to produce the radical cationic **I**, which was then cyclized and deprotonated to produce the intermediate radical **II**. Finally, **II** was oxidized before being rearomatized, yielding the cyclic product **89**.

In 2019, a cupraelectro-catalyzed electrolytic strategy allowed the creation of synthetically relevant isoindolones. In a straightforward undivided cell setup, copper catalyzes the electro-oxidative carbon–hydrogen or nitrogen–hydrogen bond activation of benzamide **90** with terminal alkyne **91**. Flexible, affordable, and nontoxic copper(II) acetate is applied as catalyst. It was discovered through testing that the required isoindolone **92** was produced at 100 °C in DMA using catalytic quantities of Cu(OAc)_2_·H_2_O and NaOPiv as the best additive.^55^

**Scheme 60:**
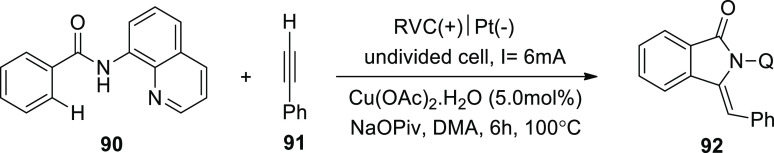
Electrosynthesis of Isoindolone **92**

**Scheme 61:**
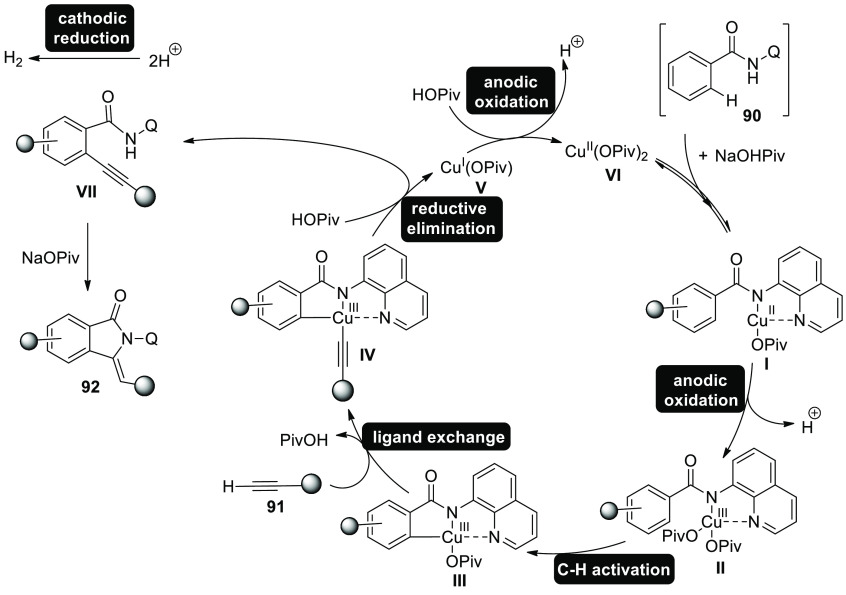
Proposed Mechanism for the Electrosynthesis of Isoindolone **92**

Based on in-depth mechanistic investigations, a workable catalytic cycle was postulated, beginning with substrate **90** coupling and concluding with anodic copper(II) oxidation to yield the active catalytic copper(III) carboxylate species (Scheme 61). The copper(III) intermediate **IV** is then produced by a straightforward C–H activation in the availability of carboxylate on the electron-deficient benzamide **II**. The C–H alkynylated arene **VII** is then produced by metalizing the terminal alkyne **91** with carboxylate help, followed by reductive elimination to fabricate the required isoindolon **92**. The catalytically active copper(III) species is regenerated from the copper(I) complex at the anode.

In 2019, Earth-abundant, low-cost cobalt salts were created to electrocatalyze C–H activation with isocyanides. At room temperature, without using oxidizing agents, the widely available Co-catalysts also pave the way for effective electrooxidative C–H/N–H functionalizations of benzhydrazides **66** using affordable carbon monoxide. By using detachable pyridyl support, the metalla electrocatalysis occurs in a convenient undivided cell design, enabling a step-economical approach to physiologically important imidates **93** and **94**. In general, electricity prohibits equimolar quantities of harmful and expensive d-and f-block elements from activating C–H, which is made possible by an Earth-plentiful aqueous durable cobalt catalyst. The fact that only benzhydrazides **66** permitted the insertion of isocyanide illustrates how difficult the cobalt-electrooxidative C–H activation regime is.^56^

**Scheme 62:**
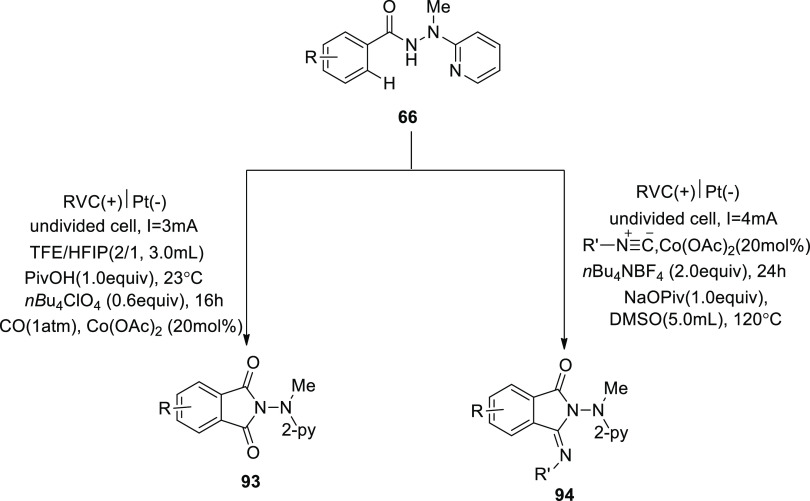
Electrosynthesis of Substituted Cyclic Imidates **93** and **94**

**Scheme 63:**
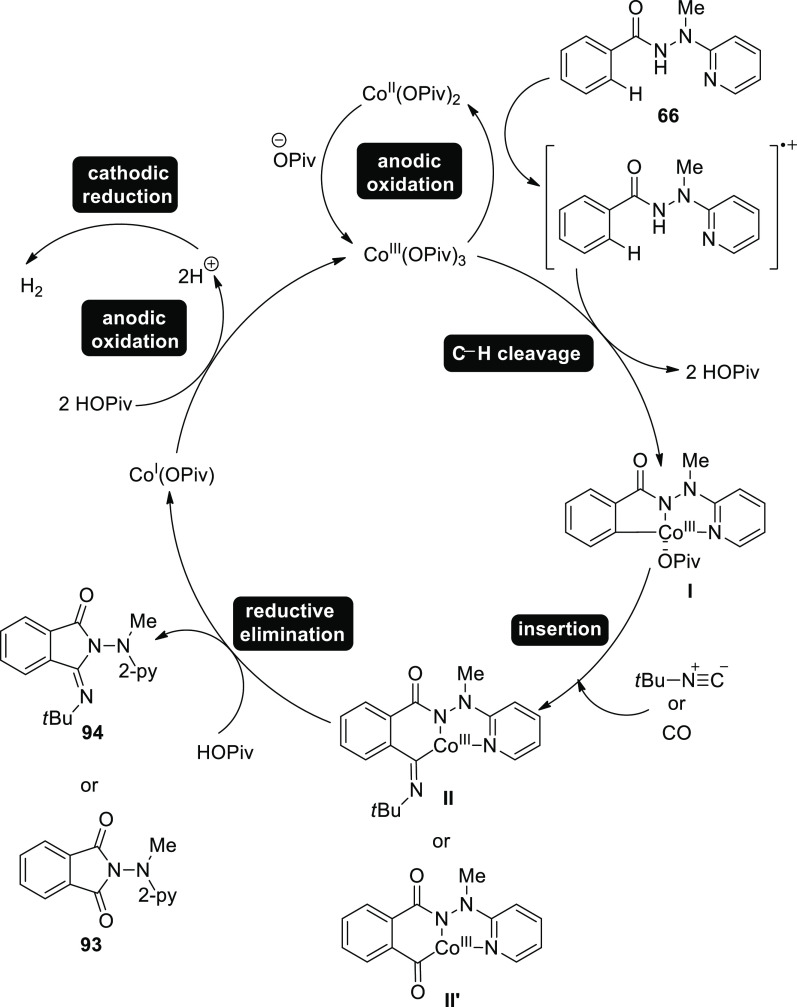
Proposed Mechanism for the Electrosynthesis of Substituted Cyclic Imidates **93** and **94**

It was suggested a realistic catalytic cycle based on mechanistic findings (Scheme 63). Intermediate **II** or **II’**, which is produced by simple carboxylate-assisted C–H activation, and the six-membered cobalta(III) cycle **III** or **III’** is developed. **III**/**III’** is produced by subsequent migratory insertion. The required products **93** or **94** are then produced by reductive elimination, which also creates cobalt(I) species. Finally, anodic oxidation is employed to recreate the catalytically active cobalt(III) carboxylate complex, eliminating the need of hazardous and pricey metals as oxidants and producing just molecular hydrogen as a byproduct.

**Scheme 64:**
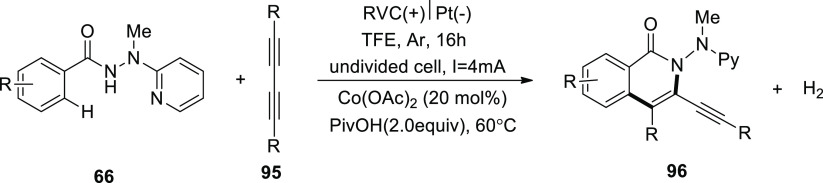
Electrosynthesis of Isoquinolone **96**

In 2019, using affordable, readily available cobalt salts, a comprehensive strategy (Scheme 64) for electrocatalytic carbon–hydrogen/nitrogen–hydrogen annulation with 1,3-diynes **95** was been revealed. Under benign reaction circumstances, the electro-oxidative cobalt catalysis took place in a straightforward undivided cell with exceptional functional group compatibility. Use of electricity eliminates the requirement for hazardous and/or expensive chemicals. Reticulated vitreous carbon-anode and platinum-cathode were used in a reaction that was conducted in TFE (solvent) at 60 °C.^57,58^

**Scheme 65:**
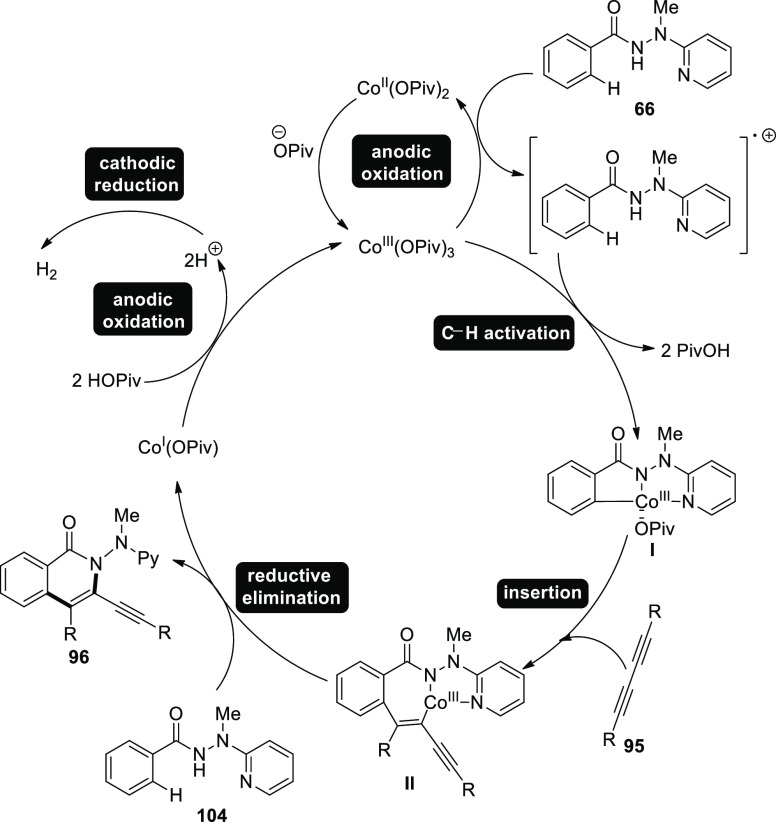
Proposed Mechanism for the Electrosynthesis of Isoquinolone **96**

Based on the primary findings, Scheme 65 demonstrates a plausible mechanism for Scheme 64. In order to make the catalytically effective cobalt(III) salt, anodic oxidation is first used. The production of cobalt(III) species I then follows straightforward C–H cobaltation with carboxylate support. The production of cobalt(III) complex **II** follows migratory insertion, simultaneous release of the cobalt(I) intermediate and the isoquinolone **96** compound, followed by reductive elimination. The catalytically active cobalt(III) carboxylate was regenerated by anodic oxidation. Overall, the cobaltaelectrocatalysis produces hydrogen without employing stoichiometric amounts of costly and dangerous oxidizing chemicals.

Aza-PAHs, which are polycyclic aromatic hydrocarbons doped with nitrogen, have numerous uses in the field of materials science. In 2019, a Rh-catalyzed activation and annulation were used to provide an electrochemical formation of polycyclic aromatic hydrocarbons. The remarkable chemo- and regioselectivity were made possible by the *o*-methylamidoxime’s functions. Amidoxime **97** and diphenylacetylene **98** were used to start the reaction in order to get the desired practice. The target product **99** was isolated using KOAc (base), methanol (solvent), and current of 2 mA. It was shown that addition of a tiny quantity of R-COOH was advantageous, with 1-adamantanecarboxylic acid producing the greatest outcomes. The efficacy was further enhanced by the positively charged rhodium catalyst, which enabled the production of product **99** in a 90% return at 35 °C.^59^

**Scheme 66:**
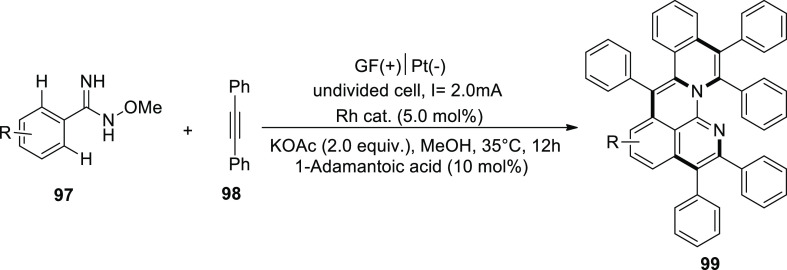
Electrosynthesis of Polycyclic Aromatic Hydrocarbons Doped with Nitrogen (Aza-PAHs) **99**

In 2019, substantial metallaelectro-catalyzed annulations and activation were used. As a result, a range of C–H/N–H functionalizations were viable both intra- and intermolecularly for alkyne **101** annulations and exhibited high degrees of functional group endurance, sensitivity, and selectivity. With a 90% yield, the electrochemical reaction of imidate **100** and asymmetrical alkyne **101** produced the required isoquinoline **102**. When methanol (CH_3_OH) is used as the solvent, (pentamethylcyclopentadienyl)rhodium(III) dichloride dimer is used as a catalyst, and sodium pivalate (NaOPiv) and pivalic acid (PivOH) are added, while platinum plate (Pt) and graphite felt (GF) serve as negatively and positively charged electrodes correspondingly, in an indivisible condition of the cell.^60^

**Scheme 67:**
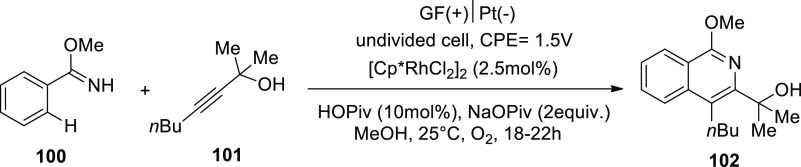
Electrosynthesis of Isoquinoline **102**

**Scheme 68:**
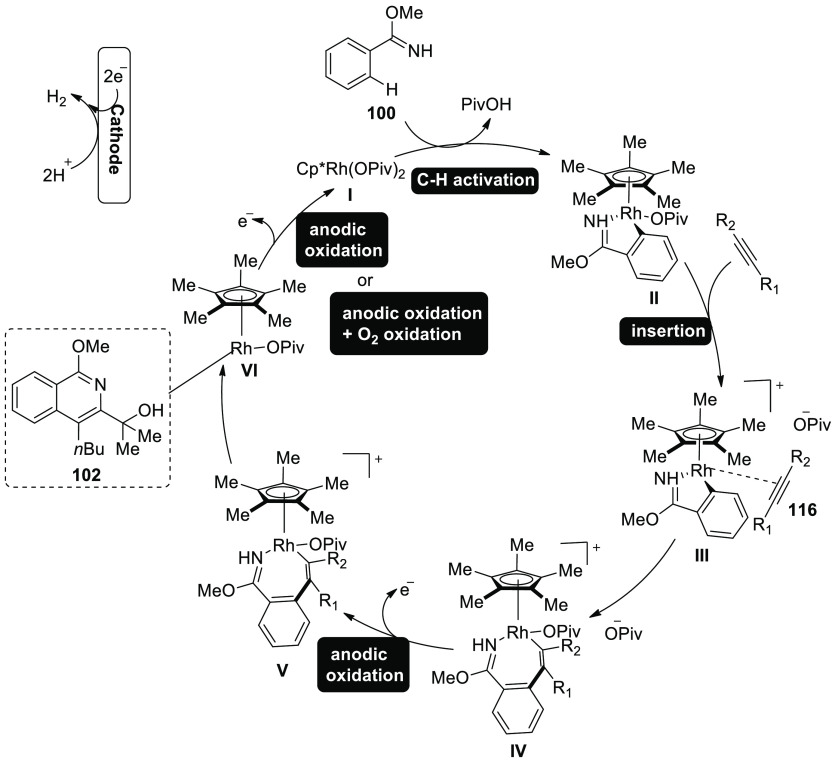
Proposed Mechanism for the Electrosynthesis Ofisoquinoline **102**

A conceivable mechanism is suggested in Scheme 68. Cp*Rh(OPiv)_2_**I** is produced by the reaction between sodium pivalate and catalyst precursor, which subsequently combines with substrate **100** to produce the cyclometalated complex **II** through simple C–H activation. Rhoda(III) cycle **IV** is then produced via migratory insertion and alkyne coordination **III**. Rhoda(IV) cycle **V** swiftly produces intermediate **VI** after anodic-oxidation. Catalyst **I** is then regenerated via anodic oxidation, which can be sped up by oxygen. Aerobic and anodic oxidation appear to be plausible processes for reoxidation of Rh^II^–Rh^III^. Rhoda(III) cycle **IV** can supply product **102** directly together with a reduced Rh (rhodium) that may reoxidize. Cathodic proton reduction produces only molecular hydrogen as a stoichiometric byproduct, and rigorous headspace GC analysis has proven this to be the case.

**Scheme 69:**
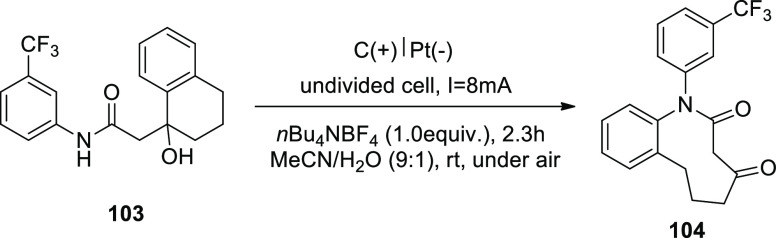
Electrosynthesis of 9-Membered Lactam **104**

In 2019, a direct electrochemical synthesis using C–C bond cleavage without the use of a catalyst has been established (Scheme 69). The best results were obtained by directly electrolyzing substrate **103** at a constant current (8 mA) in a blended electrolyte solution of MeCN/H_2_O and *n*Bu_4_NBF_4_. The required 9-membered lactam **104** was extracted in 98% yield at room temperature avoiding the inclusion of extra bases or catalysts.^61^

**Scheme 70:**
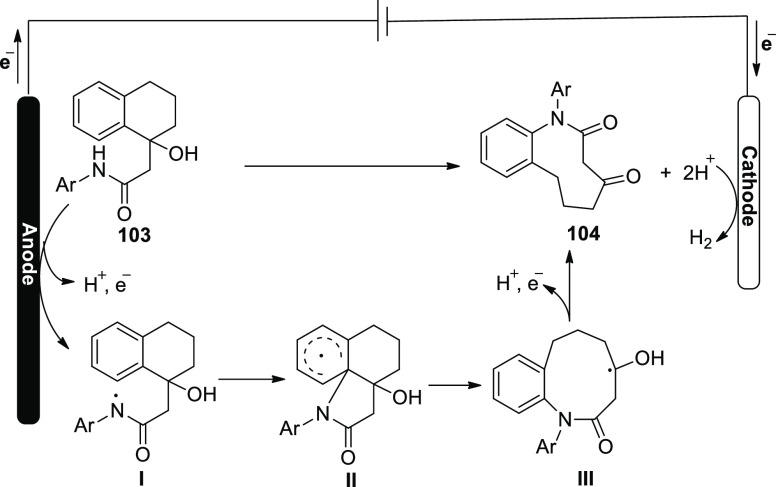
Proposed Mechanism for the Electrosynthesis of 9-Membered Lactam **104**

A viable mechanism is proposed based on our mechanistic findings (Scheme 70). The N–H bond in **103** is first anodically oxidized, producing intermediate **I**. This intermediate faces cyclization to form radical **II**, which undergoes selective C–C bond cleavage to produce radical **III**. At the end, the medium-sized lactam **104** would be produced by oxidizing this ketyl radical **III** by a single electron and then losing a proton. However, the cationic pathway cannot be ruled out because of the two closely spaced oxidative waves of **103** in the cyclic voltammogram.

**Scheme 71:**
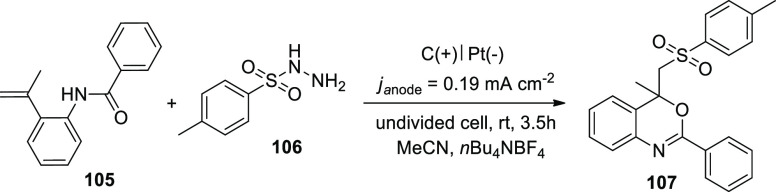
Electrosynthesis of Sulfonated 4*H*-3,1-Benzoxazines **107**

In 2020, He and co-workers reported a good method for the electrochemical manufacturing of sulfonated 4*H*-3,1-benzoxazines. Starting from *N*-(2-(prop-1-en-2-yl)phenyl)benzamide **105** and *p*-toluenesulfonylhydrazine **106** sulfonated 4*H*-3,1-benzoxazine **107** have been obtained in 78% yield (Scheme 71). The best result was obtained by conducting the reactions in an undivided cell with a carbon rod (anode) and a platinum foil (cathode) in anhydrous MeCN possessing *n*Bu_4_NBF_4_ as the electrolyte in a galvanostatic condition at room temperature. This method has a wide substrate scope with varied functional group tolerance at air temperatures, free of metal and exogenous oxidants.^62^

**Scheme 72:**
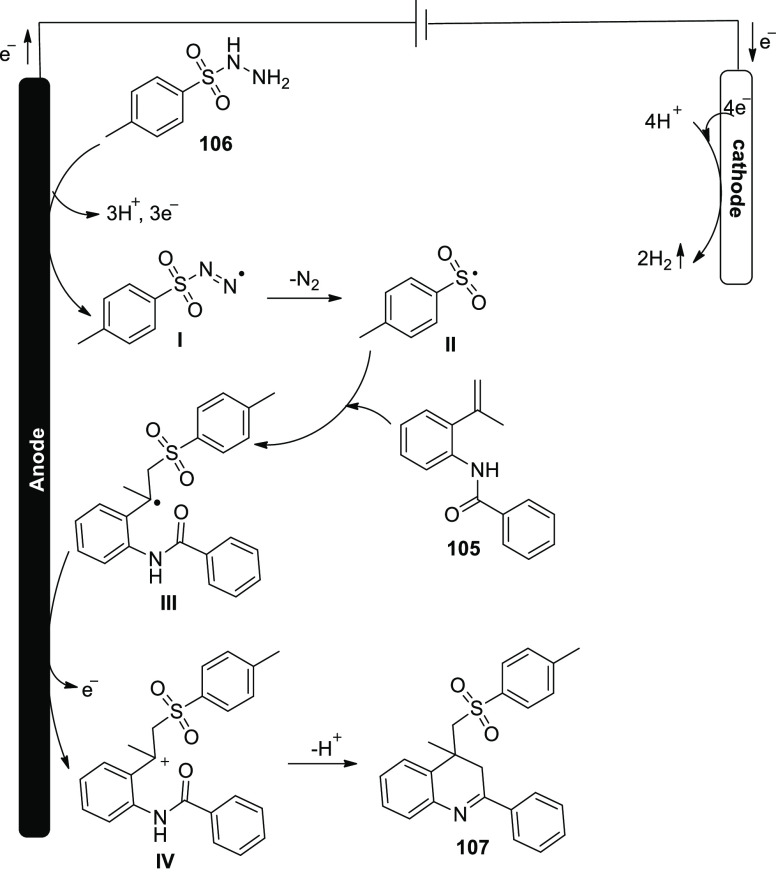
Proposed Mechanism for the Electrosynthesis of Sulfonated 4*H*-3,1-benzoxazines **107**

Electro-oxidation and deprotonation of *p*-toluenesulfonyl hydrazide **106** resulted in the formation of the corresponding sulfonyl radical **II**, which was followed by the release of N_2_. The sulfonyl radical **II** was then combined with alkene**105** to produce the radical intermediate **III**. The radical intermediate **III** might then be immediately oxidized by anode to produce the appropriate carbon cation **IV**. The required sulfonated 4*H*-3,1-benzoxazine**107** was produced after the nucleophilic attack and deprotonation. Protons were simultaneously transformed to H_2_ via cathodic reduction (Scheme 72).

**Scheme 73:**
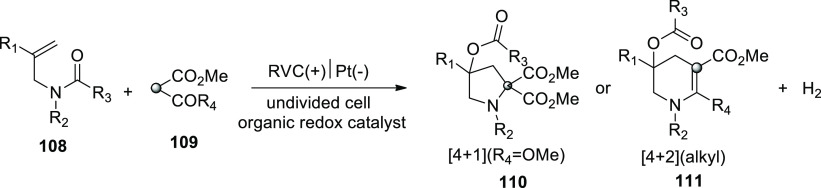
Electrosynthesis of Pyrrolidines **110** and Tetrahydropyridines **111**

Similarly, in 2018 and 2020, He and co-workers reported the fabrication of pyrrolidines **110** and tetrahydropyridines **111** derivatives through a dehydrogenative annulations process by employing widely accessible ingredients (Scheme 73). In this approach, the employment of a catalytic amount of phenothiazine-based redox catalyst helps in effective and selective 1,3-dicarbonyl compound intermolecular radical reactions. It is being considered to use this strategy to encourage different oxidative radical events of 1,3-dicarbonyl compounds.^63,64^ The electrolysis of N-allyl amide **108** with dimethyl malonate **109** is performed in this work. The best reaction system was determined to be HCO_2_Na (0.3 equiv) as the base additive and phenothiazine (20 mol %) as the redox catalyst in a refluxed mixture of *t*BuOMe, MeCN, and H_2_O in a 20/3/1 ratio. The pyrrolidine derivative **110** was obtained (yield = 70%) under these circumstances. An RVC anode and Pt cathode were used. When a β-ketoester (coupling partner) was used, the resulting (4 + 2) annulation yielded a tetrahydropyridine product **111** with no pyrrolidine derivative **110**. The annulation of methyl acetoacetate(β-ketoester) with an enyne was the only exception, yielding 13% of pyrrolidine and 53% of tetrahydropyridine product. It is worth noting that these annulations did not require sodium formate and profitted from lower current densities.

**Scheme 74:**
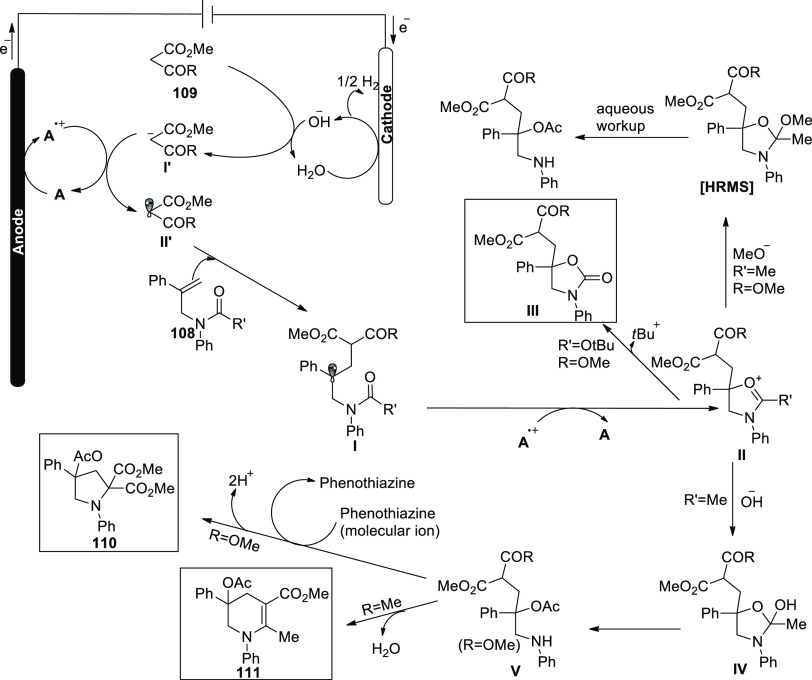
Proposed Mechanism for the Electrosynthesis of Pyrrolidines **110** and Tetrahydropyridines **111**

Based on the findings of this study, a favorable mechanistic approach has been presented in Scheme 74. The anodic oxidation of **A** to produce the radical cation **A**^**•+**^ is the first step in the process. Meanwhile, at the cathode, reduction of water to hydroxide ion and hydrogen gas occurs, with the latter deprotonating the 1,3-dicarbonyl molecule **109** to create the more oxidizable anion **I’**. Single-electron transfer produces a C-radical **II’**, which on further combination with alkenyl moiety of the N-allyl amide **108** to produce a tertiary C-radical **I**. The carbonyl group of intermediate **I** is oxidized and trapped intramolecularly instead of the carbonyl group of the 1,3-dicarbonyl moiety to provide **II**. The equivalent intermediate **II** on releasing tertiary butyl group provides a cyclic carbamate **III**. For amides, **II** reacts with water or hydroxide ion to form a secondary amine **V**, which would then undergo a C(sp^3^)–H/N–H cross coupling reaction (R = OCH_3_) to yield the product **110** or intramolecular dehydration (R = CH_3_) to yield tetrahydropyridine **111**.

**Scheme 75:**
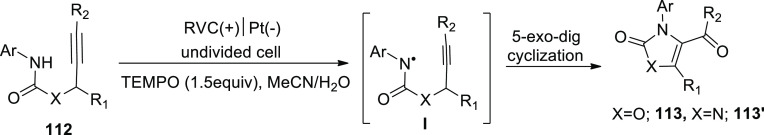
Electrosynthesis of Oxazol-2-ones **113** and Imidazol-2-ones **113’**

In 2020, 5-exo-dig cyclization of amidyl radicals **I** was used to establish oxazol-2-ones **113** and imidazol-2-ones **113’** (Scheme 75). The electrosynthesis is based on TEMPO’s dual function as an oxygen-atom donor and a redox facilitator for the development of amidyl radicals **I**. The reactions are carried out in a simple setup under mild conditions.^65^

**Scheme 76:**
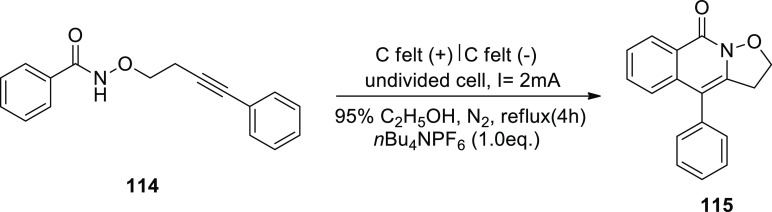
Electrosynthesis of Isoxazolidine-Fused Isoquinolin-1(2*H*)-ones **115**

In 2020, the electrochemical formation of isoxazolidine-fused isoquinolin-1(2*H*)-ones **115** via amidyl radicals has been described as a general and useful process (Scheme 76). For the optimization experiment, *N*-((4-phenylbut-3-yn-1-yl)oxy)benzamide **114** was selected as a template molecule. Under 2 mA (constant current) and *n*Bu_4_NBF_4_ (electrolye), the desired oxidation-induced intramolecular annulation product **115** was formulated in 93% yield.^66^

**Scheme 77:**
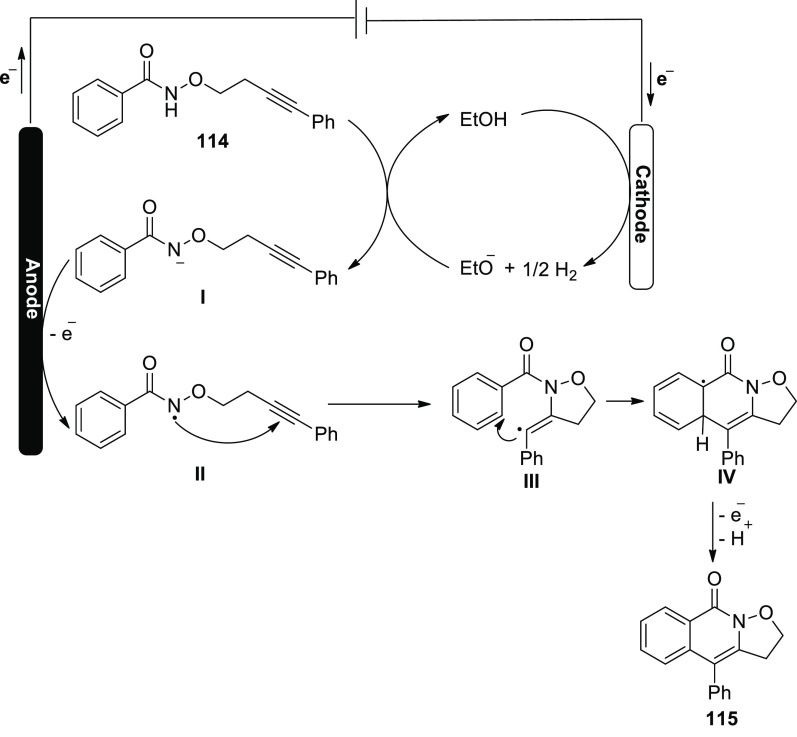
Proposed Mechanism for the Electrosynthesis of Isoxazolidine-Fused Isoquinolin-1(2*H*)-ones **115**

In Scheme 77, a feasible mechanism is offered. First, ethanol is reduced cathodically to make ethoxide ion, which deprotonates **114** to produce anion **I**. Next, the anion **I** undergoes SET-oxidation to manufacture radical **II**. **II** takes part in the 5-exo-dig annulation to produce radical **III**, and then the delocalized radical **IV** is produced by a second annulation. Finally, the rearomatization of **IV** by electron oxidation forms product **115**.

**Scheme 78:**
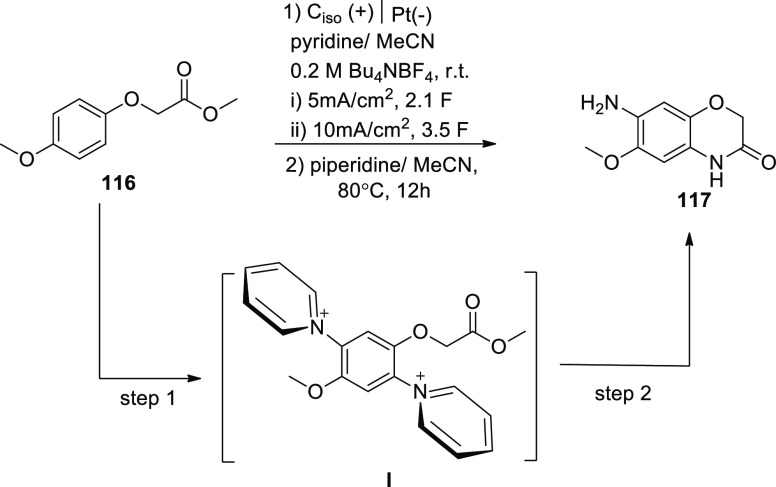
Electrosynthesis of 2*H*-Benzo[b][1,4]oxazine-3(4*H*)-one **117**

In 2020, the introduction of a versatile and powerful approach for electrochemically generating doubly positive (+ve) charged Zincke intermediate **I** is described. Phenoxy-acetate**116** demonstrated intriguing reactivity toward 2-fold amination products when it was used. Amines that stimulate the formation of an intramolecular heterocycle **117** are liberated by these pyridinium intermediates **I**. A two-step amination technique with a 90% yield was discovered (Scheme 78). This method does not need metal catalysts or leaving groups, making it a novel and strong method. Divided cell arrangement with isostatic graphite-anode and platinum-cathode, Thomapor-separator, and a two-step electrolysis of 3.4 F with 5 mA/cm^2^ and 2.1 F with 10 mA/cm^2^ at 25 °C are the optimal electrolysis conditions.^67^

**Scheme 79:**
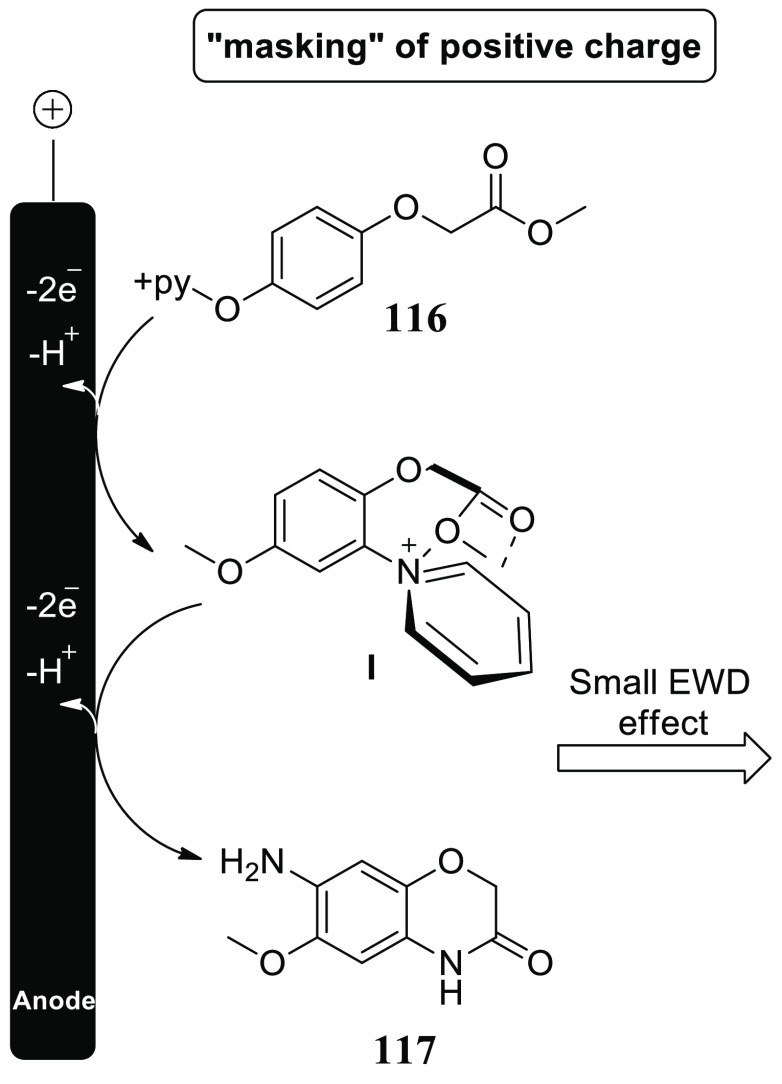
Proposed Mechanism for the Electrosynthesis of 2*H*-Benzo[b][1,4]oxazine-3(4*H*)-one **117**

In the presence of pyridine, anodic oxidation of **116** generally results in positively charged and relatively stable pyridinium intermediates **I**. Species **I** extracts an aromatic primary amine group in a second step after being treated with pyridine. The phenoxy acetate derivative **116**’s ester functionality may be effective to stabilize **I** by π–π, π–nonbonding, or nonbonding-cationic connections. Further oxidation may benefit from stabilization of the electron-deficient substituent. These contacts function as masking the positive (+) charge, shielding effects, reducing electron-withdrawing impact and allowing for more oxidation of **I** (Scheme 79).

**Scheme 80:**
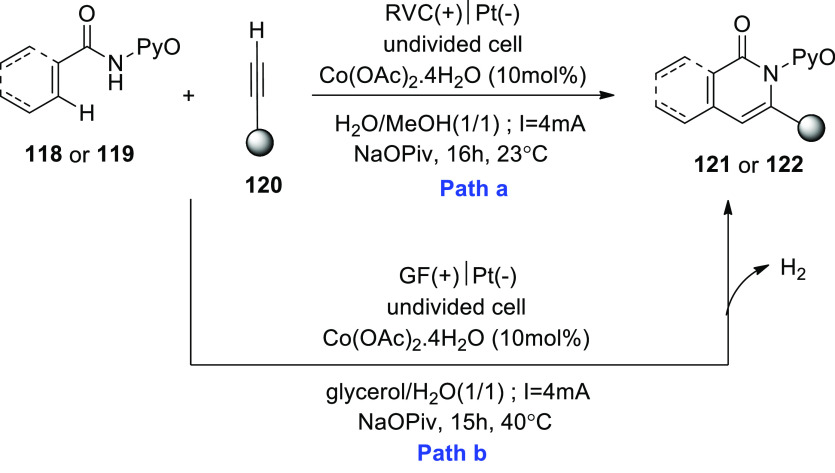
Electrosynthesis of Isoquinolones **121** and Pyridones **122**

In 2020, C–H functionalizations catalyzed by cobalt have become a revolutionary framework for molecular synthesis. By creating biologically significant isoquinolones **121** and pyridones **122** from the respective aryl and alkenyl amides **118** or **119** produced from pyridine N-oxide in a carbon–hydrogen or nitrogen–hydrogen activation process, cobaltaelectrocatalysis was further shown to have potential (Scheme 80). The transformation was detectable at room temperature under incredibly moderate reaction conditions and high yields in a H_2_O-containing solvent mixture. An undivided cell with a platinum (Pt) cathode and reticulated vitreous carbon (RVC) anode is used to drive the operation at 23 °C for 16 h. As a result, a method for a full resource economy has been created, using renewable green power as a redox agent that produces only valuable hydrogen under redox mediator-free conditions.^68−70^ The identical reaction conditions (Scheme 80-Path a) were used, but with a GF anode in place of an RVC anode, and yields of 70–76% of the product were achieved.^71^

The use of glycerol produced from biomass as a reaction system for electro-enabled C–H activation pathways has never been documented. Direct application of renewable energies for the synthesis of C–C or N–C bonds, omitting molecular hydrogen(byproduct), supported the resource economy. As a result, the sustainable C–H activation of amides was made possible without applying harmful metal oxidants by a cobalt(Co) catalyst free of Cp*. At 40 °C, the moderate C–H/N–H functionalization was easily accomplished in aqueous glycerol. Importantly, it has been shown that electrocatalytic C–H activations can be carried out directly using renewable solar and wind energy. Molecular catalysis should benefit the development of more sustainable future energy economies by combining renewable solvents and alternative energy sources (Scheme 80-Path b).^72^

**Scheme 81:**
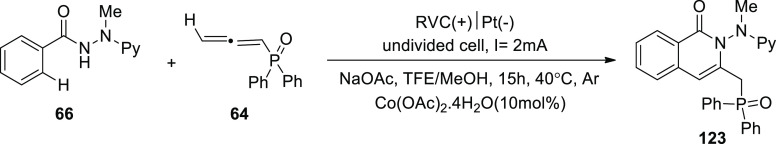
Electrosynthesis of C–H Annulation Product **123**

Similarly, electro-oxidative cobalt catalysis was made possible in 2020 (Scheme 81). Electrochemical C–H activations of **66** with allene **64** were undertaken in an undivided cell arrangement with high degrees of chemoselectivity and regioselectivity. When 2,2,2-trifluoroethanol or methanol was utilized as the solvent, it produced the desired regioselective C–H annulation product **123** in 91% yield. When synthesis was accomplished at 40 °C, the yield was considerably increased. Analyzing substitute additives showed that NaOAc performed marginally better than NaOPiv and PivOH.^73^

**Scheme 82:**
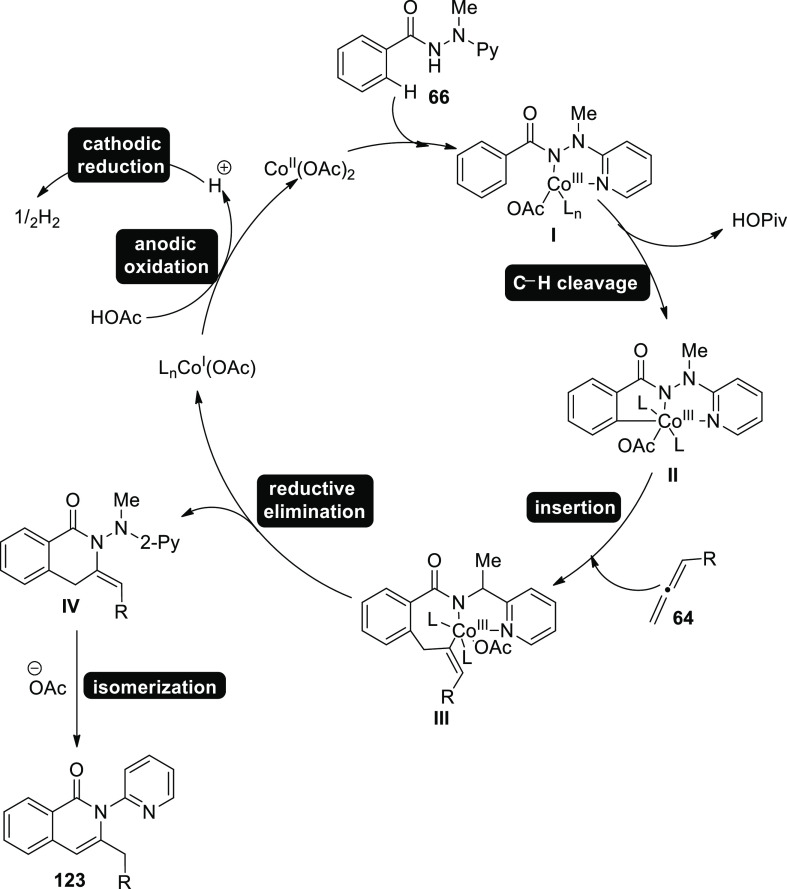
Proposed Mechanism for the Electrosynthesis of C–H Annulation Product **123**

A reasonable catalytic cycle was proposed and is shown in Scheme 82 based on the aforementioned mechanistic results. Anodic oxidation served as the catalyst for the electrooxidative C–H activation, which was then followed by a carboxylated aided BIES C–H cobaltation to produce cobalt(III) complex **II**. The cobalt(I) complex and the exomethylene isoquinolone **IV** were then produced by the regioselective allene insertion and subsequent reductive elimination, which could then be isomerized to produce the desired product **123**. The key anode oxidation was then used to reconstruct the catalytically competent cobalt(III) complex **I**, which completed the catalytic cycle. Overall, the coboltaelectrocatalysis method avoided using chemical oxidants, and the sole waste generated was molecular hydrogen.

In 2020, it has been discovered that imidazoles **124** and alkynes **98** can undergo a ruthenium(Ru)-catalyzed electrosynthetic pathway to develop a variety of N-fused[5,6]-bicyclic heteroarenes **125** by using electrochemical C–H/N–H annulation selectively and sensitively in the absence of metallic oxidizing chemicals. An indivisible cell arrangement with a Pt-cathode and GF-anode is used to produce the desired product **125**. However, DMF produced the best results when compared to other solvents.^74^

**Scheme 83:**
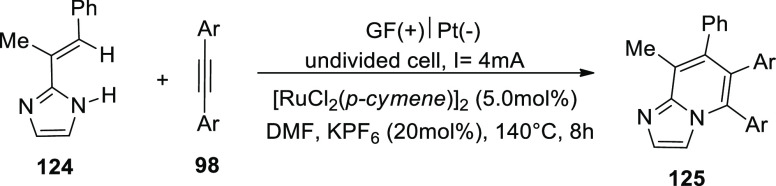
Electrosynthesis of Bridge-Head N-Fused [5,6]-Bicyclic Heteroarenes **125**

**Scheme 84:**
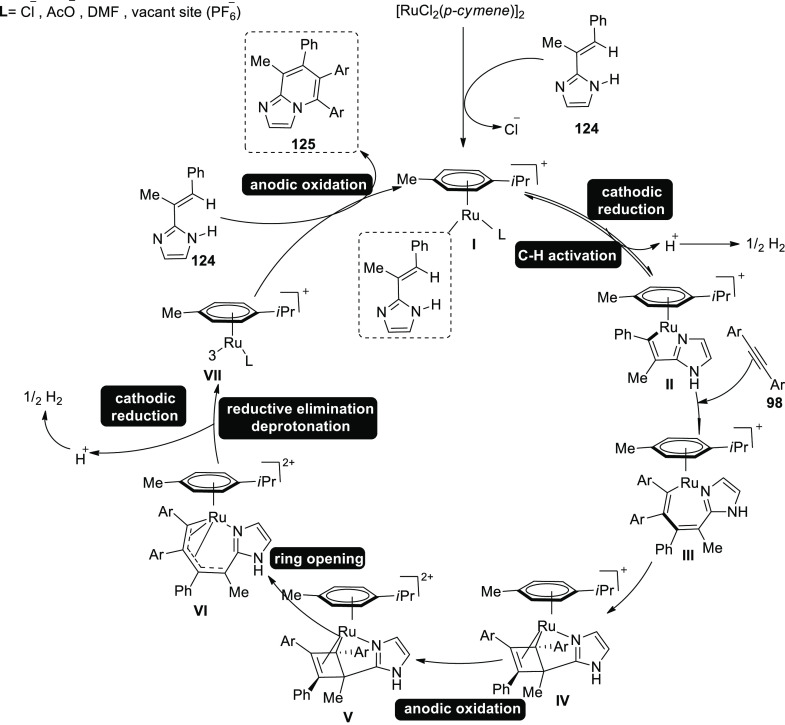
Proposed Mechanism for the Electrosynthesis of Bridge-Head N-Fused [5,6]-Bicyclic Heteroarenes **125**

It is suggested that the catalytic cycle start with a quick organometallic C–H activation based on our mechanistic findings (Scheme 84). As a result, **II** is produced. The azaruthenabicyclo[3.2.0]heptadiene **IV** is then produced via alkyne coordination and migratory insertion, which is then anodized to produce the ruthenium(III) complex **V**. **VI** is produced by a ring opening of **V**. Ruthenium(I) complex **VII** is created by oxidation-induced reductive elimination and is subsequently anodically reoxidized.

**Scheme 85:**
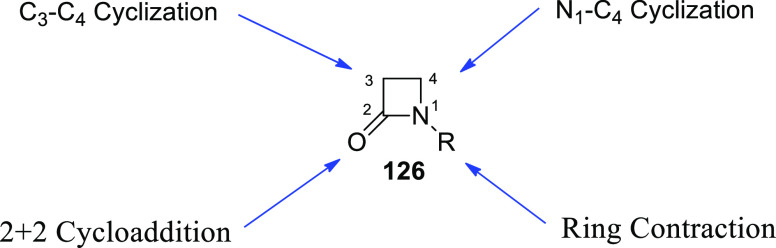
Synthesis of Azetidin-2-ones **126**

In 2021, the interconversion of a linear molecule bromoamide into cyclic structure **126** was made (Scheme 85). The heterocyclic compounds known as β-lactams (azetidin-2-ones)**126** have 4-members and an amide moiety. It is not essential to go into detail about the significance of this group of substances in the field of antibiotics because they are so well-known in biomedical sciences.^75^

**Scheme 86:**
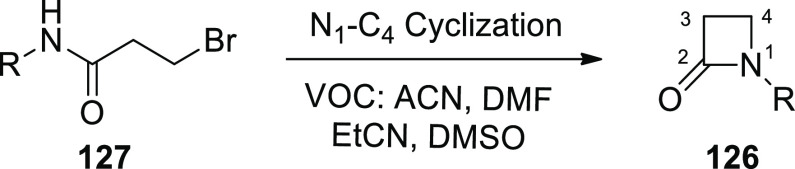
Electrochemical Synthesis of Azetidin-2-ones **126**

Using constant current electrolysis in a suitable solvent (VOC)-supporting electrolyte(tetraalkylammonium salt) solution and the addition of bromoamides **127**, a facile electrochemical synthesis of β-lactams has been achieved. The electrogenerated base is produced in this case. This method avoids the need for bases and probases, resulting in high yields of β-lactams **126** (Scheme 86). Under usual cases, **127** with a leaving group in the β-position can undergo deprotonation at the N atom, producing a negative ion that provides internal nucleophilic displacement to the analogous β-lactams.

**Scheme 87:**
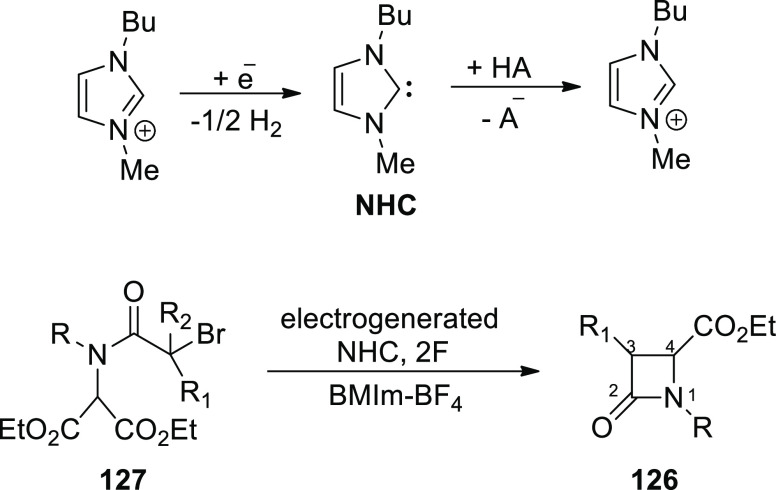
Electrosynthesis of Azetidin-2-ones **126**

By deprotonating a carbon atom, a linear bromoamide **127** can be cyclized. In this scenario, appropriate acidity must be achieved by using ethoxy-carbonyl (an electron withdrawing group). Reduction of BMIm^+^ at the negative electrode generates N-heterocyclic carbene (Scheme 87). This NHC can serve as a nucleophile or as a base, deprotonating the bromoamide **127** and causing ring formation. These carbenes have proven to be effective and environmentally acceptable tools for performing organic synthesis. Because no probase is required except the IL solvent, this electrochemical approach provides for a simpler process. In reality, the NHC generates the initial IL cation when it functions as a base. The yields of β-lactams **126** are likewise acceptable when the internal displacement takes place at a disubstituted carbon–bromine site, irrespective of the nitrogen end.

**Scheme 88:**
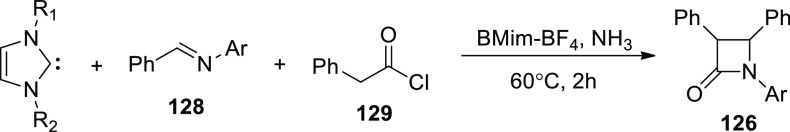
Electrosynthesis of Azetidin-2-ones **126**

[2 + 2] Cycloaddition (staudinger reaction) between imine **128** andketene is the most well-known route to make β-lactams **126** (Scheme 88). The ketene, which is generally unstable, is frequently produced by dehydrohalogenating a suitable acyl halide **129** in situ. The mechanism of this reaction is still being discussed, despite the fact that it was initially documented in 1907. Furthermore, the stereochemical result is not unambiguous.

**Scheme 89:**
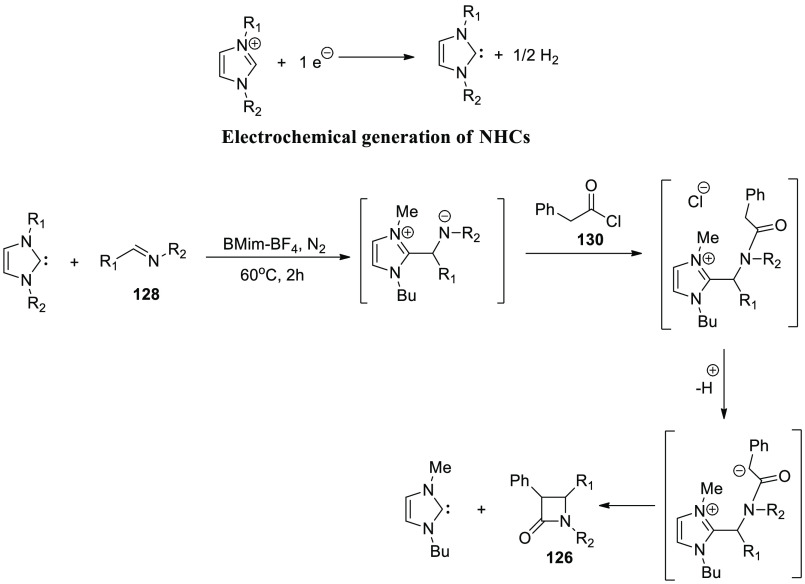
Proposed Mechanism for the Electrochemical Synthesis of Azetidin-2-ones **126**

Due to the unpredictable nature of the process, the stereochemistry consequence of the event is not disregarded. In fact, with differing relative configurations in the final β-lactam **126**, either a concerted or two-step process is feasible. In the electrosynthesis of IL BMIm-BF_4_, cis-lactams were generated preferentially, based on the N atom’s configuration, a fluctuating proportion of NHC necessitated the inclusion of Et_3_N (an external base) in order to get satisfactory yields. Electrogenerated NHC acted as both a base and a nucleophile in this reaction, activating the imine **128** (Scheme 89) and ruling out a coordinated process.^76^

**Scheme 90:**
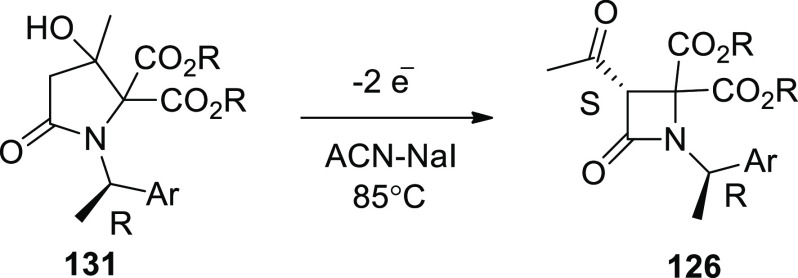
Electrochemical Synthesis of Azetidin-2-ones **126**

The last electrochemical process involved in the synthesis of β-lactams **126** is anodically induced ring contraction. The electrochemical technique foresaw the possibility of creating the active “I^+^” in situ via anodic oxidation of iodide from the supporting electrolyte in addition to unintentionally establishing an EGB at the cathode. Reaction was carried out in acetonitrile at 80 °C in the presence of chiral pyrrolidones **131** to give excellent yield (Scheme 90).

**Scheme 91:**
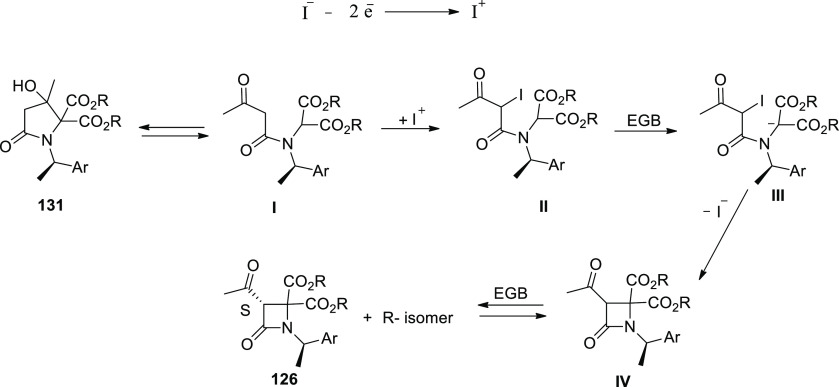
Proposed Mechanism for the Electrochemical Synthesis of Azetidin-2-ones **126**

In Scheme 91, the proposed mechanism is described. The iodide anion is the electroactive species, which gives “I^+^” on oxidation that can react with the open form of **I** to produce the equivalent α-iodide. The homologous β-lactam **126** is produced by intramolecular iodide translocation and deprotonation by a base, most likely in equimolar R- and S-forms at C_3_. Equilibration in basic media creates S-diastereoisomer as a major product, which may be extracted in pure form following crystallization because it is the most thermodynamically stable isomer.

**Scheme 92:**
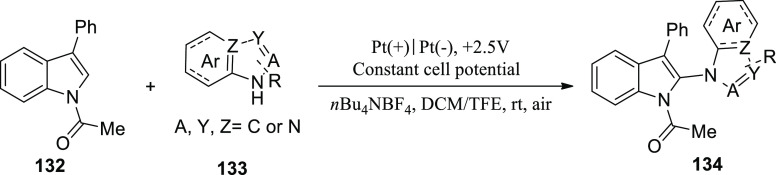
Electrosynthesis of 2-Heteroaryl-1-(3-phenyl-1*H*-indol-1-yl)ethan-1-one **134**

In 2021, Zhou et al. demonstrated a unique electro-oxidative C–N bond production approach that is accessible to gram scale synthesis and facilitates many substances (Scheme 92). The model substrate 1-(3-phenyl-1*H*-indol-1-yl)ethan-1-one **132** was first coupled with substituted pyrazole **133** adopting an undivided cell with platinum acting as both a cathode and anode. Using a mixture of DCM/TFE as the solvent, *n*Bu_4_NBF_4_ as electrolyte, 0.5 mmol of **132**, and 0.5 mmol of **133** at room temperature under air, the optimal condition was eventually established. The target product **134** possessing 69% yield was separated.^77^

**Scheme 93:**
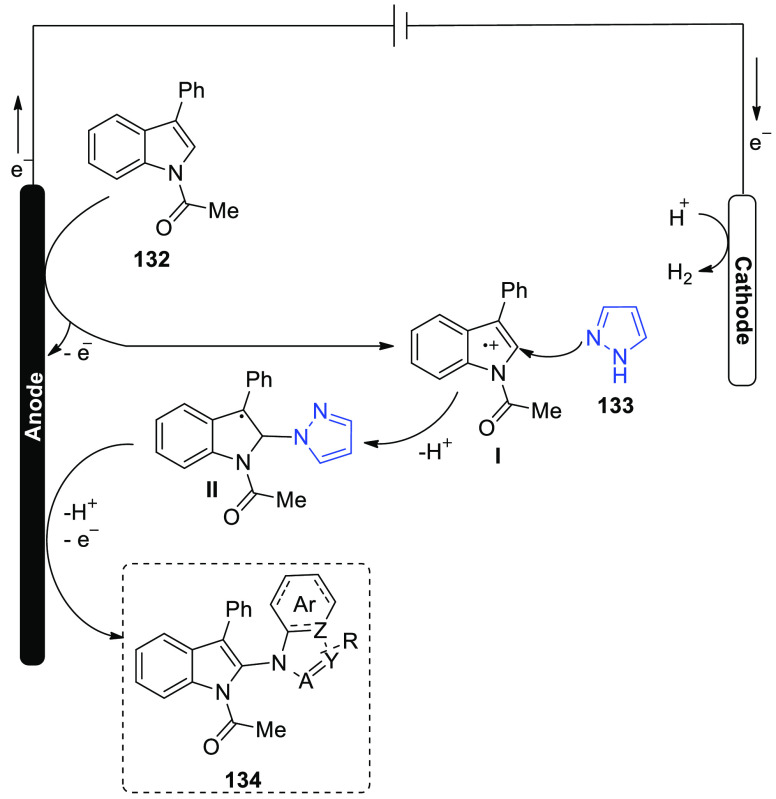
Electrosynthesis of 2-Heteroaryl-1-(3-phenyl-1*H*-indol-1-yl)ethan-1-one **134**

A viable mechanistic method for electrochemical oxidative azolation of indoles **132** was presented depending on mechanistic research and relevant literature publications (Scheme 93). Because indole **132** has a lower oxidative potential than pyrazole **133**, it first undergoes anodic oxidation, resulting in intermediate **I**, which then undergoes nucleophilic addition with **132** to lose a proton, resulting in radical intermediate **II**. To get the required azolated product**134**, the radical intermediate will be further oxidized and tautomerized.

### 2.3 O-Heterocycles

O-Heterocycles are an important family of bioactive molecules. One reason for this could be their natural abundance and diverse biological functions^78^ which are used in the development of new drugs.^79^

**Scheme 94:**
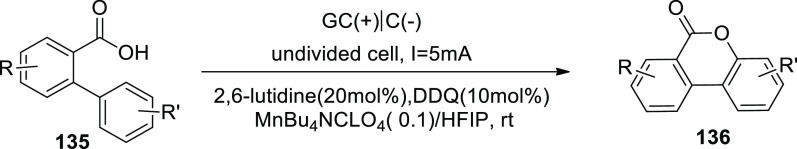
Electrosynthesis of 6*H*-Benzo[c]chromen-6-ones **136**

Recently a family of *o*-heterocycles have been published by Li and co-workers in 2018 (Scheme 94). This electro-synthesis uses 2,3-dichloro-5,6-dicyano-1,4-benzoquinone (DDQ) as redox-mediator and low-cost glassy carbon (GC) electrodes to enable intramolecular lactonization of biphenyl-2-carboxylic acid derivatives **135** to produce a large family of 6*H*-benzo[c]chromen-6-ones **136** in excellent yield.^80^ The reaction was carried out at room temperature with biphenyl-2-carboxylic acid **135** as a model substrate in an undivided cell with MeCN/LiClO_4_electrolyte solution yielding the required compound **136**.

This chain starts with 2,3-dichloro-5,6-dicyano-1,4-benzoquinone performing a homogeneous transfer of electrons (e^–^) from **135** to radical **I**, which then allowed DDQ to regenerate after anodic-oxidation,. When radical **I** is added to an arene ring, intermediate **II** is formed. The aryl radical **II** was then anodically oxidized again, yielding **136**. The presence of 2,6-lutidine would favor the coupled electron-transfer by facilitating the deprotonation of both **135** and intermediate **II**.

**Scheme 95:**
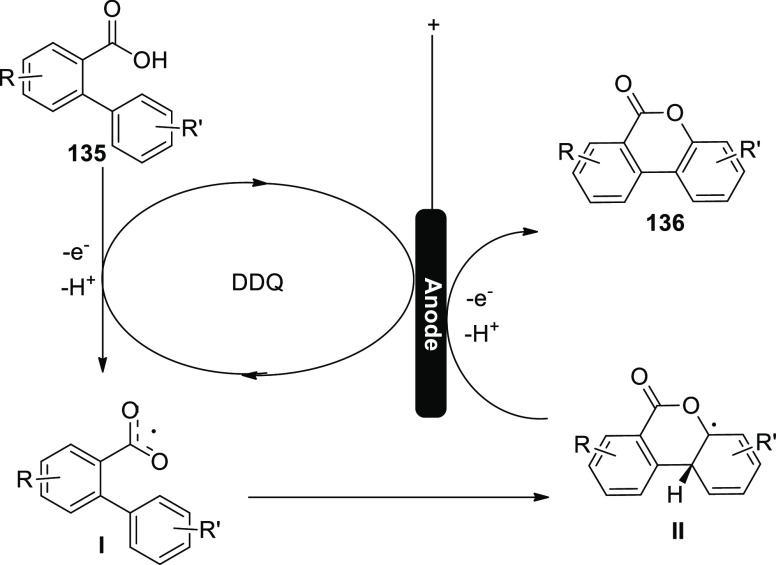
Proposed Mechanism for the Electrosynthesis of 6*H*-Benzo[c]chromen-6-ones**136**

**Scheme 96:**
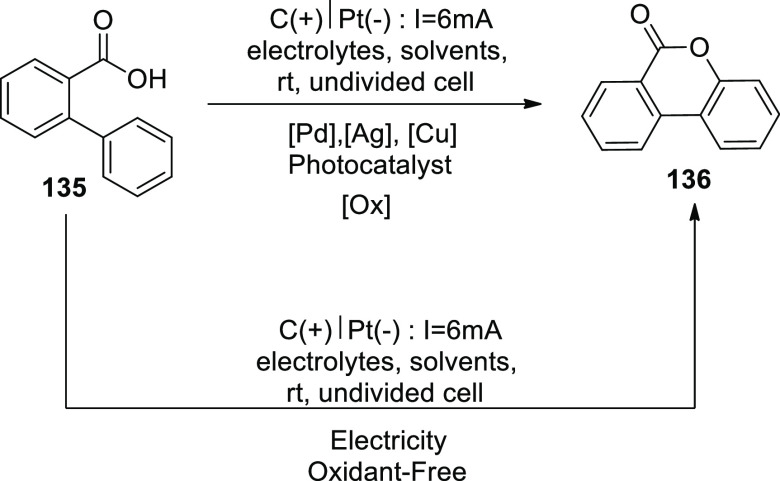
Electrosynthesis of 6*H*-Benzo[c]chromen-6-one **136**

In the same context, the electrochemical oxidative coupling has been reported by Shao and co-workers in 2018 (Scheme 96). Lactones **136** have been electrochemically synthesized by 2-arylbenzoic acids **135**. Carbon–hydrogen or oxygen–hydrogen cross-coupling reaction of 2-phenylbenzoic acid **135** can be achieved under mild circumstances with and without using excess oxidants and metallic entities and using anode oxidation and cathode hydrogen evolution in a simple undivided cell. Different 2-arylbenzoic acids could provide the respective lactones in 30% to 90% yields by using a cheap and green Na_2_SO_4_ aqueous as a supporting electrolyte. This reaction has a high usefulness for natural product synthesis and excellent reaction efficiency on a gram scale.^81^

**Scheme 97:**
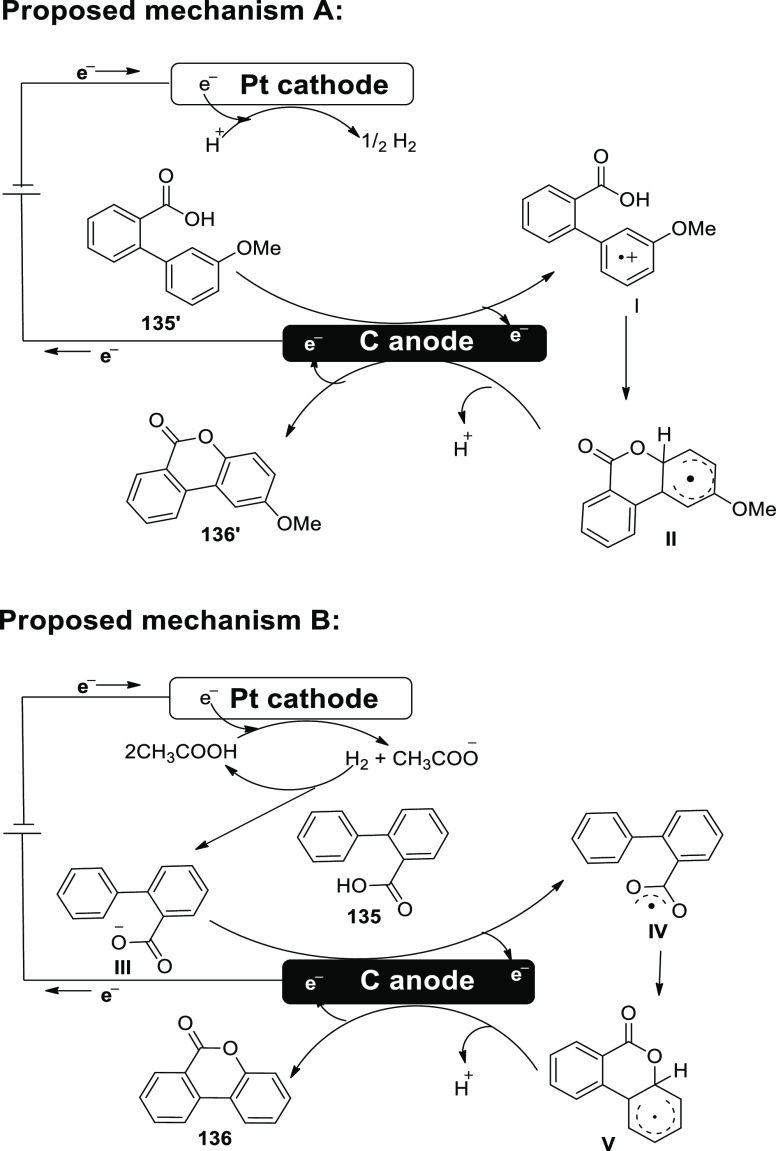
Proposed Mechanism for the Electrosynthesis of 6*H*-Benzo[c]chromen-6-one **136** and Methoxy Derivative of 6*H*-Benzo[c]chromen-6-one **136**’

Mechanism A is only applicable to some substrates containing a methoxy group, such as **135’**. The radical cation **I** is formed when a positively charged electrode (anode) performs SET oxidation of **135’**. The produced aromatic ring radical cation is next attacked by the carboxyl group, resulting in the intermediate **II**. The radical adducts **II** is quickly aromatized and deprotonated by anode oxidation to provide the desired product **136’**. In mechanism B, on the other hand, removal of hydrogen from carboxylic acid (R-COOH) on the cathode or exclusion of proton through electro-generated CH_3_COO^–^ results in **III**, which is then oxidized to carboxyl radical **IV** by anode oxidation. The cyclized intermediate **V**, which is promptly aromatized through a single electron transfer and deprotonated by anode oxidation to yield the desired product **136**, is then attacked intramolecularly by a carboxyl radical. The concurrent cathodic process in both mechanisms A and B was lowering H^+^ to liberate hydrogen gas (Scheme 97).

**Scheme 98:**
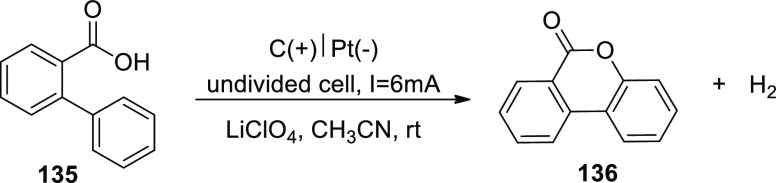
Electrosynthesis of 6*H*-Benzo[c]chromen-6-one **136**

In 2018, the electrosynthesis of aromatic lactones **136** via dehydrogenative cyclization is illustrated using a robust and reliable methodology based on electrochemical techniques (Scheme 98). This novel and beneficial electrochemical process is scalable to 100 g under modest parameters. Surprisingly, substrates containing heterocycles can be used, broadening efficiency of this process. Graphite and platinum were used as the anode and cathode, respectively, in the electrochemical C–O cyclization of 2-phenylbenzoic acid **135**. Applying 6 mA of a steady current the required product **136** was achieved in 91% yield at room temperature. Detailed illustration of this strategy is already described in part **B** of Scheme 97.^82^

**Scheme 99:**
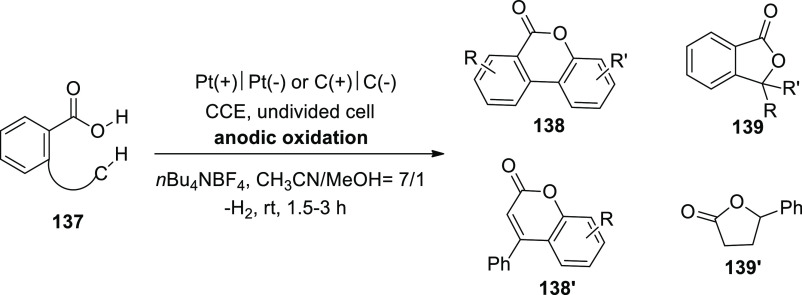
Electrosynthesis of Substituted Coumarin **138** and Tetrahydrofuranone **139**

In 2018, without using external oxidants or metals, a viable electrochemical approach for the direct lactonization is devised with outstanding regioselectivity. Under constant current scenarios, 2-biphenylcarboxylic acid **137** is cyclized easily. In this circumstance, tetrabutylammonium tetrafluoroborate (*n*Bu_4_NBF_4_) was employed as an electrolyte and mixture of methanol and methylcyanide as a solvent system in an undivided cell equipped with a Pt/C-anode and cathode. On a 40 g scale, the scalability of this freshly designed technique was proven using a straightforward method (Scheme 99).^83^

**Scheme 100:**
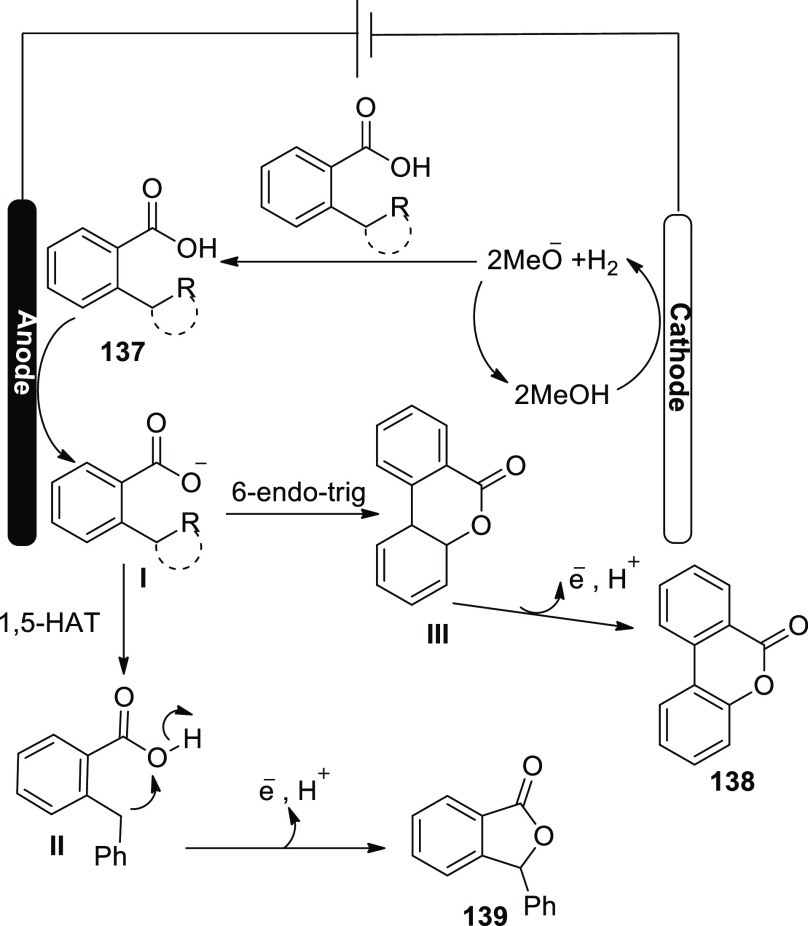
Proposed Mechanism for the Electrosynthesis of Substituted Coumarin **138** and Tetrahydrofuranone **139**

Details are offered in Scheme 100. The carboxylate radical **I** takes one of two routes, depending on the substrate structure. A 6-endo-trig cyclization yields intermediate **III**. When the C(sp^3^)–H bond is lactonized, hydrogen-atom transfer occurs, forming a stabilized benzyl radical **II**. Eventually, radicals **III** and **II** lose an electron and a proton to become products **138** and **139**, respectively.

**Scheme 101:**
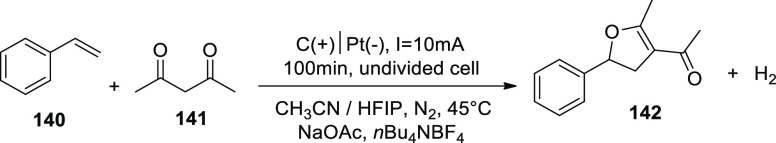
Electrosynthesis of Substituted Dihydrofuran **142**

In 2019, according to Xing and colleagues, dihydrofuran derivatives **129** were electrochemically formed (Scheme 101). Starting from styrene derivatives **140** and acetylacetone **141**, a large family of product **142** was formed in good yield. The reaction was put out in an undivided cell with carbon as the anode, platinum plate as the cathode, and CH_3_CN/HFIP as solvent/electrolyte. The constant current used was 10 mA.^84^

**Scheme 102:**
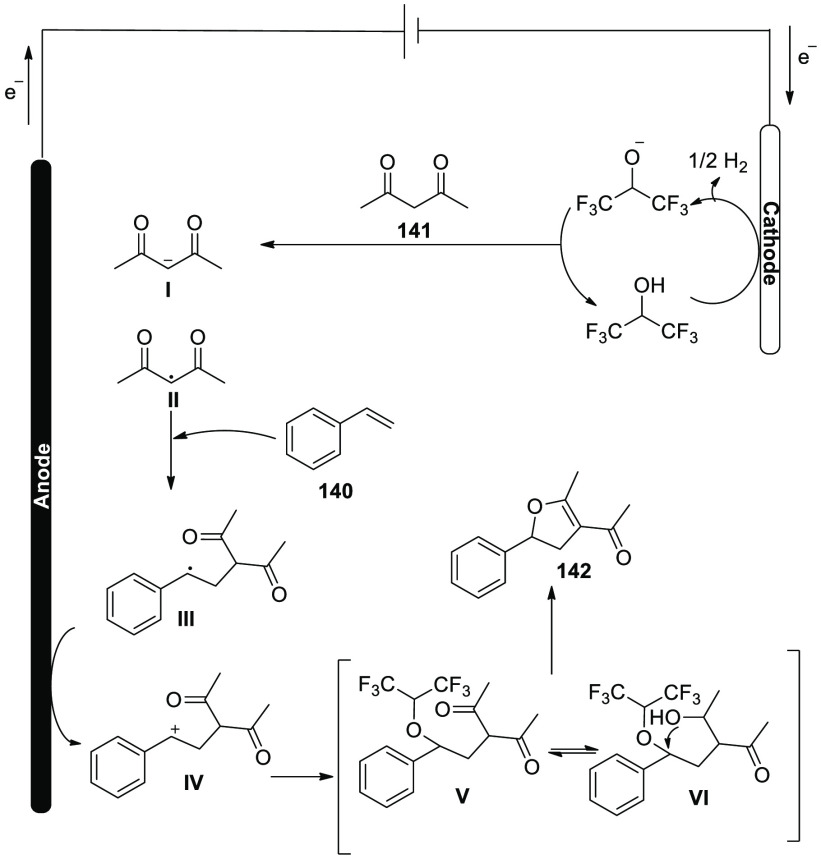
Proposed Mechanism for the Electrosynthesis of Substituted Dihydrofuran **142**

A mechanism has been suggested (Scheme 102). The acetylacetone anion **I** formed by deprotonation of acetylacetone **141** by the base, NaOAc or alkoxide, is oxidized to give trace amounts of the electrophilic C-radical **II**, which can be stabilized by HFIP (anodic event). The C-radical **II** then reacts with styrene **140** to form the benzylic radical **III**, which is then oxidized at the anode to produce the benzylic cation **IV**. The probable intermediate **V** tautomerizing to **VI**, which undertakes an intermolecular nucleophilic assault to liberate the molecular target **142**, can be further stabilized by the HFIP cluster.

**Scheme 103:**
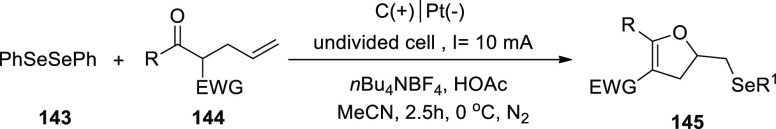
Electrosynthesis of Substituted Selenated Dihydrofuran **145**

In 2019, electrochemical oxidation of olefinic carbonyls **144** with freely accessible diselenides **143** has been successfully achieved, allowing for simultaneous development of C–Se and C–O links in an environmentally benign setup. In the beginning, the electrochemical oxidative cyclization reaction was started with model substrates **143** and **144** (Scheme 103). With graphite-rod anode (+) and platinum sheet cathode (−), the final product was achieved in good yield using *n*Bu_4_NBF_4_ (electrolyte), acetic acid (additive), and acetonitrile (solvent). With this clever chelation method, a variety of seleno-dihydrofurans **145** and selenooxazolines with brittle heterocycles, modest C–I bonds, and supernumerary vinyl groups were created. This transformation takes place avoiding external metallic and oxidizing molecules.^85^

**Scheme 104:**
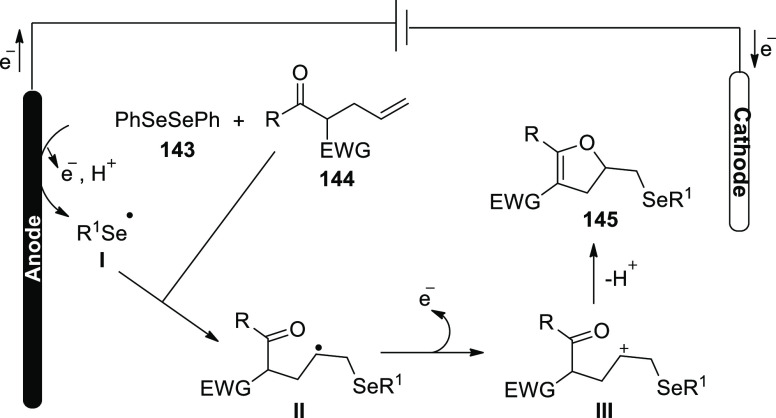
Proposed Mechanism for the Electrosynthesis of Substituted Selenated Dihydrofuran **145**

Scheme 104 displays the hypothesized pathway for this reaction. **144** interacts with phenylselenium radical **I** to generate the alkyl radical **II**, which then gives rise to the cation **III** after one electron is removed. Fast ring closure, followed by nucleophilic attack on the carbonyl oxygen atom and deprotonation, results in the production of the desired product **145**.

**Scheme 105:**
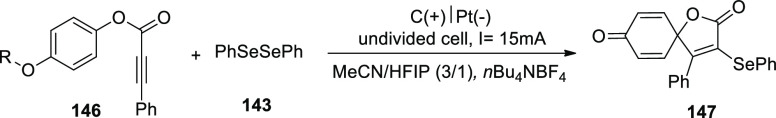
Electrosynthesis of 3-Selenated spiro[4.5]trienones **147**

In 2020, a new and environment friendly method for producing spiro[4.5]trienones has been devised (Scheme 105). This mildly regulated process was carried out in an undivided cell. Diphenyl diselenide **143** and 4-methoxyphenyl-3-phenylpropiolate **146** were selected as the model reaction to produce 3-selenated spiro[4.5]trienones **147** applying 15 mA-constant current, a platinum-cathode, and a graphite-anode. In CH_3_CN/HFIP, *n*Bu_4_NPF_6_ was used as an electrolyte, yielding 84% of the target product **147**. In addition, huge amounts of other homologous substances were generated.^86^

**Scheme 106:**
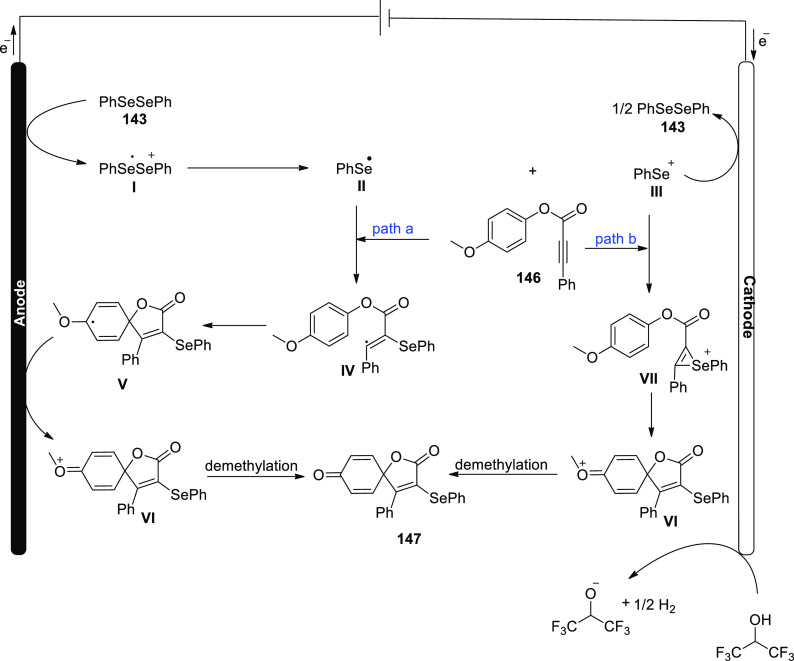
Proposed Mechanism for the Electrosynthesis of 3-Selenated spiro[4.5]trienones **147**

Scheme 106 suggests a potential reaction mechanism for the electrochemical transition. The cationic radical intermediate **I** produced by the first oxidation of diphenyl diselenide **143** is then broken down to produce radical **II** and cation **III**. Then, via radical addition of the C–C triple bond, phenylselenium radical **II** combines with **146** to create vinyl radical **IV**. Because of the five-membered ring structure and the relative stability of the resonant free radical, the vinyl radical **IV** usually completes the intramolecular spirocyclization to produce **V**. Additionally, intermediate **V** is further oxidized at the anode to produce intermediate **VI**, an oxygenium cation. Last but not least, the demethylation of cation **VI** and the dearomatization of aromatic ring produced the product **147** (path a). Alternately, we cannot completely rule out the idea that the intermediate **VII** is created when the phenyl selenium cation **III** combines with **146**. The cyclic selenium cation is then attacked intramolecularly by an aromatic ring with a high electron density, producing intermediate **VI**, which is then demethylated to produce the product **147** (path b).

**Scheme 107:**
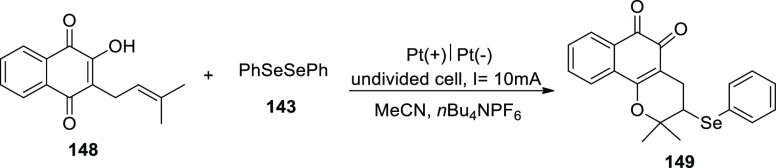
Electrosynthesis of Selenium-Containing Naphthoquinones **149**

In 2020, the production of selenium-containing naphthoquinones **149** is possible through electrochemical selenation in undivided electrochemical cells (Scheme 107). By switching from an electrolyte to *n*Bu_4_NPF_6_ and using a current of 10 mA and a reaction duration of 1 h, the quick, green, and effective methodology yields **149** (yield = 93%) in a quick and dependable manner in the absence of oxidizing agents.^87^

**Scheme 108:**
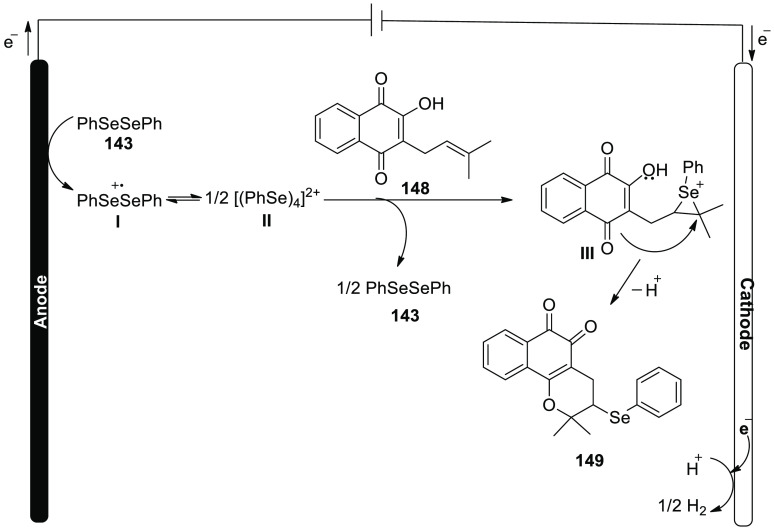
Proposed Mechanism for the Electrosynthesis of Selenium-Containing Naphthoquinones **149**

The proposed mechanism for the electrosynthesis ofselenium-containing naphthoquinones **149** is given in Scheme 108. Lapachol **148** was added, and a new anodic reaction was seen at *E*_p_ = 1.55 V vs SCE. This new anodic response can be attributed to the oxidation of product **149**, as shown by a separate analysis of product **149**. Both results—the additional anodic event and the declining cathodic event at higher scan rates—indicate that the selenium dication **II** and lapachol **148** quickly react carbophilically to formulate cationic intermediate **III** that quickly shows nucleophilic cyclization to produce product **149** and diselenide **143**.

**Scheme 109:**
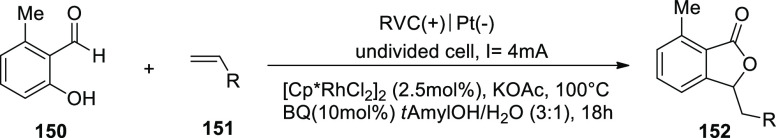
Electrosynthesis of Substituted Isobenzofuran-1(3*H*)-one **152**

In 2020, iridium catalyzed electrocatalysis was exhibited. This fabrication is an illustrative of user-friendliness, highly stereo- and chemo-selective (Scheme 109). Alkene **151** and 2-hydroxybenzaldehyde **150** combine to form substituted isobenzofuran-1(3*H*)-one **152**. Using potassium acetate as the electrolyte, a process occurs in an undivided cell at 100 °C for 18 h.^65,66^

**Scheme 110:**
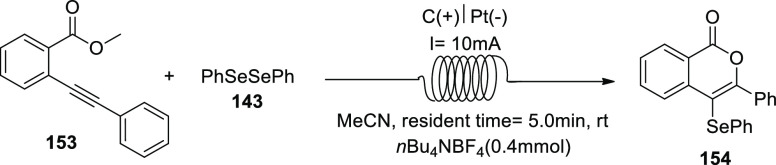
Electrosynthesis of Isocoumarin **154**

In 2020, a continuous electrochemical microreactor was employed to start an oxidative cyclization reaction between *o*-(1-alkynyl) benzoate and radicals. It is a nonoxidant and metal-free method of manufacturing isocoumarin **154** (Scheme 110). Even though the postprocessing technique has no longer undergone any green enhancements, the greening of this synthesis method still has a lot of opportunities for expansion due to the enhancement of the chemical effectiveness. Furthermore, gram scale up events may be accomplished using a continuous electrochemical formulation, and good yields of the desired product are acquired, demonstrating its significant potential for use in future industrial production. Methyl 2-(phenylethynyl)benzoate **153** and **143** are combined with two equivalents of *n*Bu_4_NBF_4_ and the solvent acetonitrile. The combined solution was pumped into the electrochemical microreactor for a reaction at a flow rate of 45 L/min and a current of 10 mA. And a yield of 92% can be achieved to produce the target product **154**.^88^

**Scheme 111:**
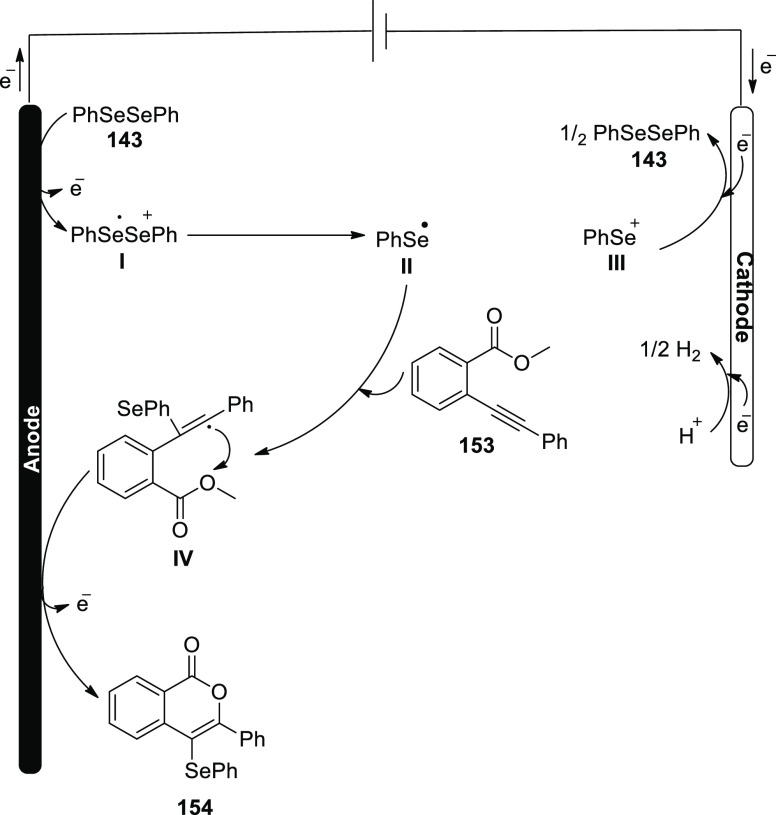
Proposed Mechanism for the Electrosynthesis of Isocoumarin **154**

The detailed mechanism is described by Scheme 111. First, at the anode, diphenyl selenide **143** is oxidized to a cationic radical intermediate **I**, which is then split into a phenyl-selenium radical **II** and a phenyl-selenium cation **III**, respectively. In preparation for the following cycle, phenyl-selenium cation **III** was reduced to diphenyl-selenide at the cathode, while phenyl-selenium radicals attacked the C–C triple bond of *o*-(1-alkynyl) benzoates **153** to produce the highly regioselective vinyl radical **IV**. The intramolecular cyclization of this in situ produced vinyl group is then carried out by interaction with the −OMe moiety, leading to the synthesis of the end product **154** and release of the methyl positive ion. The methyl cation subsequently captures the hydroxide anion (OH^–^) in the solvent to produce methyl alcohol.

In 2021, it was suggested that chromones can be assembled over time via electro-formyl C–H activation. An undivided cell with a graphite felt (GF) anode and a platinum cathode was used to conduct the electrocatalysis. A 10 mL cell was filled with the 2-hydroxybenzaldehyde **155**, alkyne **156**, NaOPiv (electrolyte), [Cp*RhCl_2_]_2_ (catalyst), and *t*-AmylOH/H_2_O (4.0 mL, 3:1) (solvent mixture). To produce the desired compounds **157**, electrocatalysis was carried out at 100 °C in undivided cell containing GF-anode and Pt-cathode with a continuous current of 4 mA.^89^

**Scheme 112:**
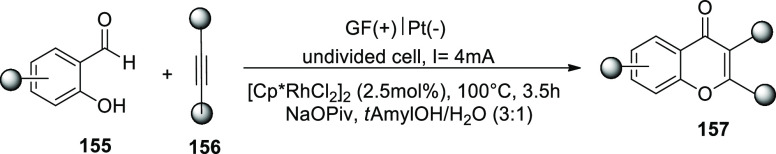
Electrosynthesis of 4*H*-Chromen-4-one **157**

**Scheme 113:**
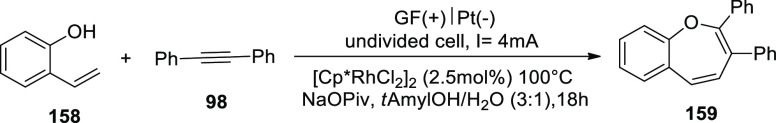
Electrosynthesis of Benzoxepine **159**

In 2021, metal-catalyzed [5 + 2] cycloadditions were described to create useful 7-membered benzoxepine **157** skeletons (Scheme 113). The effective alkyne annulation has a wide range of substrates and merely used electricity as an oxidant. Graphite felt and platinum plate were used as the anode and cathode materials in an undivided cell arrangement for electrochemical [5 + 2] cycloadditions employing 2-vinylphenol **158** and diphenylacetylene **98**. With pentamethylcyclopentadienyl rhodium dichloride dimer (catalyst) and NaOPiv(electrolyte) in tAmOH-H_2_O (solvent system) at 100 °C for 18 h, the target product **159** was isolated in 88% yield following extensive testing.^90^

**Scheme 114:**
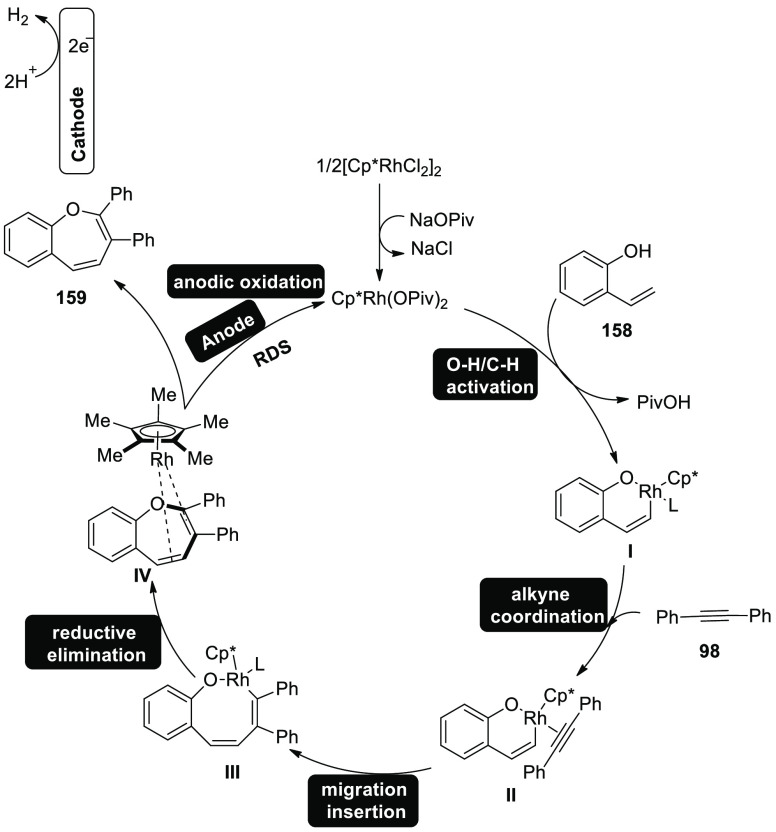
Proposed Mechanism for the Electrosynthesis of Benzoxepine **159**

A possible catalytic cycle is described (Scheme 114), which begins by the activation of oxygen–hydrogen or carbon–hydrogen bond to produce a rhodacycle **I**. The intermediate **III** is then produced by alkyne coordination–insertion, which is followed by a quick reductive-elimination to create complex **IV**. Ultimately, at the anode, the Rh^I^ is oxidized again to Rh^III^ releasing product **159** and producing molecular hydrogen at the cathode as a byproduct.

**Scheme 115:**
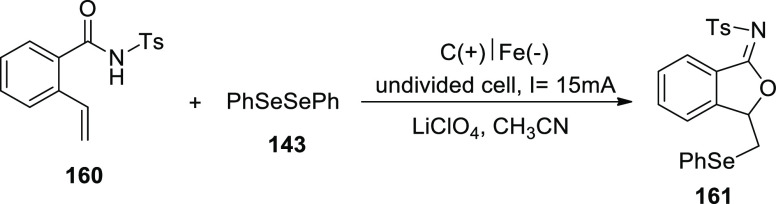
Electrosynthesis of Selenatedisobenzofuran **161**

Another process for manufacturing O-heterocycles was published by Bian and co-workers in 2021. The model reaction was the oxidation of N-tosyl-2-vinylbenzamide **160** and diphenyl diselenid e**143** in an electrochemical microreactor utilizing a graphite anode and an iron cathode to yield selenatedisobenzofuran **161**. First, different solvents were tested under a 15 mA constant current with LiClO_4_ as the electrolyte; the target iminoisobenzofuran product **161** was produced in good yield in CH_3_CN. After that, a number of supportive electrolytes were tested. Without utilizing oxidants and metals, this method allowed the formation of a wide quantity of iminoisobenzofuran derivatives in exceptional outcomes (Scheme 115). The continuous-flow system paired with electrochemical procedures solves the difficulty of scaling up a batch-type electrolysis.^91^

**Scheme 116:**
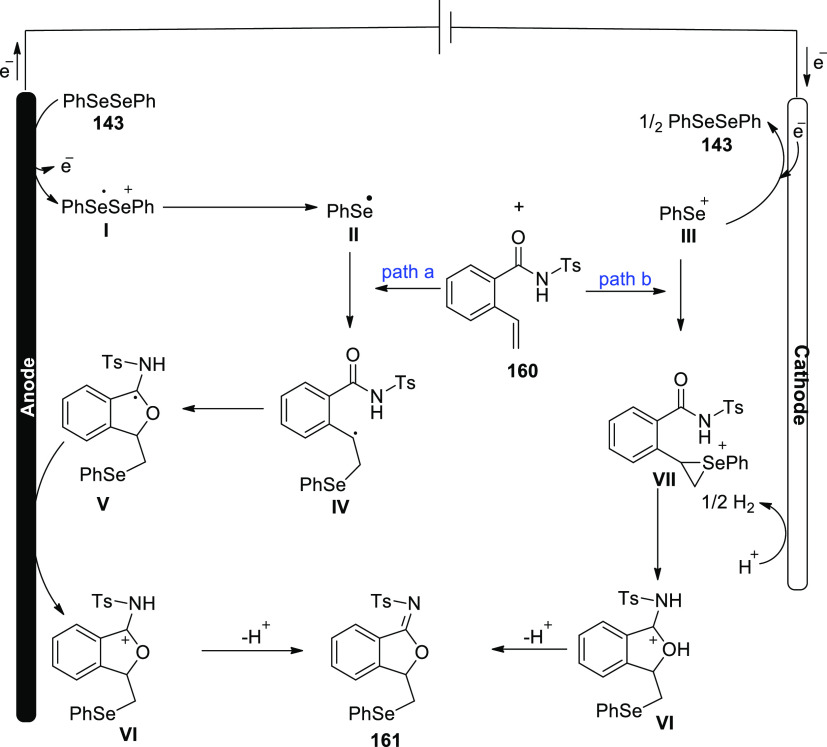
Proposed Mechanism for the Electrosynthesis of Selenatedisobenzofuran **161**

The concept of an electrochemical oxidative cyclization action has been explored (Scheme 116). At the anode, diphenyl diselenide **143** is oxidized to a cationic radical intermediate **I**, which is then decomposed into phenylselenium radical **II** and phenylselenium **III**, respectively. The alkyl radical **IV** is formed when phenylselenium radical **II** assaults the C=C of **160**. The alkyl radical **IV** is cyclized intramolecularly to produce **V**, which is then oxidized at the cathode to produce the intermediate **VI**. Eventually, the product **161** is obtained by dehydrogenating cation **VI** (path a). Furthermore, the potential that phenyl selenium cation **III** interacts with **160** to form intermediate **VII** cannot be ruled out. Then an intramolecular nucleophilic attack occurs, forming intermediate **VI**, which is then dehydrogenated to generate the target compound **161** (path b).

In 2018, 2020 and 2021, the hypothetical electrooxidative C–H transition was demonstrated with weakly coordinating benzoic acid **162**. The most effective way to produce the desired product **152** was using a *t*-AmOH and H_2_O solvent mixture with KOAc as the addition in an easy-to-use undivided cell arrangement. The total efficacy might be significantly increased by participation of ferrocene/*p*-benzoquinone (catalytic mediators) as compared with that of rhodium (Rh)^92^ and ruthenium (Ru) catalysis.^93,94^

**Scheme 117:**
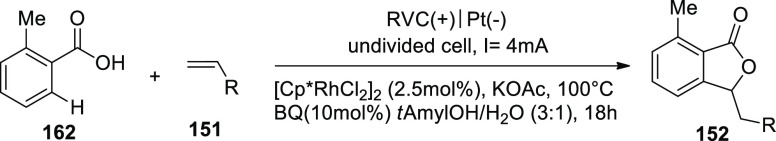
Electrosynthesis of Substituted Isobenzofuran-1(3*H*)-one **152**

**Scheme 118:**
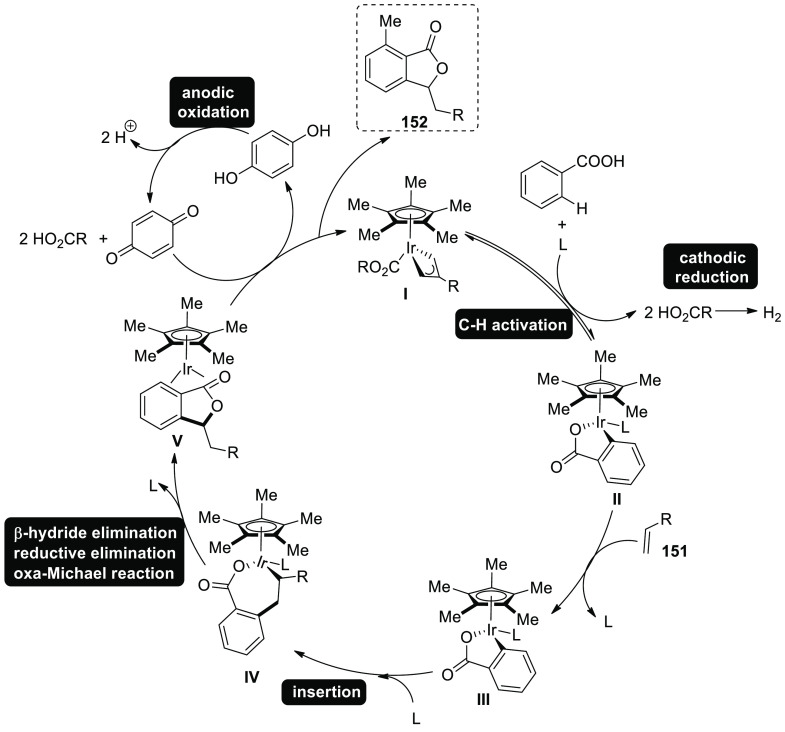
Proposed Mechanism for the Electrosynthesis of Substituted Isobenzofuran-1(3*H*)-one **152**

In 2018, 2020, and 2021, likely, an iridium(Ir)-catalyzed cycle is initiated for easy organometallic C–H functionalization (Scheme 118). 7-membered iridium(III) cycle **IV** is then produced by migrating alkene **154** insertion. This cycle then proceeds through reductive elimination and β-hydride to produce intermediate **V**. With the help of *p*-benzoquinone, intermediate **V** is reoxidized to create the catalytically active compound **I**. Finally, anodic oxidation is taking place on the newly generated hydroquinone.^65,66,88,93,95^

**Scheme 119:**
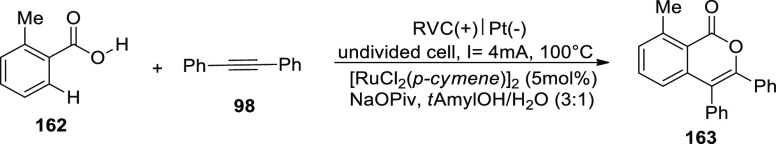
Electrosynthesis of Substituted 1*H*-Isochromen-1-one **163**

In 2018 and 2021, a ruthenium catalyzed approach was first reported (Scheme 119). The only oxidant used was electricity to activate the C–H bond. Hydrogen gas was released as waste. Water and air had no effect on the catalyst used in this process. Best results were acquired in a solvent solution of *tert*-amyl alcohol (*t*AmOH) and water (H_2_O) with the addition of sodium pivalate (NaOPiv). Biphenylacetylene **98** and benzoic acid **162** are transformed into substituted 1*H*-isochromen-1-one **163** in the presence of these circumstances.^88−90^

**Scheme 120:**
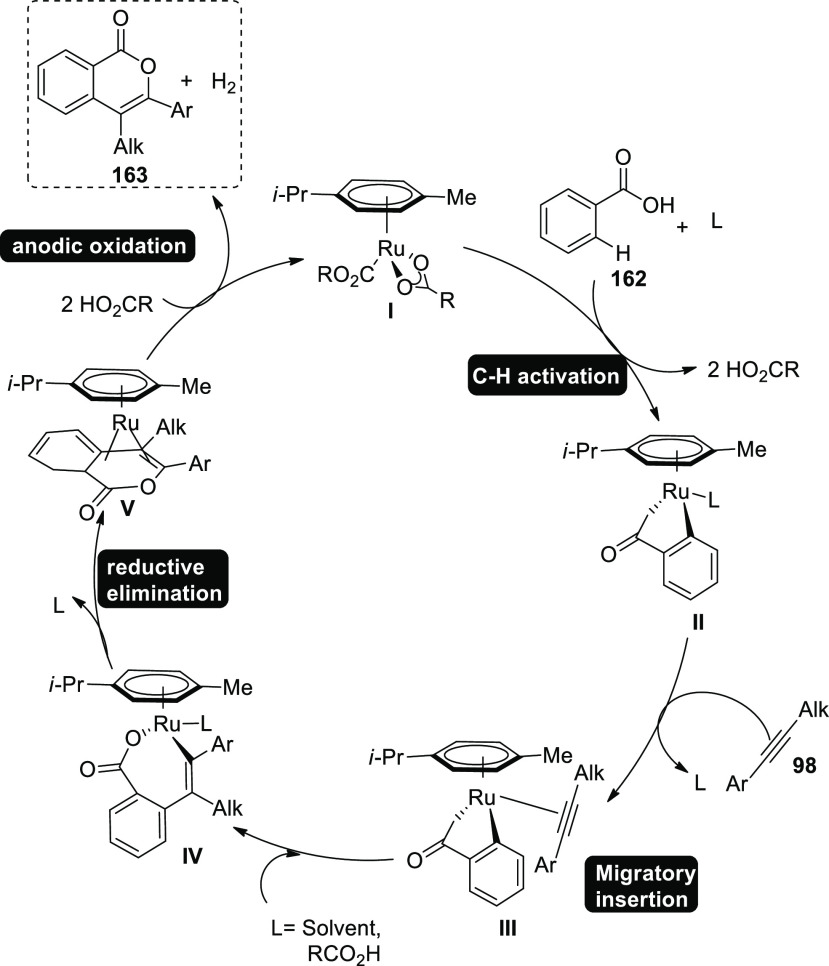
Proposed Mechanism for the Electrosynthesis of Substituted 1*H*-Isochromen-1-one **163**

It is suggested that a realistic catalytic cycle start with an easy organometallic C–H activation (Scheme 120). As a result, the ruthena(II) cycle **II** and two equivalents of carboxylic acid are produced. **IV** is subsequently provided by migrating alkyne insertion. This cycle quickly passes through reductive-elimination to produce complex **V**. Anodic-oxidation completes reoxidation complex **I**.

## 3 Conclusion and Future Perspectives

The focus of this review is on significant innovations in electrochemical synthesis of N-, S-, O-heterocyclic compounds. There is also discussion of the potential heterocycle formation mechanisms. These families of compounds have significant utility especially in drug synthesis, therefore, the development of sustainable synthetic routes is prerequisite. The discovery of the organic electrochemistry was started in the 19th century, and is still an underdeveloped research area. Electrosynthesis is an enabling technique that allows for selective construction of N-, S-, O-heterocyclic compounds. This method offers a milder condition, reduces the number of steps, and provides alternative routes compare to classical synthesis. The processes involving redox reactions are suitable for electrolysis because an oxidation reaction at the anode and a reduction reaction at the cathode occur simultaneously during electrolysis.

The use of electricity^96^ as an alternative constitutes a convenient, atom- and cost-efficient way to form molecules. Despite the fact that this field of research has made significant progress, some obstacles still exist, such as the requirement for huge quantities of supportive electrolytes. Utilizing continuous-flow electrochemistry in electrolyte-free environments is one of the directions in this field when considering the trend toward green synthesis.^97^ Additionally, the recent fusion of photochemistry and electrosynthesis allowed for homogeneous photoelectrocatalysis with exceptional redox potentials, removing the need for harsh chemical redox reagents.^98^

However, cathodic heterocycle production appears to be an uncommon occurrence, with anodic oxidation accounting for the majority of the electrochemical heterocyclic syntheses documented so far. Naturally, this indicates that there are many uncharted areas and prospects for further research in this area. The field of electroorganic synthesis is expanding, and it is presumed that new advancements in electroreductive techniques will continue to enrich it and offer useful solutions to synthetic problems.^99^ More intriguing developments are envisioned, such as the application of renewable solvents and renewable energy sources.^100^ It is fair to assume that the application of new processes in bioderived reaction media will progress in a manner that is synchronous with the development of novel heterocyclic reactions.^101^ It is possible to move on with additional developments for anodic oxidation-based transition metal recycling.^41^ Regio- and stereoselective bond functionalizations will gain a new dimension with the development of suitable catalyst designs in the future.^102^
